# Outstanding CO_2_ Photoreduction in Single‐Atom Thulium Modified Carbon Nitride

**DOI:** 10.1002/advs.202406329

**Published:** 2024-08-09

**Authors:** Cheng Ding, Liuqing Yang, Xinxin Lu, Haoqiang Chi, Yong Yang, Junyang Yuan, Xiaoyong Wang, Xinglong Wu, Yongcai Zhang, Yong Zhou, Zhigang Zou

**Affiliations:** ^1^ Key Laboratory of Modern Acoustics (MOE) Institute of Acoustics School of Physics National Laboratory of Solid‐State Microstructures College of Engineering and Applied Sciences Collaborative Innovation Center of Advanced Microstructures Eco‐Materials and Renewable Energy Research Center (ERERC) Jiangsu Key Laboratory for Nano Technology Nanjing University Nanjing Jiangsu 210093 P. R. China; ^2^ College of Science Nanjing Forestry University Nanjing Jiangsu 210037 P. R. China; ^3^ Kunshan Sunlaite New Energy Co. Ltd. Kunshan Innovation Institute of Nanjing University No. 1666 South Zuchongzhi Road Kunshan Jiangsu 215347 P. R. China; ^4^ PetroChina Shenzhen New Energy Research Institute Shenzhen Guangdong 518052 P. R. China; ^5^ Key Laboratory of Soft Chemistry and Functional Materials (MOE) Nanjing University of Science and Technology Nanjing Jiangsu 210094 P. R. China; ^6^ School of Chemistry and Chemical Engineering Yangzhou University Yangzhou 225009 P. R. China; ^7^ School of Science and Engineering The Chinese University of Hongkong (Shenzhen) Shenzhen Guangdong 518172 P. R. China; ^8^ School of Chemical and Environmental Engineering Anhui Polytechnic University Wuhu Anhui 241000 P. R. China

**Keywords:** carbon vacancies, g‐C_3_N_4_, photocatalytic CO_2_ reduction, single atom, thulium

## Abstract

CO_2_ reduction photocatalysts are favorable for obtaining renewable energy. Enriched active sites and effective photogenerated‐carriers separation are keys for improving CO_2_ photo‐reduction. A thulium (Tm) single atom tailoring strategy introducing carbon vacancies in porous tubular graphitic carbon nitride (g‐C_3_N_4_) surpassing the ever‐reported g‐C_3_N_4_ based photocatalysts, with 199.47 µmol g^−1^ h^−1^ CO yield, 96.8% CO selectivity, 0.84% apparent quantum efficiency and excellent photocatalytic stability, is implemented in this work. Results revealed that in‐plane Tm sites and interlayer‐bridged Tm‐N charge transfer channels significantly enhanced the aggregation/transfer of photogenerated electrons thus promoting CO_2_ adsorption/activation and contributing to *COOH intermediates formation. Meanwhile, Tm atoms and carbon vacancies both benefit for rich active sites and enhanced photogenerated‐charge separation, thus optimizing reaction pathway and leading to excellent CO_2_ photo‐reduction. This work not only provides guidelines for CO_2_ photo‐reduction catalysts design but also offers mechanistic insights into single‐atom based photocatalysts for solar fuel production.

## Graphical abstract

Single‐atom thulium (Tm) modified porous tubular carbon nitride with carbon vacancies is successfully designed and synthesized. The construction of in‐plane Tm sites, interlayer‐bridged Tm‐N charge transfer channels, and carbon vacancies promoted the transfer/separation of photogenerated carriers, enhanced the CO_2_ adsorption/activation, optimized the reaction pathway, and ultimately led to excellent CO_2_ photo‐reduction.

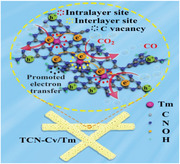

## 1 Introduction

CO_2_ photo‐reduction for fuels/value‐added chemicals has been recognized as an approach for obtaining renewable energy as well as solving energy shortages and global environmental problems.^[^ ^1^ ^]^ Single‐atom catalysts (SACs) with the maximal active metal utilization, came into scientists’ eyes due to their accessibility of active sites as well as excellent catalytic performance and product selectivity for CO_2_ photo‐reduction.^[^ ^2^ ^]^ However, the stability of SACs is still far from satisfactory due to the weak interactions between metal atoms and dispersion media which lead to the tendency to aggregate into thermodynamically stable nanoparticles.^[^ ^3^ ^]^

We chose graphitic carbon nitride (g‐C_3_N_4_), which has received widespread attention in the field of photocatalysis on account of its low cost, facile preparation, and excellent chemical stability, as a single atom dispersion support.^[^ ^4^ ^]^ Due to the ultra‐high content of Lewis‐base pyridine nitrogen which can efficiently interact with the strong Lewis‐acid metal atoms to form a metal‐nitrogen coordination structure, g‐C_3_N_4_ with specific functionalities for stabilizing metal atoms, was regarded as a promising single‐atom carrier.^[^ ^3^, ^5^ ^]^ Some single‐atom doping g‐C_3_N_4_, for instance, erbium doping g‐C_3_N_4_ with 47.1 µmol g^−1^ h^−1^ CO generation^[^ ^6^ ^]^ and Cu single‐atom supported on g‐C_3_N_4_ with CO yield of 3.086 µmol g^−1^ h^−1^,^[^ ^7^ ^]^ introduced metal single atoms into π‐conjugated planes and increased the in‐plane conductivity of g‐C_3_N_4_, favoring in‐plane charge transfer. However, charge transport between adjacent layers in g‐C_3_N_4_ failed to be improved. Interlayer charge transfer was concerned in lanthanum (La) or Cu single‐atom doped g‐C_3_N_4_ through the formation of La‐N or Cu‐N(C) charge‐transfer channels between La or Cu single‐atom and adjacent layers of g‐C_3_N_4_.^[^ ^5^, ^8^ ^]^ However, rare studies are able to consider both intra‐plane and interlayer co‐modifications to enhance the separation/transfer of photogenerated charge carriers for g‐C_3_N_4_‐based SACs. Moreover, in order to overcome the inherent disadvantage that van der Waals force is unfavorable to the homogeneous loading of metal atoms in g‐C_3_N_4_,^[^ ^9^ ^]^ we chose g‐C_3_N_4_ with porous tubular morphology as the single atom carrier. This porous tubular g‐C_3_N_4_ is not only conducive to the provision of sufficient load‐bearing sites for metal atoms but also facilitates the adsorption of reactants and the transport of gas molecules.^[^ ^10^ ^]^ Therefore, we designed a simple strategy to obtain single‐atom modified porous g‐C_3_N_4_ nanotubes to achieve full dispersion of single atoms and separation of intra‐plane/interlayer photogenerated charges.

The choice of single‐atom species directly affects the activity of CO_2_ photo‐reduction and on the other hand, defect engineering of intrinsic molecular structure is beneficial for enhancing catalytic activity. However, most of the designs only focus on introducing foreign active metal sites to capture photogenerated electrons to activate CO_2_, e.g., single‐atom Pt‐anchored defective carbon nitride,^[^ ^11^ ^]^ Ni single‐atom decorated boron‐oxo‐species‐modified g‐C_3_N_4_^[^ ^12^ ^]^ and single‐atom Ru‐modified CdS.^[^ ^13^ ^]^ Otherwise, recent studies showed that carbon vacancies in g‐C_3_N_4_ can trap photoinduced electrons, facilitate photogenerated carriers transfer/separation and enhance CO_2_ adsorption/activation.^[^ ^14^ ^]^ We infer that using single atom modification to achieve shear modification while increasing active sites and carbon defects will greatly benefit CO_2_ photoreduction in g‐C_3_N_4_, but this topic is rarely studied.

To design SACs that integrate single‐atom metal sites and carbon vacancy and to explore the structure‐performance relationship for a deep understanding of CO_2_ photo‐reduction, thulium (Tm) atoms which as presented electron capture/conduction ability in photocatalysts attracted our attention.^[^ ^15^ ^]^ Moreover, lanthanide chemistry studies have revealed that the fz^3^ orbital of the 4f orbitals or the dz^2^ orbital of the 5d orbitals of rare‐earth elements are closest to the Fermi surface of the material, leading to the fact that rare‐earth elements doping contributes to the enhancement of the electrons transfer in the interlayer toward the Z‐direction.^[^ ^5^, ^16^ ^]^ Thus, these provide the feasibility of introducing Tm single atoms in g‐C_3_N_4_ to enhance intra‐plane/interlayer photogenerated carrier separation/transfer. In addition, Tm sites may increase the adsorption of CO_2_ by the evidence that the possible generation of an electric field between unsaturated Tm atoms and π‐electrons would induce dipoles in CO_2_ as well as the interaction between the quadrupole moment of CO_2_ and the electric field gradient in Tm complexes.^[^ ^17^ ^]^ More importantly, Tm species exhibit high activity in reduction chemistry, such as the reductive conversion of CO_2_.^[^ ^18^ ^]^ Moreover, since the 4f orbitals of rare‐earth atoms are completely shielded by octet electrons of the outer orbitals (5s^2^p^6^), Tm atoms have no tendency to interact with H_2_O molecules thus exhibiting hydrophobicity properties.^[^ ^19^ ^]^ Comprehensively analyzing the above factors, there is a high probability of excellent CO_2_ photoreduction in single‐atom Tm modified porous tubular carbon nitride with carbon vacancies. However, this kind of Tm single‐atom decorated g‐C_3_N_4_ for CO_2_ photo‐reduction has not been reported.

We explored a facile template‐free method to prepare porous tubular g‐C_3_N_4_ (TCN) as a single‐atom support and an intra‐/interlayer co‐confined Tm single‐atom decorated TCN containing carbon vacancies (TCN‐Cv/Tm‐Y, Y = 1, 2, or 3, representing the Tm content) were originally synthesized. Moreover, it was surprisingly found that TCN‐Cv/Tm‐Y exhibited a highly efficient photo‐reduction from CO_2_ to CO without any co‐catalysts or sacrificial agents. The optimized TCN‐Cv/Tm‐2 achieved 199.47 µmol g^−1^ h^−1^ CO yield and 0.84% apparent quantum efficiency, which is not only ≈16.75‐fold and 3.33‐fold higher than that of bulk g‐C_3_N_4_ (CN) and pristine TCN but also significantly better than most g‐C_3_N_4_‐based photocatalysts. Results show that the introduction of intra‐/interlayer Tm^δ+^ sites and carbon vacancies significantly enhanced the photogenerated carriers’ transfer/separation. The intra‐/interlayer Tm sites effectively promoted CO_2_ adsorption/activation and significantly reduced the formation energy of *COOH. Meanwhile, carbon vacancy further lowered the activation energy barrier of CO_2_. Interlayer Tm‐N charge transfer channels lead to stronger activity for accelerating the formation of activated *CO_2_ species in interlayer Tm sites than in intralayer Tm sites. Therefore, the construction of inter‐/intralayer Tm sites and the accompanying carbon vacancies synergistically optimize the reaction path and significantly enhance the CO_2_ photo‐reduction activity. This work not only provides guidelines for CO_2_ photo‐reduction catalysts design but also offers mechanistic insights into single‐atom based photocatalysts for solar fuel production.

## 2 Results and Discussion

### 2.1 Synthesis and Characterizations

Tubular g‐C_3_N_4_ (TCN) was prepared using a facile template‐free one‐step annealing method (Figure S1, Supporting Information). No template was utilized, avoiding the tedious etching, and washing steps. Moreover, it was found that different pressures were applied to the precursor, resulting in different morphologies for the products, ultimately leading to significant differences in photo‐reduction CO_2_ performance. By fine‐tuning the pressure and evaluating its performance, the optimal product structure, namely TCN, was obtained. The optimization of morphology and structure will be detailed in the performance evaluation section.

**Figure 1:**
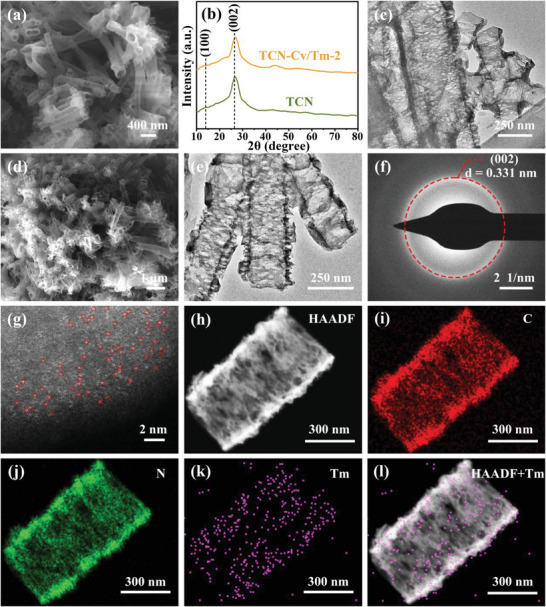
Structural characterizations of TCN and TCN‐Cv/Tm‐2. a) SEM image of TCN. b) XRD patterns. d–f) SEM, TEM, and SAED images for TCN‐Cv/Tm‐2. c) TEM image of TCN. g–l) AC HAADF‐STEM (single‐atom Tm species are highlighted by red circles) and EDX mapping images of TCN‐Cv/Tm‐2.

Scanning electron microscope (SEM) showed that TCN has a hollow porous tubular structure (**Figure** **1**a; Figure S2a, Supporting Information). The X‐ray diffraction (XRD) confirmed that the TCN exhibits a graphitic carbon nitride (g‐C_3_N_4_) crystal structure, with characteristic peaks near 13.9° and 27.0°, which are attributed to the (100) and (002) crystal planes of g‐C_3_N_4_, respectively (Figure 1b).^[^ ^20^ ^]^ Transmission electron microscope (TEM) further confirmed the porous tubular morphology of TCN (Figure 1c; Figure S2b, Supporting Information). The selected area electron diffraction (SAED) (Figure S2c, Supporting Information) was also collected, the (002) crystalline plane with interplanar spacing ≈0.34 nm can be obviously observed for TCN. It is further confirmed that TCN belongs to the g‐C_3_N_4_ crystal structure. Meanwhile, the uniform distribution of N and C in TCN was confirmed by energy‐dispersive X‐ray spectroscopy (EDS) and X‐ray photoelectron spectroscopy (XPS) (Figures S2a and S3a–d, Supporting Information). Furthermore, the N 1s high‐resolution XPS spectrum (Figure S3b, Supporting Information) proved a high 72.84% content of pyridinic nitrogen, which could be conducive to capturing Tm atoms by forming Tm─N bonds through its extra pair of electrons. As a reference, bulk g‐C_3_N_4_ (CN) was also synthesized using the same method as TCN without applying pressure. Structure analyses displayed CN has a similar crystalline structure and composition as TCN, but an obviously different geometry (Figure S4, Supporting Information).

The tubular g‐C_3_N_4_ (TCN‐Cv/Tm‐Y, Y = 1, 2, or 3, representing different Tm contents) catalysts containing intra‐/interlayer single‐atom Tm and C‐vacancies were synthesized through a facile template‐free impregnation and re‐annealing method (Figure S1, Supporting Information). The ingenuity of this reported method lies in: on one hand, the post‐embedded Tm atoms on g‐C_3_N_4_ nanotubes can effectively solve the problem of invalid reaction sites or waste of metal resources caused by encapsulation or aggregation compared with the common one‐pot pyrolysis method, thus improving the utilization of rare‐earth Tm, and significantly increasing the exposure amount of Tm active sites; on the other hand, the nano‐confinement effect based on a porous tubular structure can effectively prevent the aggregation of metal atoms during high‐temperature annealing, thereby uniformly and stably chelating Tm atoms. Furthermore, the actual Tm contents of the obtained photocatalysts were also measured by an inductively coupled plasma‐atomic emission spectrometer (ICP‐OES), 1, 2, and 3 corresponding to Tm contents of 0.79, 1.65, and 2.56 wt.%, respectively (Table S1, Supporting Information).

All the samples maintain a typical XRD spectrum of TCN (Figure 1b; Figure S5, Supporting Information), indicating that the introduction of Tm sites has not altered the crystal structure of TCN. No characteristic peaks corresponding to Tm‐related species such as Tm nanoparticles or Tm_2_O_3_ were detected in TCN‐Cv/Tm‐Y, suggesting that Tm may be evenly distributed on the TCN matrix in the form of single atoms by bonding with adjacent N or C atoms. It could be conducive to reducing the electronic localization and expanding the π‐conjugated system, promoting electron transfer, and thus facilitating the photocatalytic reduction of CO_2_.^[^ ^21^ ^]^ Compared to pure TCN, the weaker peak intensity and smaller (002) peak angle shift of TCN‐Cv/Tm‐Y were observed, which may be attributed to the introduction of C vacancies and intra‐/interlayer Tm atoms leading to the destruction of in‐plane long‐range ordering, decreased interlayer spacing, and increased distortion of interlayer stacking arrangement.^[^ ^3^, ^22^ ^]^

As observed in SEM, TEM, and SAED images (Figure 1d–f; Figure S6, Supporting Information), TCN‐Cv/Tm‐Y maintains the porous tubular morphology and structure of TCN. To clarify whether Tm was successfully introduced into the TCN matrix as isolated atoms, the distribution state of Tm sites on the TCN‐Cv/Tm‐2 catalyst was observed using aberration‐corrected high‐angle annular dark‐field scanning transmission electron microscopy (AC HAADF‐STEM). The presence of single‐atom bright spots corresponding to Tm (marked with red circles) and the absence of agglomeration can be clearly seen in the AC HAADF‐STEM images of TCN‐Cv/Tm‐2 (Figure 1g; Figure S7, Supporting Information), indicating that the Tm sites are indeed existing as single atoms on the TCN substrate without any metallic Tm nanoparticles or clusters. This is consistent with the XRD pattern where there are only TCN‐related diffraction peaks. The corresponding elemental mapping results for TCN‐Cv/Tm‐2 also confirm that C, N, and Tm are uniformly distributed throughout the porous tubular skeleton (Figure 1h–l).

**Figure 2:**
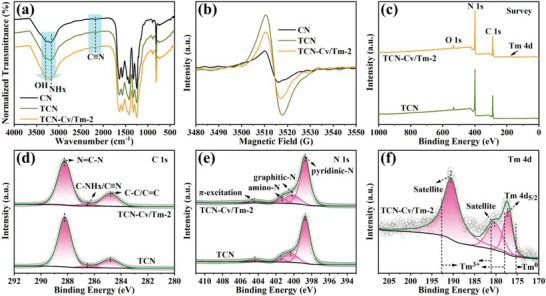
a) FTIR and b) EPR spectra of CN, TCN, and TCN‐Cv/Tm‐2 samples. c–f) XPS survey and high‐resolution spectra of TCN and TCN‐Cv/Tm‐2 samples ((c) Survey, (d) C 1s, (e) N 1s, and (f) Tm 4d).

The interior molecular structures of synthetic photocatalysts were revealed by Fourier transform infrared spectroscopy (FTIR) (**Figure** **2**a; Figure S8, Supporting Information). All samples showed typical infrared spectra of g‐C_3_N_4_, which further indicated that the synthesized catalysts retained the basic g‐C_3_N_4_ atomic structures. In detail, the sharp peak at 813 cm^−1^ can be attributed to the stretching vibration of the out‐of‐plane bending mode of the heptazine rings, the absorption peak at 890 cm^−1^ corresponding to the deformation mode of N─H, a series of strong adsorption peaks in the range of 1200–1700 cm^−1^ are derived from the stretching vibration modes of the N═C─N heterocyclic rings, and the two broad peaks in the region of 3100–3500 cm^−1^ are assigned to the stretching vibration modes of the N─H and O─H bands, related to the amino structure NHx (x = 1, 2) and surface adsorbed H_2_O molecules of the samples, respectively.^[^ ^20^, ^23^ ^]^ In addition, compared to the bulk CN, the tubular catalyst TCN has a new peak at 2175 cm^−1^, which is ascribed to the stretching vibration of C≡N.^[^ ^24^ ^]^ Notably, TCN‐Cv/Tm‐Y shows a significant increase in the N‐H peak intensity between 3100–3300 cm^−1^ after Tm single atoms were embedded in TCN, while the C≡N peak remains and the intensity almost unchanged. These results, therefore, suggest that the introduction of Tm single atoms did not affect the C≡N structure, but the resulting increased concentration of N‐H components may imply a loss of carbon in the heptazine unit, which is accompanied by the formation of C vacancies.^[^ ^25^ ^]^ Furthermore, as the Tm content gradually increases, the concentration of the N‐H also gradually rises, suggesting that the C vacancies may also be gradually increasing in content.

To confirm the formation of C vacancies, the electron paramagnetic resonance (EPR) spectra of the photocatalysts were measured (Figure 2b; Figure S9, Supporting Information). All samples exhibit a single Lorentzian line with a *g* value of ≈2.0036, which is generated by unpaired electrons on sp^2^‐hybridized carbon atoms in the π‐conjugated aromatic rings, indicating the presence of a similar g‐C_3_N_4_ skeleton structure.^[^ ^3^, ^26^ ^]^ However, the formation of the C≡N leads to a significant enhancement of the EPR signal intensity of TCN compared to the pristine CN (Figure 2b). This is mainly attributed to the strong electron‐withdrawing properties of the C≡N, thus allowing the isolated valence electrons in the π‐conjugated heterocyclic rings to be delocalized and concentrated.^[^ ^24^ ^]^ Notably, the EPR signal intensity of TCN‐Cv/Tm‐Y was significantly weakened after the introduction of the Tm single‐atom sites. Combined with the conclusion from FTIR analysis that the C≡N structure and content are not altered by modification of the Tm sites, the above phenomenon indicates a reduction in the lattice carbon content of TCN‐Cv/Tm‐Y. Thus, this also confirms the formation of C vacancies while anchoring Tm single atoms in the TCN matrix by H_2_/Ar thermal treatment.^[^ ^22^, ^26^ ^]^ Moreover, the EPR signal intensity gradually decreased with the gradual increase in Tm content (Figure S9, Supporting Information), indicating that the concentration of C vacancies was also gradually rising, which is highly consistent with the results of FTIR analysis.

To further reveal the elemental compositions and chemical states after the introduction of Tm single atoms and C vacancies into TCN, XPS analysis was conducted. Figure 2c and S10a (Supporting Information), show the constituents Tm, N, C, O of TCN‐Cv/Tm‐Y and N, C, O of TCN. In the C 1s high‐resolution XPS spectra (Figure 2d; Figure S10b, Supporting Information), it can be seen that the C 1s state displays three characteristic peaks at the binding energies of 288.2, 286.5, and 284.8 eV, respectively, where, the peak at 288.2 eV corresponds to the sp^2^‐hybridized carbon coordination in the N‐containing aromatic rings (N═C─N), and the peak at 284.8 eV represents adventitious carbon (C─C/C═C).^[^ ^22^, ^24^, ^27^ ^]^ In addition, the peak at 286.5 eV is attributed to C‐NHx (x = 1, 2) or C≡N groups on the edges of the heptazine units, which is mainly due to the C≡N groups and C‐NHx groups possessing similar binding energies.^[^ ^24^, ^27^, ^28^ ^]^ Notably, with the increase of Tm single atoms content, the peak area ratio of N═C─N to adventitious carbon decreased from 4.69 (TCN) to 2.71 (TCN‐Cv/Tm‐3) and the content of N═C─N decreased from 0.7991 (TCN) to 0.6436 (TCN‐Cv/Tm‐3), while the total content of C‐NHx and C≡N increased from 0.0305 (TCN) to 0.1192 (TCN‐Cv/Tm‐3) (Table S2, Supporting Information), also according to FTIR analyses showing that the C≡N groups are unaffected by modification of the Tm single‐atom sites, and thus this phenomenon suggests that C vacancies were formed in the sp^2^‐hybridized carbon in the N‐containing aromatic rings.^[^ ^22^, ^25^ ^]^ The N 1s high‐resolution XPS spectra (Figure 2e; Figure S10c, Supporting Information) could be deconvoluted into four peaks centered at the binding energies ≈398.7, 400.3, 401.4, and 404.4 eV, corresponding to the sp^2^ type N forming the two‐coordinated N atoms structure inside the triazine rings (C─N═C, pyridinic‐N), the three‐coordinated N atoms bonding structure between units (N‐C_3_, graphitic‐N), the C─N single bond linkage forming the C‐NHx amino structure (NHx, x = 1, 2), and the positive charge localization structure in heptazine rings (π‐excitation), respectively.^[^ ^25^, ^26^, ^29^ ^]^ Compared to TCN, with a gradual augmentation in Tm single‐atom sites loadings, the peak area of C─N═C decreased from 0.7284 (TCN) to 0.6668 (TCN‐Cv/Tm‐3), but the peak area of NHx increased from 0.0426 (TCN) to 0.1069 (TCN‐Cv/Tm‐3), meanwhile, the peak area ratio of NHx to N‐C_3_ raised from 0.23 (TCN) to 0.57 (TCN‐Cv/Tm‐3) (Table S3, Supporting Information). Of note, the increase in the NHx concentration and the decrease in the C─N═C content can be attributed to the loss of the carbon atoms, thus further confirming the formation of the C vacancies, which is consistent with the analysis of the C 1s spectra.^[^ ^25b^ ^]^ The formed C‐vacancies are surrounded by N‐dangling bonds (unsaturated N atoms), and simultaneously the N‐dangling bonds are compensated by adsorbed H, thus resulting in the formation of the NHx group.^[^ ^22a^ ^]^ Besides FTIR, EPR, and XPS, organic elemental analysis (OEA) was also employed to verify the formation of carbon vacancies. Compared with pure TCN, the C content, C/N and N/H molar ratios of TCN‐Cv/Tm‐Y decreased significantly with the introduction of Tm single atoms, while the H content increased significantly (Table S4, Supporting Information). Moreover, the N content is almost constant. These results not only further confirm the formation of C vacancies, but also demonstrate the absence of N vacancies in the synthetic materials. More importantly, previous studies have shown that the introduction of C vacancies could enhance the conductivity and mobility of photogenerated charge carriers, trap photo‐induced electrons, and promote charge separation, therefore, the introduction of C vacancies may contribute to improving photocatalytic activity.^[^ ^9^, ^26^, ^30^ ^]^

The high‐resolution Tm 4d state exhibits three signal peaks at the binding energies of 177.2 as well as at 180.3 and 190.5 eV, which should be attributed to the Tm 4d_5/2_ and satellite peaks, respectively (Figure 2f; Figure S1d, Supporting Information).^[^ ^31^ ^]^ Noticeably, the Tm 4d XPS spectra show that the oxidation states of the Tm species in TCN‐Cv/Tm‐Y are all located between that of the Tm^0^ and Tm^3+^ species, indicating that the atomically dispersed Tm species carry partially positive charges are well embedded in the TCN matrix, possibly forming Tm^δ+^‐N moieties.^[^ ^31^, ^32^ ^]^ In addition, the quantitative analysis results of XPS are almost consistent with those of ICP‐OES (Table S1, Supporting Information), further indicating that the Tm single‐atom sites are uniformly distributed in the porous tubular carbon nitride framework. Furthermore, Figure S10e (Supporting Information), shows the O 1s XPS spectra of the fabricated samples. The peak located at ≈532.3 eV can be attributed to surface‐adsorbed oxygen‐containing species (O_2_, H_2_O, or CO_2_), and the absence of a peak assigned to Tm‐O (530.3 eV) demonstrates the exclusion of possible Tm‐containing oxide species.^[^ ^9^, ^21^, ^33^ ^]^ In conclusion, porous tubular carbon nitride photocatalysts (TCN‐Cv/Tm‐Y) modified with Tm single atoms and C vacancies were successfully constructed in this work.

**Figure 3:**
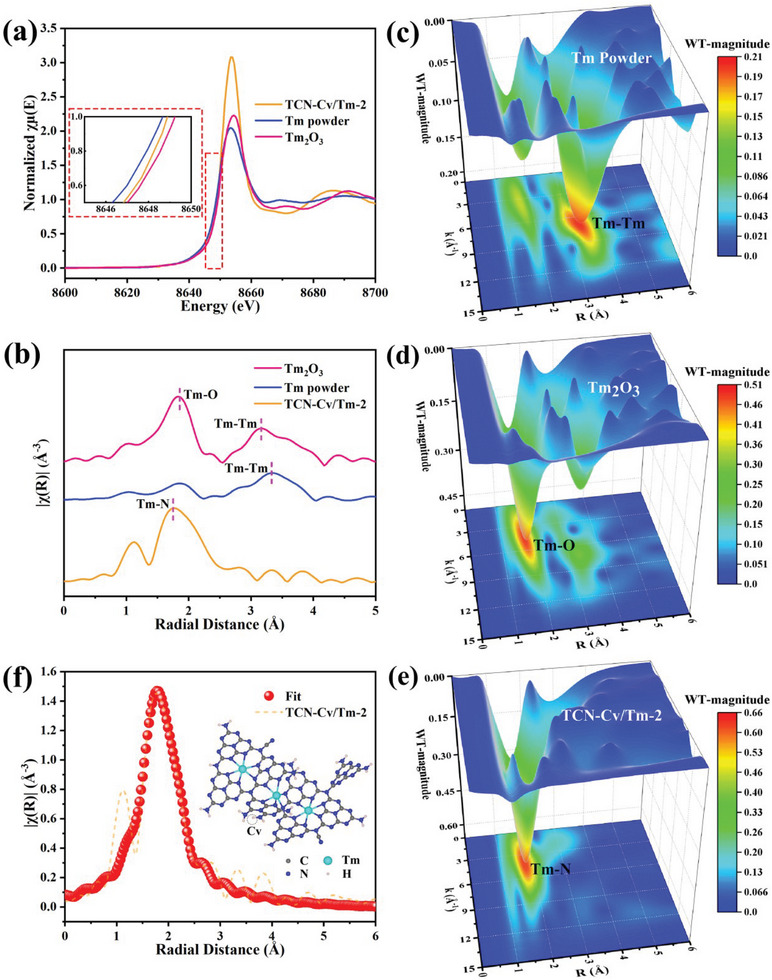
The characterization of the Tm^δ+^‐N sites. a) Normalized Tm L_3_‐edge XANES spectra of TCN‐Cv/Tm‐2 and the reference samples (inset: an enlarged image of the partial region). b) The Tm L_3_‐edge Fourier‐transformed (FT) k^2^‐weighted EXAFS spectra in the R‐space of TCN‐Cv/Tm‐2 and the reference samples. c–e) 3D WT‐EXAFS spectra of Tm powder, Tm_2_O_3_, and TCN‐Cv/Tm‐2. f) Tm L_3_‐edge EXAFS spectrum and fit curve for the TCN‐Cv/Tm‐2 sample, shown in k^2^‐weighted R‐space (inset: structure model of intra‐/interlayer Tm^δ+^‐N sites).

To further elucidate the electronic structure and local coordination environment of Tm single atoms in TCN‐Cv/Tm‐Y catalysts, its synchrotron‐based X‐ray absorption fine structure spectra (XAFS) were investigated, using the spectra of Tm powders and Tm_2_O_3_ as references. X‐ray absorption near‐edge structure (XANES) and extended X‐ray absorption fine structure (EXAFS) spectra investigations provide a more detailed understanding of the chemical valence state and coordination configuration of Tm atomic centers. As displayed in the normalized XANES spectrum of TCN‐Cv/Tm‐2, the near‐edge absorption energy of the Tm L_3_‐edge is situated between those of the Tm powders and Tm_2_O_3_ references, indicating that the Tm single atoms in TCN‐Cv/Tm‐2 are partially positively charged and the valence state is between 0 and +3 (**Figure** **3**a). Besides, the adsorption threshold energies (E_0_) of Tm in TCN‐Cv/Tm‐2, Tm powders, and Tm_2_O_3_ could be obtained by the first derivatives of Tm L_3_‐edge XANES spectra to be ≈8649.86, 8647.47, and 8650.52 eV, respectively (Figure S11a, Supporting Information). Accordingly, the average oxidation state of Tm in TCN‐Cv/Tm‐2 is +2.3, lower than that of Tm (+3) in Tm_2_O_3_, as shown in Figure S11b (Supporting Information), further confirming that the valence state of the Tm species in TCN‐Cv/Tm‐2 does indeed range from 0 to +3, which is consistent with the results of the XPS analyses. Combining the results of TEM and AC HAADF‐STEM reveals that Tm is uniformly distributed on the TCN substrate in the form of single atoms with partially positive charges. Furthermore, as displayed in the Fourier‐transformed (FT) k^2^‐weighted EXAFS spectra of the Tm L_3_‐edge in R space (Figure 3b), the dominant peak at ≈1.75 Å for TCN‐Cv/Tm‐2 could be assigned to the first‐shell coordination of the Tm─N bond, implying that the Tm atoms are anchored by the surrounding N atoms. Notably, unlike Tm_2_O_3_ and Tm powders, no coordination peak attributed to the Tm─Tm bond located at ≈3.20 Å was detected in TCN‐Cv/Tm‐2, which further confirms the absence of the formation of metallic crystalline Tm species, but rather in the form of isolated single Tm atoms existing on the TCN support, which is in accordance with the results of XRD, TEM, and AC HAADF‐STEM.^[^ ^34^ ^]^ To further reinforce this viewpoint, wavelet transform (WT) EXAFS spectral analysis was implemented as a powerful tool for high‐resolution identification of backscattering atoms in k‐space and R‐space. The WT spectrum of TCN‐Cv/Tm‐2 presents only a maximum signal intensity at ≈4.16 Å^−1^ compared to that of Tm_2_O_3_ and Tm powders, which can be attributed to the contribution of Tm─N bond (Figure 3c–e). The similar absence of contributions from the Tm─Tm bond (ca. 7.20 Å^−1^) further confirms the atomic‐level distribution of Tm sites in TCN‐Cv/Tm‐2, with the absence of metallic Tm crystalline structures. To further determine the coordination configuration of the Tm single atoms in TCN‐Cv/Tm‐2, the EXAFS spectrum was fitted and quantitatively analyzed (Figure 3f; Figure S11c and Table S5, Supporting Information). The fitting results indicate that the coordination number of Tm single atoms in TCN‐Cv/Tm‐2 is ≈6, suggesting that one Tm atom is coordinated to six N atoms. Non‐negligibly, the fitting analyses found that the Tm single atoms in TCN‐Cv/Tm‐2 exhibit the coexistence of two N‐atom coordination environments. Although the coordination numbers are both 6, the average bond lengths (R) of Tm‐N are significantly different, with R of 2.25 Å for Tm‐N_1_ and 2.41 Å for Tm‐N_2_ (Table S5, Supporting Information). This finding suggests that Tm single atoms may be involved in both in‐plane N‐coordination and interlayer N‐coordination, as reported in previous outstanding works.^[^ ^5^, ^6^, ^35^ ^]^ Based on the analysis results of XAFS, the optimized structural model of TCN‐Cv/Tm‐2 was constructed by density functional theory (DFT) calculation (inset of Figure 3f). DFT calculations confirmed the coexistence of intra‐/interlayer 6‐coordinated Tm sites (see DFT analysis section for details). The unique electronic properties and coordination configurations of the Tm single‐atom sites are expected to promote the separation and transfer of photogenerated charge carriers and enhance the adsorption and activation of CO_2_, resulting in the excellent photocatalytic CO_2_ reduction performance of TCN‐Cv/Tm‐2.

### 2.2 Evaluation of Photocatalytic CO_2_ Reduction Activity

A suitable substrate morphology structure is beneficial for the distribution of metal single atoms, the enhancement of photocatalytic performance, and the catalytic stability of the synthetic catalysts. In this work, the synthesis of porous tubular carbon nitride supports is closely related to the pressure that is applied to the precursors during the preparation process. As a result, a range of morphologically structured g‐C_3_N_4_ (TCN‐X, X = 1, 2, 3, 4, and 5 represent pressures of 48, 56, 64, 72, and 80 Mpa, respectively) was obtained by fine‐tuning the pressure that was applied to the precursors (Figures S12 and S13, Supporting Information). One can see that the porous tubular structure cannot be well‐formed without applying pressure or when the pressure is not appropriate. Then, the photocatalytic CO_2_ reduction performance of the TCN‐X samples was evaluated using pure water as a proton source (Figure S14, Supporting Information). The main product of reduction for all TCN‐X samples is CO, accompanied by trace CH_4_ output. Among them, TCN (i.e., TCN‐4) reached a CO yield of 59.96 µmol g^−1^ h^−1^ after six hours of illumination, which improved the photocatalytic performance nearly five‐fold compared with pure CN (11.91 µmol g^−1^ h^−1^) synthesized by the conventional method (Figure S14c, Supporting Information). Moreover, the photocatalytic performance of pure TCN is far ahead even when compared with the existing reported pristine carbon nitride photocatalysts (Figure S14d and Table S6, Supporting Information). The optimal support structure (TCN) was obtained based on the evaluation of the morphological structure and photocatalytic performance. The significantly improved performance of the optimized TCN sample may be mainly attributed to the porous tubular structure providing more catalytic reaction sites and gas molecule transport channels for the reduction process.

**Figure 4:**
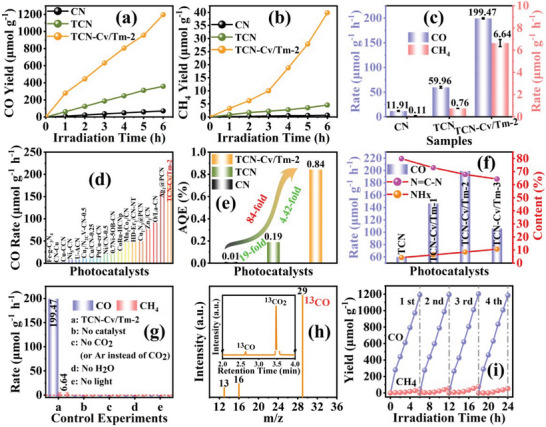
The photocatalytic CO_2_ reduction activity of CN, TCN, and TCN‐Cv/Tm‐2 samples a) The time courses of CO evolution, b) the time courses of CH_4_ evolution, and c) the average production rates of CO and CH_4_.). d) Comparison of TCN‐Cv/Tm‐2 catalyst with other carbon nitride‐based single‐atom photocatalysts. e) AQE values for CN, TCN, and TCN‐Cv/Tm‐2 samples at 385 nm. f) Relationship plot between the NHx/N = C‐N content and photocatalytic CO_2_ reduction activity over TCN and TCN‐Cv/Tm‐Y (Y = 1, 2, or 3) samples. g) Control experiments of photocatalytic CO_2_ reduction over TCN‐Cv/Tm‐2 catalyst. h) GC‐MS analysis of photocatalytic reduction products with ^13^CO_2_ as a carbon source (inset: the gas chromatography). i) Photocatalytic cyclic stability tests over TCN‐Cv/Tm‐2 catalyst for four cycles.

In further exploration, the photocatalytic CO_2_ reduction performance of the TCN‐Cv/Tm‐Y was evaluated (**Figure** **4**a,b; Figure S15a,b, Supporting Information). The yields of both CO and CH_4_ products increase monotonically with increasing irradiation duration. Meanwhile, it was found that the main product of TCN‐Cv/Tm‐Y was also CO, with no other gaseous products such as H_2_, ethylene, etc., being detected, and with a CO selectivity of more than 96.8% (Table S7, Supporting Information). Among a series of TCN‐Cv/Tm‐Y catalysts, TCN‐Cv/Tm‐2 obtained the optimal CO_2_ photocatalytic reduction performance (Figure S15c, Supporting Information). For TCN‐Cv/Tm‐2, the CO yield was as high as 199.47 µmol g^−1^ h^−1^, which is one of the best levels recently reported for carbon nitride‐based single‐atom catalysts, as well as being in the lead when compared to carbon nitride‐based catalysts in the past year (Figure 4c,d; and Tables S7 and S8, Supporting Information). Moreover, its performance and selectivity are far ahead even when compared to other state‐of‐the‐art photocatalysts that have been newly reported this year (Table S9, Supporting Information). However, there are also significant shortcomings in terms of product categories compared to other state‐of‐the‐art photocatalysts that can produce methane or C_2+_ products. Therefore, optimizing the product toward a more valuable direction than CO is worth further research in our future work. Notably, the photocatalytic performance of the optimized sample TCN‐Cv/Tm‐2 was improved by ≈16.75‐fold and 3.33‐fold, respectively, in comparison with that of bulk CN (11.91 µmol g^−1^ h^−1^) and pristine TCN (59.96 µmol g^−1^ h^−1^). Furthermore, in the oxidative half‐reaction (Figure S16, Supporting Information), the H_2_O oxidation to O_2_ was also significantly enhanced on TCN‐Cv/Tm‐2 (91.25 µmol g^−1^ h^−1^), which was ≈18.25‐fold and 3.47‐fold of that on CN (5.00 µmol g^−1^ h^−1^) and TCN (26.26 µmol g^−1^ h^−1^). The molar ratio of the generated CO to O_2_ is ≈2, confirming CO and O_2_ were the primary products in the reductive and oxidative half‐reactions, respectively. In addition, the apparent quantum efficiency (AQE), another descriptor for evaluating the catalytic efficiency of the photocatalysts, was calculated to be 0.84% for the optimal sample TCN‐Cv/Tm‐2, which is an improvement of ≈84‐fold and 4.42‐fold compared to the bulk CN (0.01%) and the pristine TCN (0.19%), respectively, indicating that this is comparable to, or even better than the AQEs of the C_3_N_4_‐based photocatalysts reported so far (Figure 4e; and Table S10, Supporting Information). Figure 4f shows the relationship between the performance of TCN‐Cv/Tm‐Y catalysts and the N═C─N and NHx contents, which symbolize the C vacancy content. It is indicated that the best performance could be achieved when the C vacancy content, i.e., the Tm single atom content, was moderate. However, with further increase in the content of Tm single‐atom sites, the photocatalytic activity decreased although the content of C vacancies still increased, which could be attributed to the slight aggregation of some Tm metal atoms or too many Tm atoms resulting in a short average interatom distance, thereby reducing the intrinsic activity of Tm single‐atom reactive sites.^[^ ^3^ ^]^ Apparently, these results indicate that the modification of moderate amounts of C vacancies and Tm single atoms sites on TCN can significantly improve the photocatalytic CO_2_ reduction performance.

Control experiments were carried out in the absence of illumination or photocatalysts or CO_2_ gas or H_2_O, and the results showed that no appreciable amounts of product were detected (Figure 4g), implying that illumination, photocatalysts, and H_2_O are essential for the photocatalytic reduction of CO_2_ to CO. Additionally, the blank experiment in which Ar was used instead of CO_2_ did not detect any products, suggesting that the generated products originated exclusively from the CO_2_ photocatalytic reduction. To further confirm this inference, an isotope labeling experiment was conducted using ^13^CO_2_ as a carbon source instead of CO_2_ under equivalent photocatalytic reaction conditions. Figure 4h exhibits a gas chromatography‐mass spectrometry (GC‐MS) graph of an isotope tracing experiment using the TCN‐Cv/Tm‐2 sample as an example. Among them, the peak at m/z = 29 in the mass spectrum can be attributed to ^13^CO, which further strongly confirms that the carbon source of the CO product is indeed derived from the CO_2_ used. In addition, the stability of the catalyst is crucial for its practical application in photocatalytic CO_2_ reduction. Therefore, the photocatalytic stability of the as‐synthesized catalysts was evaluated by cyclic reaction tests. As illustrated in Figure 4i, the yields of CO and CH_4_ on TCN‐Cv/Tm‐2 remained nearly unchanged over four runs of cyclic reactions, which affirmed its long‐lasting photocatalytic stability. This was also further supported by the XRD, SEM, TEM, SAED, AC HAADF‐STEM, XPS, and EPR structural characterization of the sample after the cyclic reactions (Figures S17 and S18, Supporting Information). No structural changes were observed from these comparative characterizations of the sample before and after the cyclic reactions, indicating that the intra‐/interlayer Tm single‐atom sites and C vacancies were well maintained during the photocatalytic reaction, and thus further confirming that the as‐synthesized catalysts have excellent photocatalytic stability.

### 2.3 In‐Depth Understanding of Photocatalytic Mechanism

**Figure 5:**
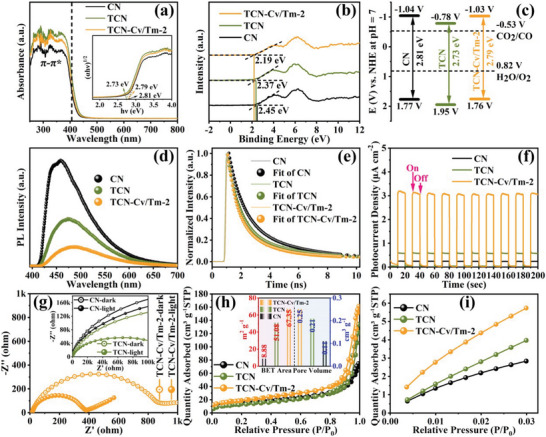
The photoelectrochemical and N_2_/CO_2_ adsorption–desorption characterizations of CN, TCN, and TCN‐Cv/Tm‐2 samples. a) UV–vis spectra (inset: Tauc‐plots of the (αhν)^1/2^ versus hν). b) VB‐XPS plots. c) Energy band position diagram. d) Steady‐state PL spectra. e) Normalized TRF emission decay spectra. f) Photocurrent density curves. g) EIS spectra with the absence and presence of light illumination. h) N_2_ adsorption–desorption isotherms at 77 K (inset: BET specific surface area and pore volume histograms). i) CO_2_ adsorption isotherms.

To investigate the optical properties and photogenerated charge carrier dynamics behavior of the as‐prepared photocatalysts, a series of photoelectrochemical measurements were carried out. As shown in **Figure** **5**a and S19a (Supporting Information), the UV–vis diffuse reflectance spectroscopy (UV‐Vis DRS) of all the samples in the range of wavelength less than 405 nm exhibit significant optical absorption, which corresponds to the intrinsic absorption of the *π*–π* electron transitions in the aromatic π‐conjugation.^[^ ^24^, ^26^ ^]^ Compared to bulk CN, the intrinsic absorption edge of TCN exhibits a significant redshift, accompanied by a decrease in the forbidden bandwidth (E_g_) from 2.81 (CN) to 2.73 eV (TCN) obtained by the Tauc plots analysis of the Kubelka‐Munk functional transformation (inset in Figure 5a; and Figure S19b, Supporting Information). This is mainly attributed to two factors, first, the generation of C≡N groups could modulate the electronic structure, leading to a narrowing of the bandgap and thus enhancing the light absorption,^[^ ^24^ ^]^ and second, owing to the formation of a porous tubular structure, which leads to multiple reflections of the incident light within the carbon nitride tubes and pores, thereby expanding the absorption of light.^[^ ^9^, ^36^ ^]^ Notably, with the introduction of Tm single atoms and C vacancies, the intrinsic absorption edge of TCN‐Cv/Tm‐Y shows a slight blueshift with respect to TCN, in which the bandgap of TCN‐Cv/Tm‐2 is 2.79 eV. On the one hand, it may be mainly due to the introduction of Tm single atoms and C vacancies adjusting the energy band structures of the synthesized catalysts, resulting in not only bandgap widening, but also a gradual broadening with the increase of the content of Tm single atoms, or C vacancies;^[^ ^14^, ^25^, ^37^ ^]^ on the other hand, the quantum confinement effect of the TCN‐Cv/Tm‐Y system may also cause the bandgap to be broadened.^[^ ^9^, ^14^, ^22^, ^25^ ^]^ In addition, the light absorption of the TCN‐Cv/Tm‐Y system gradually enhances in the range of 480–800 nm compared to that of TCN (inset in Figure S19a, Supporting Information), which may also be caused by the C vacancies.^[^ ^14^, ^37^ ^]^ In short, the introduction of Tm single atoms and C vacancies can subtly modulate the energy band structure of carbon nitride and thus improve the photocatalytic CO_2_ reduction performance, which is also confirmed by the ensuing characterizations.

Given that the redox ability of catalysts is closely related to their energy band positions, it is essential to determine the conduction band (CB) and valence band (VB) positions of the samples to further understand the suitability of the as‐prepared catalysts for photocatalytic CO_2_ reduction. Therefore, Mott‐Schottky and valence‐band XPS (VB‐XPS) tests were performed on the as‐synthesized materials. Figure S20a–e (Supporting Information) shows the Mott‐Schottky curves of the synthetic materials measured at frequencies of 2000, 2500, and 3000 Hz, respectively. One can clearly see that all the as‐prepared photocatalysts exhibit a positive slope, indicating that all of them possess n‐type semiconductor characteristics.^[^ ^38^ ^]^ The flat‐band potentials (E_fb_) of Bulk CN, TCN, TCN‐Cv/Tm‐1, TCN‐Cv/Tm‐2, and TCN‐Cv/Tm‐3 are determined by the intersection of the Mott‐Schottky plots with the horizontal axis and are −0.90, −0.64, −0.60, −0.65, and −0.70 V versus Ag/AgCl (pH = 7) (Figure S20a–e, Supporting Information). E_fb_ (vs normal hydrogen electrode (NHE) at pH = 7) can be calculated according to the following equation: E = E_Ag/AgCl_ – E^θ^_Ag/AgCl_ + 0.059 * pH, (vs NHE, pH = 7), where E^θ^_Ag/AgCl_ (pH = 7) is 0.197 V.^[^ ^39^ ^]^ Accordingly, the E_fb_ (vs NHE at pH = 7) of CN, TCN, TCN‐Cv/Tm‐1, TCN‐Cv/Tm‐2, and TCN‐Cv/Tm‐3 can be determined to be −0.684, −0.424, −0.384, −0.434, and −0.484 V, respectively. Besides, the VB‐XPS spectra of all synthetic catalysts are displayed in Figure 5b and Figure S20f (Supporting Information). It can be seen that the energy gaps between Fermi level (E_f_) and VB are 2.45 (CN), 2.37 (TCN), 2.30 (TCN‐Cv/Tm‐1), 2.19 (TCN‐Cv/Tm‐2), and 2.12 eV (TCN‐Cv/Tm‐3), respectively. For n‐type semiconductors, the flat‐band potential is equal to the Fermi level, and thus their VB positions can be calculated to be 1.77 (CN), 1.95 (TCN), 1.92 (TCN‐Cv/Tm‐1), 1.76 (TCN‐Cv/Tm‐2), and 1.64 eV (TCN‐Cv/Tm‐3).^[^ ^40^ ^]^ The upward shift in the VB potential of the TCN‐Cv/Tm‐Y system compared to that of the TCN may be due to the broadening of the valence band induced by the C vacancy.^[^ ^22a^ ^]^ Combining the forbidden bandwidths previously analyzed, it can be concluded that their CB positions are −1.04 (CN), −0.78 (TCN), −0.85 (TCN‐Cv/Tm‐1), −1.03 (TCN‐Cv/Tm‐2), and −1.15 eV (TCN‐Cv/Tm‐3), respectively. The above results further demonstrate that the modification of Tm single atoms and C vacancies modulated the energy band structure of TCN, as shown in Figure 5c and Figure S21 (Supporting Information). Obviously, the CB potentials of all catalysts are more negative than that required for CO production (CO_2_/CO: −0.53 V, vs NHE at pH = 7), which means that the prepared photocatalysts are all thermodynamically capable of satisfying the photocatalytic reduction of CO_2_ to CO. Moreover, the CB potential of the TCN‐Cv/Tm‐Y system presents an obvious upshift compared with that of the TCN, indicating that the photogenerated electrons of the TCN‐Cv/Tm‐Y system have a stronger reduction ability, resulting in the TCN‐Cv/Tm‐Y exhibiting a higher CO_2_ reduction efficiency. In addition, the slope of the Mott‐Schottky curve is inversely proportional to the concentration of carriers.^[^ ^6^, ^41^ ^]^ As shown in Figure S22 (Supporting Information), the slopes of the TCN‐Cv/Tm‐Y system are all significantly smaller than that of the TCN, and it is noteworthy that the slope of the TCN‐Cv/Tm‐2 is the smallest, which indicates that the TCN‐Cv/Tm‐2 has a higher carrier density than that of TCN‐Cv/Tm‐1, TCN‐Cv/Tm‐3, and TCN, thus leading TCN‐Cv/Tm‐2 to exhibit the highest photocatalytic CO_2_ reduction performance. This finding may predict that the introduction of atomically dispersed intra‐/interlayer Tm centers and C vacancies improves the separation efficiency of photogenerated electron‐hole pairs.

To evaluate the effect of the introduction of Tm single atoms and C vacancies on the separation and transfer of photogenerated charge carriers in the synthetic catalysts, the steady‐state photoluminescence (PL) spectra and time‐resolved fluorescence (TRF) emission decay spectra were measured. As shown in Figure 5d and Figure S23a (Supporting Information), the bulk CN shows the highest PL intensity, while the intensity of TCN is much weaker relative to CN, indicating that the porous tubular structure favors the separation and transfer of photogenerated charge carriers. More importantly, the PL intensity of the TCN‐Cv/Tm‐Y system exhibits further attenuation, demonstrating that the introduction of Tm single atoms and C vacancies significantly inhibits the recombination of photoinduced charge carriers. Also, for better comparison, the photoexcited charge‐carrier dynamics of all fabricated catalysts were investigated by TRF spectra (Figure 5e; Figure S23b–f and Table S11, Supporting Information). Notably, a bi‐exponential fitting to those TRF spectra reveals that the longer lifetime (τ_2_, reflecting non‐radiative recombination) decays faster than the shorter lifetime (τ_1_, reflecting radiative fluorescence quenching) with the formation of porous tubular structures as well as the introduction of Tm species and C vacancies, and that the estimated average fluorescence lifetimes (τ_ave_) are 3.35 (bulk CN), 2.75 (TCN), 2.03 (TCN‐Cv/Tm‐1), 1.73 (TCN‐Cv/Tm‐2), and 2.22 ns (TCN‐Cv/Tm‐3), which implies a more unimpeded photogenerated charge‐carrier separation and transfer over these porous tubular catalysts TCN‐Cv/Tm‐Y co‐modified by single‐atom Tm species and C vacancies.^[^ ^39^, ^42^ ^]^ Additionally, based on the following equation: K_et(TCN→TCN‐Cv/Tm‐Y)_ = 1/τ_ave(TCN‐Cv/Tm‐Y)_ – 1/τ_ave(TCN)_, the electron transfer rate constants (K_et_) for the TCN‐Cv/Tm‐Y system can be calculated as 1.2897*10^8^ (TCN‐Cv/Tm‐1), 2.1440*10^8^ (TCN‐Cv/Tm‐2), and 0.8681*10^8^ s^−1^ (TCN‐Cv/Tm‐3), which further reveals that the introduction of Tm single atoms and C vacancies effectively promotes the transfer of photoexcited electrons from TCN support to the reaction sites.^[^ ^3^, ^14^, ^42^ ^]^ Furthermore, according to the above analyses, TCN‐Cv/Tm‐2 presents the lowest PL intensity, the smallest τ_ave_, and the largest K_et_, indicating that it has the best‐photogenerated charge carrier separation efficiency, which is in line with its exhibiting the optimal photocatalytic CO_2_ reduction performance. In addition, the transient photocurrent response (TPR) and electrochemical impedance spectroscopy (EIS) measurements were also performed over the as‐prepared photocatalysts. As shown in Figure 5f,g and Figure S24a,b (Supporting Information), the TCN‐Cv/Tm‐Y system exhibits higher photocurrent density and smaller arc radius of electrochemical impedance than those of the pristine TCN and bulk CN, suggesting that there is a smaller charge transfer resistance in the TCN‐Cv/Tm‐Y system, thereby enhancing the rapid transfer and separation of photoexcited electrons, which is in agreement with the analyses of PL and TRF. Besides, the arc radius of the EIS is smaller in light than in darkness, implying that illumination further improves the photogenerated charge separation and transfer process. Consequently, these photoelectrochemical analysis results provide strong evidence that the existence of Tm single‐atom sites and C vacancies could significantly enhance the separation and transfer of photogenerated electron‐hole pairs, and thus confer superior CO_2_ photocatalytic reduction performance to the TCN‐Cv/Tm‐Y system. Among them, TCN‐Cv/Tm‐2 exhibits the most efficient photoinduced carrier separation and transfer, which resulted in TCN‐Cv/Tm‐2 presenting the highest photocatalytic CO_2_ reduction efficiency.

Adsorption of reactants plays an important role in the catalytic performance of photocatalysts. In general, the specific surface area of a catalyst is positively correlated with its adsorption capacity for reactants. The specific surface area and pore structure distribution of all as‐synthesized catalysts were analyzed by Brunauer‐Emmett‐Teller (BET) tests (Figure 5h; Figure S25 and Table S12, Supporting Information). It can be seen that the specific surface area and pore volume of TCN are approximately six‐fold and two‐fold higher than that of bulk CN, respectively, which is attributed to the different geometric structures. Moreover, with the introduction of Tm single atoms and C vacancies, the BET‐specific surface area showed a further increase, in which the specific surface area of TCN‐Cv/Tm‐2 was as high as 67.35 m^2^ g^−1^, suggesting that the formation of porous tubular structure as well as modification of an appropriate amount of Tm species and C vacancies could contribute to a larger specific surface area.^[^ ^14^, ^39^, ^43^ ^]^ Furthermore, detailed pore size analyses (Figure S25 and Table S12, Supporting Information) indicate that the as‐prepared photocatalysts mainly exhibit mesoporous distribution accompanied by a small fraction of macropores with 50–150 nm pore sizes. Moreover, there were no obvious alterations in the pore structure of the TCN‐Cv/Tm‐Y system, indicating that the introduction of Tm single atoms and C vacancies did not disrupt the porous tubular structure of TCN, which is consistent with the results of the morphological analysis of the samples. The enlarged specific surface area and abundant porous tubular structure are beneficial for increasing the adsorption of reactants or exposing more reaction sites, resulting in the excellent photocatalytic performance of the TCN‐Cv/Tm‐Y system. To further support this viewpoint, CO_2_ adsorption capacity measurements were performed on all as‐synthesized catalysts. Figure 5i and Figure S26 (Supporting Information), display the CO_2_ adsorption isotherms for all samples at 273.15 K. It can be seen that the adsorption amounts for all samples are 2.838 (bulk CN), 3.968 (TCN), 4.643 (TCN‐Cv/Tm‐1), 5.735 (TCN‐Cv/Tm‐2), and 4.880 cm^3^ g^−1^ (TCN‐Cv/Tm‐3), respectively, at a relative pressure of 0.03, indicating that the generation of porous tubular structures together with the introduction of single‐atom Tm species and C vacancies enhanced CO_2_ adsorption, which is consistent with the results of BET specific surface area analyses.^[^ ^6^, ^14^, ^43^ ^]^ Notably, TCN‐Cv/Tm‐2 exhibits the strongest CO_2_ adsorption capacity, which is roughly 2.02 and 1.45 fold higher than that of bulk CN and TCN, respectively, implying that a higher apparent reactant concentration exists over TCN‐Cv/Tm‐2, leading to a higher CO_2_ reduction activity. Therefore, all these results also confirm that efficient CO_2_ reduction photocatalysts could be obtained by co‐modifying porous tubular TCN with Tm single atoms and C vacancies.

The effective adsorption and activation of CO_2_ molecules on the catalyst surface are crucial for the photocatalytic CO_2_ reduction process. In situ Fourier transform infrared spectroscopy (ISFTIR) characterization is a powerful means for dynamic monitoring and identifying surface‐bound species on catalysts in the field of photocatalytic CO_2_ reduction. In the meantime, in order to further investigate the role of Tm single atoms and C vacancies in the photocatalytic CO_2_ reduction reaction, therefore, the as‐prepared photocatalysts were subjected to ISFTIR spectroscopy tests. Figure S27 (Supporting Information) illustrates the time‐resolved ISFTIR spectra of the catalysts TCN‐Cv/Tm‐2, TCN, and CN after the introduction of a moist CO_2_ atmosphere under dark conditions. It can be seen that the characteristic IR peaks of adsorbed species and CO_2_‐derived intermediates on the catalyst surface, such as c‐CO_3_^2−^ (ν(C‐O): ≈1727 and ≈1755 cm^−1^), ·CO_2_^−^ (symmetric CO_2_ stretching vibration (ν_s_(O‐C‐O)): ≈1706 and 1660–1690 cm^−1^), b‐CO_3_^2−^ (ν_s_(CO_3_): 1295–1315, 1360–1371, and 1401 cm^−1^, asymmetric CO_3_ stretching vibration (ν_as_(CO_3_)): ≈1552, ≈1572, and ≈1599 cm^−1^), m‐CO_3_^2−^ (ν_s_(CO_3_): ≈1280 and 1450–1520 cm^−1^), and bicarbonate HCO_3_^−^ (σ(CHO): 1245–1270, ≈1340, and 1420–1440 cm^−1^) (Figure S27a–c, Supporting Information).^[^ ^42^, ^44^ ^]^ All of these observed species are important intermediates involved in the CO_2_ photocatalytic conversion, and originating from the surface‐bound interaction between adsorbed CO_2_ and the catalyst. The absorption peaks located at ≈1625 and ≈1645 as well as ≈2057 and ≈2076 cm^−1^ could be attributed to the bending vibration and combination band of H_2_O, respectively (Figure S27a–f, Supporting Information).^[^ ^44^, ^45^ ^]^ Figure S27g–l (Supporting Information) presents the characteristic vibrational peaks of CO_2_ (ν_3_(CO_2_)) situated at 2317–2371 and 3597–3725 cm^−1^.^[^ ^42^, ^46^ ^]^ It can be noticed that the intensity of the characteristic vibrational peaks of all these species is enhanced with extended adsorption time (Figure S27, Supporting Information). Moreover, meticulous observation reveals that the order of the vibrational peak intensities of these species is TCN‐Cv/Tm‐2 > TCN > CN, which is consistent with the activity trend of photocatalytic CO_2_ reduction, where the intensity implies the adsorption and activation capacity of different catalysts for CO_2_ under the equivalent conditions.^[^ ^21^, ^42^ ^]^ It is also noteworthy that CO_2_ exists predominantly as chelating‐bridged carbonates (c‐CO_3_^2−^) in bulk CN, whereas it exists primarily as ·CO_2_^−^ species in porous tubular TCN and TCN‐Cv/Tm‐2, which is considered to be a key evidence for CO_2_ activation. Thus, these results indicate that TCN and TCN‐Cv/Tm‐2 could adsorb and activate CO_2_ molecules more efficiently than CN.^[^ ^44b^ ^]^ In addition, the IR absorption peak intensities of CO_2_ and CO_2_‐derived species on TCN‐Cv/Tm‐2 are also remarkably higher than those of the corresponding peaks on TCN, especially the peak intensity contrast of the ·CO_2_^−^ species is even more significant, which is in good agreement with the results of the CO_2_ adsorption isotherms analyzed in Figure 5i, which may be mainly attributed to the presence of the single‐atom Tm sites and C vacancies promoting the CO_2_ adsorption and activation. Significantly enhanced adsorption and activation of CO_2_ molecules are key to the excellent photocatalytic reduction reaction performed by TCN‐Cv/Tm‐2.

**Figure 6:**
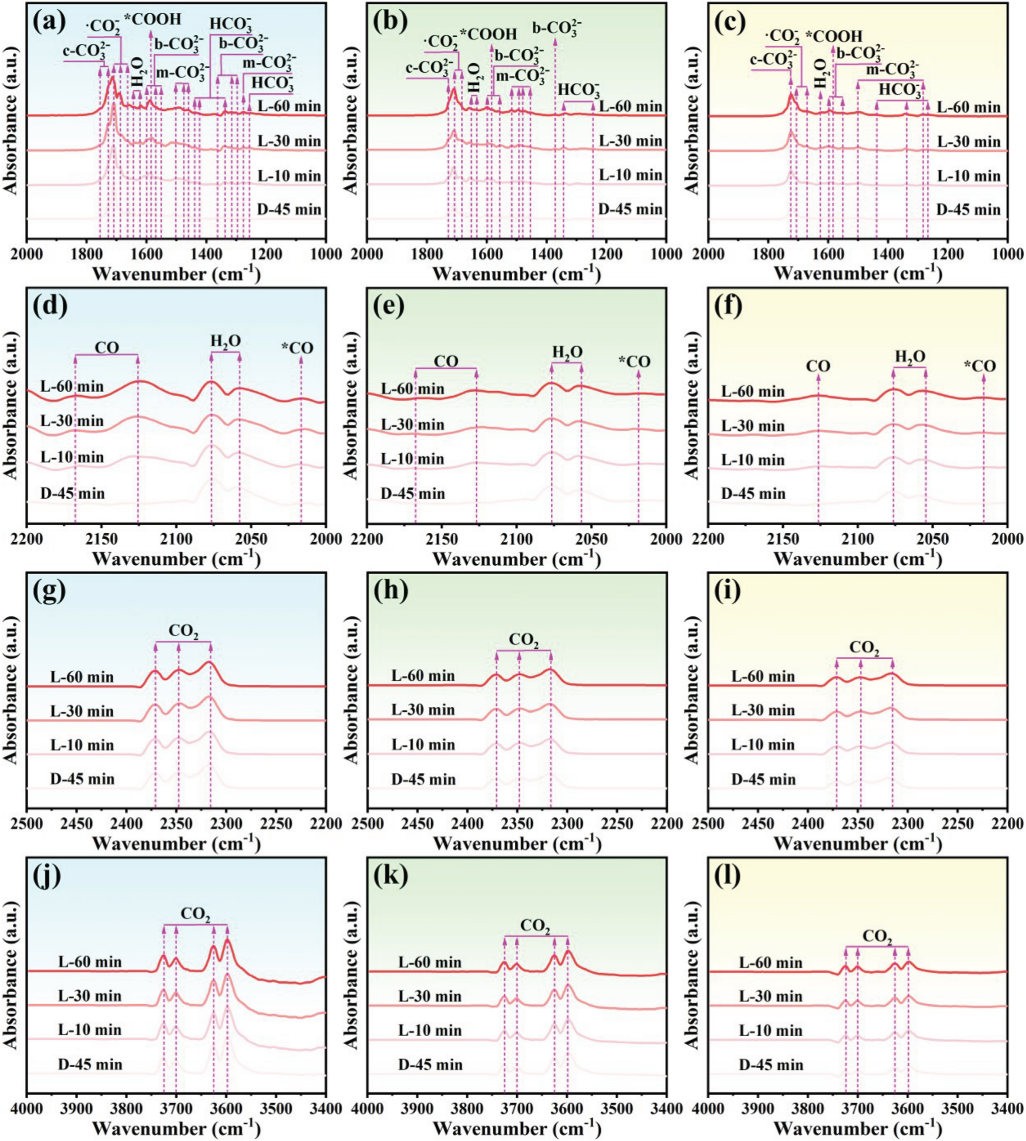
The ISFTIR spectra of CO_2_ and H_2_O interaction with a,d,g,j) TCN‐Cv/Tm‐2, b,e,h,k) TCN, or c,f,i,l) CN under subsequent light irradiation (D refers to dark environment, L refers to light environment).

Upon the photo‐irradiation, the absorption peak intensities of the CO_2_ derivative species and CO_2_ molecules continued to climb with the prolongation of reaction time, suggesting that the adsorption and conversion of the CO_2_ were proceeding uninterruptedly (**Figure** **6**). Moreover, the absorption peak intensity of TCN‐Cv/Tm‐2 is appreciably higher than that of TCN and CN, indicating that the porous tubular carbon nitride co‐decorated with Tm single atoms and C vacancies significantly enhances the adsorption and conversion of reactants. Notably, a new vibrational peak located at ≈1585 cm^−1^ appears in the spectra under illumination, and the intensity of the peak continues to increase over time (Figure 6a–c). This peak belongs to the characteristic vibrational peak of the *COOH intermediate, which is regarded as the crucial intermediate in the photocatalytic reduction of CO_2_ to CO or CH_4_.^[^ ^21^, ^45^ ^]^ Meanwhile, the characteristic absorption peaks of the *CO (CO gas) species can be clearly seen in Figure 6d–f (≈2016 for *CO; ≈2126 and ≈2166 for CO gas), and the peak intensities are likewise observed to continuously increase throughout the entire testing process.^[^ ^45^, ^47^ ^]^ Most importantly, the absorption peak intensity of these species still exhibits the following order: TCN‐Cv/Tm‐2 > TCN > CN, indicating that TCN‐Cv/Tm‐2 possesses the highest keystone species generation efficiency, which is well in agreement with its showing optimal CO_2_ photoreduction performance. These results further demonstrate that the dual strategy of constructing a porous tubular structure and introducing both Tm single‐atom sites and C vacancies could greatly improve the adsorption and activation of reactants on carbon nitride, thereby achieving outstanding CO_2_ reduction photocatalysts.

To further elucidate in depth the intrinsic mechanism of the significant enhancement of photocatalytic CO_2_ reduction activity, density functional theory (DFT) computational simulations were carried out. According to the Tm coordination environment determined by X‐ray absorption near‐edge structure (XANES) and extended X‐ray absorption fine structure (EXAFS) spectroscopy at the Tm L_3_‐edge, a 3 × 3 supercell of one‐layered C_3_N_4_ was used to construct the Tm‐doped C_3_N_4_ with C vacancy and ‐CN group. Along with the presence of C vacancy, ‐NH_2_ groups are obviously increased in as‐prepared materials. Thus, five material models were first simulated for comparison, namely CN, TCN, TCN/Tm, TCN‐Cv/Tm, and TCN‐Cv/2Tm. In experiments, it was observed that the introduction of Tm or rather the introduction of both Tm and C vacancy can significantly enhance the activity of CO_2_ reduction to CO. Herein, the calculated free energy diagrams to CO_2_ reduction through CO_2_ to CO reaction path are presented in Figure S28 (Supporting Information). For the CO_2_ reduction reaction, the initial reduction through a proton‐electron transfer step forms an O─H bond and thereby producing a *COOH intermediate. The ∆G for CO_2_ hydrogenation to *COOH is uphill by 2.29 eV on the bare CN, and it is decreased by 0.55 eV with the modification of the ─CN group (Figure S28a, Supporting Information). This result confirms that TCN has superior photocatalytic activity than pure CN and that the reaction site may be located at s1. However, for both bare CN and pristine TCN structures, the reaction barrier is relatively huge in the whole reaction path. When introducing the Tm site, the ∆G for CO_2_ hydrogenation to *COOH is downhill though downhill trend is not much distinct except that the TCN‐Cv/Tm‐s2 site (reaction site is not located at Tm, Figure S28b, Supporting Information). Consequently, Tm doping can significantly decrease the energy barrier for the *COOH step and therefore promote CO_2_ reduction. Notably, the existence of C vacancy coupling with the Tm introduction is likely to further enhance photoreduction performance, while the origin of activity enhancement could be ascribed to a better CO_2_ activation. Looking at Figure S28b (Supporting Information), both the TCN‐Cv/Tm‐s1 (−0.74 eV) and TCN‐Cv/2Tm‐s1 (−0.76 eV) show a lower CO_2_ activation energy barrier than the TCN/Tm‐s1 (−0.49 eV), indicating that the presence of C vacancy promotes the adsorption and activation of CO_2_ at intralayer Tm sites. To test the impact of C vacancy close to the Tm site, TCN‐Cv/2Tm‐s2 was calculated in the reaction path, where the CO_2_ activation energy barrier is further decreased to −1.06 eV (Figure S28b, Supporting Information). This further suggests that the C vacancy could enhance the activation ability of Tm sites for CO_2_, especially for Tm sites closer to the C vacancy. Based on the fact that the ‐NH_2_ site induced by the C vacancy is unfavorable for the reductive conversion of CO_2_ and that the Tm site serves as a reaction site for CO_2_, we hypothesize that the ‐NH_2_ site may be an oxidation reaction site for H_2_O. Therefore, we calculated the oxidation reaction pathway of H_2_O at the ‐NH_2_ site (Figure S29, Supporting Information). The results showed that the dissociation energy barrier of H_2_O on the ‐NH_2_ site near the C vacancy was significantly reduced, indicating that ‐NH_2_ is indeed the active site for O_2_ evolution. In addition, the activity of photocatalytic CO_2_ reduction can also be reflected by the adsorption ability of reaction sites for CO_2_ and *COOH intermediates.^[^ ^48^ ^]^ DFT calculations showed that TCN has a stronger adsorption ability than pure CN, especially at the s1 site (Table S13, Supporting Information). Compared with pure TCN, the introduction of Tm single atoms and C vacancies dramatically increased the adsorption energy of CO_2_ and *COOH intermediates at each reaction site, except for the s2 site (the reaction site is not located at Tm). This suggests that the introduction of single‐atom Tm sites significantly enhances CO_2_ adsorption and conversion and that the presence of C vacancies strengthens this process. Furthermore, the Tm site adjacent to the C vacancy possesses a stronger adsorption ability. Briefly, the adsorption ability of the reaction site for CO_2_ and *COOH intermediates is consistent with the trend of CO_2_ activation on these structures. Strong CO_2_ and *COOH intermediate adsorption are the prerequisite for CO_2_ conversion, accordingly, it can also be inferred that the order of photocatalytic activity is TCN‐Cv/Tm‐Y > TCN > CN, thus confirming the findings of the photocatalytic tests.^[^ ^48^ ^]^

**Figure 7:**
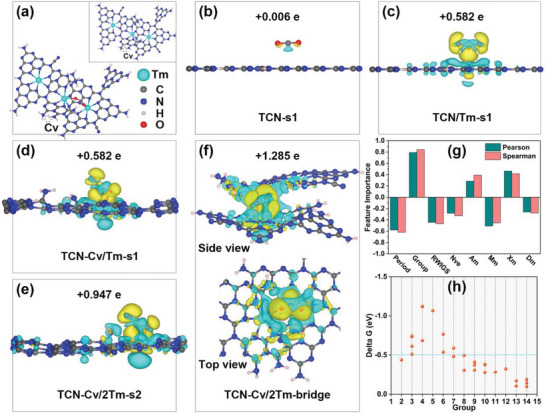
a) The optimized TCN‐Cv/2Tm‐bridge (TCN‐Cv/Tm‐2) model with adsorbed *CO_2_ (inset: the optimized model without adsorbed *CO_2_). b–f) Side view or Top view of the charge density difference for *CO_2_ adsorption. Charge depletion and accumulation are presented in cyan and yellow, respectively. Isosurface = 0.001 e Å^−3^. g) Importance of using 8 features through Pearson and Spearman methods. h) Correlation between energy barrier of CO_2_ activation reaction and metal element descriptor‐Group.

Regarding that Tm atom in the as‐prepared material is six‐coordinated, another material model TCN‐Cv/2Tm‐bridge, which is probably closest to the real optimal sample (TCN‐Cv/Tm‐2) structure, was optimized and the CO_2_ activation energy barrier was calculated as well (**Figure** **7**a). By X‐ray absorption fine structure (XAFS) analysis, two types of Tm coordination present the different Tm─N bond lengths, with an average of 2.25 and 2.41 Å in experiment results. The optimized TCN‐Cv/Tm and TCN‐Cv/2Tm‐bridge were used to observe the Tm coordination environment, in which 2.39 Å of average Tm─N bond length was calculated for the TCN‐Cv/Tm, and 2.41 Å (intralayer) and 2.35 Å (interlayer) of average Tm─N bond length were calculated for the TCN‐Cv/2Tm‐bridge structures, respectively. It indicates that Tm coordination observed in experiments is located in the intralayer and interlayer. The CO_2_ activation energy barrier around the interlayer area is −2.40 eV, much lower than that in the TCN‐Cv/2Tm site, demonstrating that the interlayer Tm site with adjacent C vacancy could accelerate the formation of activated *CO_2_ species more than intralayer Tm site and thereby facilitating CO_2_ photoreduction to CO. In addition, the activation ability of CO_2_ can be reflected by the extent of charge transfer between the reactive site and the adsorbed CO_2_ molecule. As shown in Figure 7b–f, it is clearly seen that more charges can be transferred to the adsorbed CO_2_ molecule (0.582 e) from the Tm active center compared to TCN‐s1 (0.006 e), suggesting that the Tm site could effectively facilitate the CO_2_ adsorption and the following *COOH formation. When Tm is adjacent to C vacancy coupled with resulting ‐NH_2_, the active site (TCN‐Cv/2Tm‐s2) can strongly transfer charge to *CO_2_ (0.947 e). It is shown that the presence of the C vacancy could enhance the activation of CO_2_ by the neighboring Tm site and the subsequent generation of *COOH intermediate. When Tm was located at the interlayer (TCN‐Cv/2Tm‐bridge), benefiting from the formation of interlayer Tm‐N charge transfer channels, thereby leading to the charge transfer toward adsorbed *CO_2_ was further significantly increased (1.285 e), suggesting that the interlayer Tm site with neighboring C vacancy has stronger CO_2_ adsorption and activation ability than the intralayer Tm site. In brief, the charge transfer degree is consistent with the trend of CO_2_ activation on these structures. Furthermore, the Tm sites can also be identified as centers for CO_2_ adsorption and activation based on the findings of the hydrophobic/hydrophilic tests (please refer to the contact angle (CA), which is a fundamental parameter to analyze the wettability of a triple‐phase interface, presented in Figure S30 (Supporting Information, and the additional discussions).^[^ ^49^ ^]^ Therefore, combining the above analysis and discussion, the proposed photocatalytic CO_2_ reduction mechanism is illustrated in Figure S31 (Supporting Information). The synergistic effect of C vacancies and intra‐/interlayer rare‐earth Tm single atoms significantly enhanced the separation/transfer of photogenerated electrons and promoted CO_2_ adsorption/activation. The interlayer‐bridged Tm─N bonds served as electron transfer channels, resulting in stronger electron capture ability for interlayer Tm sites than for intralayer ones. Meanwhile, C vacancies further enhanced the CO_2_ adsorption/activation at the single‐atom Tm sites. The comprehensive effect leads to an optimized reaction path and ultimately achieves a superior CO_2_ photo‐reduction activity.

Based on the above DFT results and comparison of CO_2_ photoreduction activity on different metal‐doped C_3_N_4_, it is indicated that different metal‐doped C_3_N_4_ modified with ‐CN group and C vacancy could also have different performance on this reaction, especially on CO_2_ activation step. Consequently, 28 activation energy barriers (Delta G) of different metal active sites (except Tm) were obtained by DFT calculations and used for correlation analysis between the used descriptors and Delta G through the Pearson correlation coefficient (Pearson) and the Spearman correlation coefficient (Spearman) with the help of machine learning (ML). The descriptors and corresponding Delta G are presented in Table S14 (Supporting Information). As shown in Figure 7g, the rankings of these descriptors by Pearson are basically consistent with those by Spearman. Notably, descriptors such as Group, Am, and Xm show a positive correlation coefficient, while others such as Period, RWIGS, Nve, Mm, and Dm display a negative correlation coefficient. Among these descriptors, Group shows the biggest correlation coefficient, therefore the map of Group descriptor and Delta G was plotted. It can be seen that 3, 4, 5, and 6 of Group metals present more possibility to better activate CO_2_ adsorption on metal‐C_3_N_4_‐CN‐Cv structures (Figure 7h), which gives a potential clue to explore better catalysts for CO_2_ photoreduction in experiments.

## 3 Conclusion

In summary, in‐plane/interlayer single‐atom Tm modified porous tubular carbon nitride containing C vacancy photocatalyst for CO_2_ photo‐reduction has been successfully designed and synthesized. Benefiting from the design of C vacancy‐containing porous tubular structure with intra‐/interlayer active Tm sites, TCN‐Cv/Tm‐2 exhibited a highly selective (96.8%) CO production (199.47 µmol g^−1^ h^−1^) and excellent stability, which was superior to most of the reported carbon‐nitride‐based photocatalysts. Results showed that the synergistic effect of C vacancies and intra‐/interlayer rare‐earth Tm single atoms significantly enhanced the utilization efficiency of photogenerated electrons, promoted CO_2_ adsorption/activation, reduced *COOH formation energy, optimized the reaction path, and ultimately achieved a superior CO_2_ photo‐reduction activity. Among them, the interlayer‐bridged Tm─N bonds served as electron transfer channels, resulting in stronger CO_2_ activation ability for interlayer Tm sites than for intralayer ones. Meanwhile, C vacancies further enhanced the CO_2_ adsorption/activation at the single‐atom Tm sites. This study may provide a strategy for designing efficient single‐atom CO_2_ reduction photocatalysts and a mechanistic insight into the enhancement of photocatalytic performance using rare‐earth element modifications.

## Conflict of Interest

The authors declare no conflict of interest.

## Supporting information

Supporting Information

## Data Availability

The data that support the findings of this study are available from the corresponding author upon reasonable request.
