# Supplementary material for: Outstanding CO2 Photoreduction in Single‐Atom Thulium Modified Carbon Nitride
Source: Adv Sci (Weinh). 2024 Aug 9;11(38):2406329. doi: 10.1002/advs.202406329 (PMC11481386; doi:10.1002/advs.202406329)
Supplement: Supplementary file 1 — Supporting Information [file ADVS-11-2406329-s001.docx]

Supporting Information

**Outstanding CO_2_ Photoreduction in Single-atom Thulium Modified Carbon Nitride**

*Cheng Ding, Liuqing Yang,* Xinxin Lu, Haoqiang Chi, Yong Yang, Junyang Yuan, Xiaoyong Wang, Xinglong Wu, Yongcai Zhang, Yong Zhou,* and Zhigang Zou*

C. Ding, H. Chi, J. Yuan, X. Wang, X. Wu, Y. Zhou, Z. Zou

Key Laboratory of Modern Acoustics (MOE), Institute of Acoustics, School of Physics, National Laboratory of Solid-State Microstructures, College of Engineering and Applied Sciences, Collaborative Innovation Center of Advanced Microstructures, Eco-Materials and Renewable Energy Research Center (ERERC), Jiangsu Key Laboratory for Nano Technology, Nanjing University, Nanjing, Jiangsu, 210093, P. R. China.
E-mail: zhouyong1999@nju.edu.cn

L. Yang
College of Science, Nanjing Forestry University, Nanjing, Jiangsu, 210037, P. R. China.

E-mail: liuqingyang@njfu.edu.cn

L. Yang
Kunshan Sunlaite New Energy Co. Ltd., Kunshan Innovation Institute of Nanjing University, No. 1666, South Zuchongzhi Road, Kunshan, Jiangsu 215347, P. R. China.

E-mail: liuqingyang@njfu.edu.cn

X. Lu

PetroChina Shenzhen New Energy Research Institute, Shenzhen, Guangdong, 518052, P. R. China.

Y. Yang

Key Laboratory of Soft Chemistry and Functional Materials (MOE), Nanjing University of Science and Technology, Nanjing, Jiangsu, 210094, P. R. China.

Y. Zhang

School of Chemistry and Chemical Engineering, Yangzhou University, Yangzhou 225009, P. R. China.

Y. Zhou, Z. Zou

School of Science and Engineering, The Chinese University of Hongkong (Shenzhen), Shenzhen, Guangdong, 518172, P. R. China.

E-mail: zhouyong1999@nju.edu.cn

Y. Zhou

School of Chemical and Environmental Engineering, Anhui Polytechnic University, Wuhu, Anhui, 241000, P. R. China.

E-mail: zhouyong1999@nju.edu.cn

**Catalogue**

[1. Experimental section 4](#_Toc171678909)

[1.1 Chemicals and reagents 4](#_Toc171678910)

[1.2 Catalysts synthesis 4](#_Toc171678911)

[1.3 Materials characterization 5](#_Toc171678912)

[1.4 Evaluation of CO_2_ photo-reduction performance 6](#_Toc171678913)

[1.5 Photoelectrochemical measurements 8](#_Toc171678914)

[1.6 Density functional theory (DFT) calculations 8](#_Toc171678915)

[2. Supplementary data 9](#_Toc171678916)

[2.1 Schematic diagram for synthesis 9](#_Toc171678917)

[2.2 SEM (EDS), TEM (SAED), and XPS analysis of TCN 10](#_Toc171678918)

[2.3 XRD, SEM, TEM (SAED), and XPS analysis of CN 11](#_Toc171678919)

[2.4 Table of Tm content analysis 12](#_Toc171678920)

[2.5 XRD, SEM, and TEM characterization of TCN-Cv/Tm-Y (Y = 1, 2, or 3) 12](#_Toc171678921)

[2.6 AC HAADF-STEM images of TCN-Cv/Tm-2 13](#_Toc171678922)

[2.7 FTIR and EPR spectra of CN, TCN, and TCN-Cv/Tm-Y 14](#_Toc171678923)

[2.8 XPS analysis of TCN-Cv/Tm-Y 15](#_Toc171678924)

[2.9 Organic elemental analysis (OEA) 17](#_Toc171678925)

[2.10 Synchrotron radiation analysis 18](#_Toc171678926)

[2.11 XRD and SEM characterization of TCN-X (X = 1, 2, 3, 4, and 5) 20](#_Toc171678927)

[2.12 Photocatalytic CO_2_ reduction activity 21](#_Toc171678928)

[2.13 Tables of performance comparison data 23](#_Toc171678929)

[2.14 O_2_ evolution rate during CO_2_ reduction 29](#_Toc171678930)

[2.15 Table of AQE comparison data 29](#_Toc171678931)

[2.16 Characterizations of TCN-Cv/Tm-2 after the cyclic stability test 30](#_Toc171678932)

[2.17 Band structure analysis 31](#_Toc171678933)

[2.18 Photoelectrochemical analysis 33](#_Toc171678934)

[2.19 Analysis of specific surface area and pore structure 35](#_Toc171678935)

[2.20 CO_2_ adsorption isotherms analysis 36](#_Toc171678936)

[2.21 ISFTIR analysis 37](#_Toc171678937)

[2.22 Calculation simulation analysis 38](#_Toc171678938)

[2.23 Hydrophilicity/hydrophobicity analysis 41](#_Toc171678939)

[2.24 Table of machine learning (ML) parameters 42](#_Toc171678940)

[2.25 Schematic diagram of reaction mechanism 43](#_Toc171678941)

[References 44](#_Toc171678942)

# 1. Experimental section

## 1.1. Chemicals and reagents

Urea (CH_4_N_2_O, ≥ 99.5%, Aladdin), melamine (C_3_H_6_N_6_, ≥ 99.0%, Sinopharm-SCR), thulium chloride hexahydrate (TmCl_3_·6H_2_O, 99.99%, Aladdin), sodium sulfate anhydrous (Na_2_SO_4_, ≥ 99.0%, Sinopharm-SCR), iodine (I_2_, 99.99%, Aladdin), ethanol absolute (C_2_H_5_OH, ≥ 99.7%, Sinopharm-SCR) and acetone (CH_3_COCH_3_, ≥ 99.7%, Sinopharm-SCR) were from commercial and used without further treatment. Deionized water with an 18.25 MΩ/cm resistivity was also used in all experiments.

## 1.2. Catalysts synthesis

*Synthesis of tubular g-C_3_N_4_ (TCN):* The TCN was fabricated by a high-temperature calcination method using a mixture of melamine and urea as the precursor. In a typical synthetic process, 10 g of urea and 1 g of melamine were taken and placed in an agate mortar, ground thoroughly for 40 min, and then the uniform mixture was loaded into a tablet press (Shimadzu, S72-120KN, Japan) and compacted into cylinder sheets (approximately Ø 18 mm × 7 mm) under a certain pressure. Then the prepared cylinder sheets were put into a covered alumina ark and then annealed at a heating rate of 5 °C/min in an air atmosphere at 550 °C in a muffle furnace for 4 h. After cooled naturally to room temperature, the pale-yellow solid was taken out and ground into a fine powder, collected for standby and denoted as TCN-X, where X = 1, 2, 3, 4, and 5 represent the pressure of 48, 56, 64, 72, and 80 Mpa, respectively. Unless otherwise specified, the TCN was regarded as TCN-4 in the subsequent discussions.

*Synthesis of bulk g-C_3_N_4_ (CN):* CN was also prepared as a comparison sample, and the synthesis method was consistent with TCN except for no pressure application.

*Synthesis of single-atom Tm-modified tubular g-C_3_N_4_ with carbon vacancies (TCN-Cv/Tm-Y):* TCN-Cv/Tm-Y was prepared by an impregnation and secondary high-temperature annealing method. Typically, 100 mg of TCN powder was uniformly dispersed in 30 mL of deionized water after 30 min of ultrasound treatment. Then, a certain amount of TmCl_3_·6H_2_O aqueous solution (10 mg/mL) was added dropwise under intense magnetic stirring, and stirring continued for 3 h. After stirring, the suspension was quickly frozen with liquid nitrogen and dried in a freeze-dryer. After freeze-drying, an appropriate amount of semi-finished product was placed into a covered quartz ark and then annealed at a heating rate of 5 °C/min in a 5% H_2_/Ar atmosphere (80 mL/min) for 3 h in a 300 °C tube furnace. After cooling naturally to room temperature, the resultant product was washed several times with anhydrous ethanol and deionized water, and then freeze-dried. The final obtained catalyst was named TCN-Cv/Tm-Y, where Y = 1, 2, and 3 respectively represent the addition of 300, 600, and 900 μL of TmCl_3_·6H_2_O aqueous solution.

## 1.3. Materials characterization

The X-ray diffraction (XRD) patterns of these as-fabricated samples were recorded by an X-ray diffractometer (TD-3500, Dandong Tongda Science & Technology Co., Ltd, China) with Cu-Kα radiation (λ = 1.5418 Å, at 30 kV and 20 mA). The testing range was from 10° to 80° with a scanning rate of 4.8 °/min. The surface properties of as-obtained specimens were obtained by a Fourier transform infrared spectroscopy (FTIR; NEXUS870, NICOLET, USA). The morphology and microstructure of all samples were obtained using an ultra-high resolution field emission scanning electron microscope (FESEM; GeminiSEM 500, Carl Zeiss, Germany) equipped with energy-dispersive X-ray spectroscopy (EDS; Ultim Extreme, Oxford Instruments, UK) detector operated at 0.02-30 kV and a TECNAI F20 transmission electron microscope (TEM; FEI, USA) operated at 200 kV. The selected area electron diffraction (SAED) patterns were collected on the same TEM instrument. The surface elemental composition and electronic structure of the as-synthesized catalysts were determined by an X-ray photoelectron spectroscopy (XPS; Thermo Scientific K-Alpha, Thermo Fisher Scientific, USA) equipped with Al Kα monochromatic X-ray source operated at 12 kV. The binding energy of the C 1s (284.80 eV) was taken as the energy standard for charge correction. Electron paramagnetic resonance (EPR) signals were measured at ambient temperature by using a Bruker EMXplux spectrometer (Bruker BioSpin, Germany). The aberration-corrected high-angle annular dark-field scanning transmission electron microscopy (HAADF-STEM) characterization as well as energy-dispersive X-ray spectroscopy (EDS) mapping images were obtained using a transmission electron microscope equipped with a double spherical aberration corrector (Titan3 Cubed G2 60-300, FEI, USA) operated at 300 kV, which located at Center for the Microstructures of Quantum Materials, Nanjing University, Jiangsu. Organic elemental analysis (OEA) was carried out on a Vario MICRO cube (Elementar, Germany). The UV-vis diffuse reflectance spectroscopy (UV-vis DRS) of catalysts was examined on a UV-2550 spectrophotometer (Shimadzu, Japan) with BaSO_4_ as the reference background. Meanwhile, the absorption spectra were obtained based on the Kubelka-Munk function. The N_2_ adsorption-desorption isotherms of catalysts were measured by a TriStar 3000 automatic specific surface area and porosity analyzer (Micromeritics, USA) at 77 K to obtain Brunauer-Emmett-Teller (BET) specific surface area and pore size distribution. The CO_2_ adsorption isotherms of the samples were measured in the relative pressure range of 0.00-0.03 at 273.15 K on the same TriStar 3000 instrument. Photoluminescence (PL) emission spectra were collected using an SR-500i-B1-R fluorescence spectrometer (ANDOR, UK) at 405 nm. In addition, the time-resolved fluorescence (TRF) emission decay spectra of samples were recorded on a PicoHarp 300 Time-Correlated Single Photon Counter (TCSPC) spectrometer (PicoQuant, GER). The *in-situ* Fourier transform infrared spectroscopy (ISFTIR) of catalysts was measured using the INVENIO R FT-IR spectrometer (Bruker, Germany) equipped with an in situ diffuse reflectance cell (Harrick). The actual thulium content of each catalyst was quantified using an inductively coupled plasma-atomic emission spectrometer (ICP-OES; OPTIMA5300DV, PE, USA). The detection of the ^13^C isotope in ^13^CO was conducted using mass spectrometry (QP2010SE, Shimadzu, Japan). The hydrophilic/hydrophobicity tests were performed on an SDC-350KS contact angle (CA) measuring instrument (SINDIN, China). The test method was the Sessile Drop method and the angle measurement method was Young-Laplace equation fitting. The X-ray absorption fine structure (XAFS) spectra at the Tm L_3_-edge of the materials were operated at beamline BL14W1 station in Shanghai Synchrotron Radiation Facility (SSRF, located at 239 Zhangheng Road, Pudong New District, Shanghai, China). The radiation was monochromatized through a Si (311) monochromator. The data were obtained in fluorescence excitation mode. Tm_2_O_3_ and Tm powder were used as references. Data reduction, data analysis, and extended X-ray absorption fine structure (EXAFS) fitting were applied through Athena and Artemis software.^[1]^ The energy calibration of the sample was conducted through a standard Tm powder, which as a reference was simultaneously measured. Wavelet transformation (WT) was also employed using the software package developed by Funke and Chukalina using Morlet wavelet with κ = 10, σ = 1.^[2]^

## 1.4. Evaluation of CO_2_ photo-reduction performance

Photocatalytic CO_2_ reduction experiments were conducted in a Pyrex reaction vessel with a volume of approximately 460 mL at room temperature. A Xe arc lamp (PLS-SXE300+, Perfectlight, China) was utilized as the light source. During the testing period, the temperature of the reaction system was constantly maintained at room temperature by the circulating cooling water device. Typically, before illumination, the as-fabricated photocatalyst (5 mg) was evenly dispersed on the quartz glass plate with an area of 3.14 cm^2^, and 0.4 mL deionized water was added into the Pyrex reactor as a reducer. Then, the reactor was sealed using a quartz glass top, and subsequently, the high-purity CO_2_ gas was injected into the gas-tight reaction system to exhaust air and reach ambient pressure. Finally, the as-prepared catalysts were equilibrated at dark in the CO_2_/H_2_O atmosphere for several hours to guarantee the adequate adsorption of gas molecules. Starting from the Xe arc lamp was turned on, about 1 mL of the gas was continually aspirated from the reactor every hour and the reaction products were detected by using a GC-2014 gas chromatograph (Shimadzu Corp., Japan) equipped with a flame ionization detector (FID) and thermal conductivity detector (TCD). The catalytic performance of each catalyst was tested three times in parallel with samples prepared from different batches, and the average value was taken as the test result. Using the TCN-Cv/Tm-2 normal test as a reference, control experiments were carried out under conditions of no catalyst, no CO_2_, no H_2_O, no light, or Ar instead of CO_2_. For example, when tested under catalyst-free conditions, only no catalyst is added compared to the normal test conditions of TCN-Cv/Tm-2, and other conditions remain unchanged. The photocatalytic cyclic stability test on CO and CH_4_ production over TCN-Cv/Tm-2 for four cycles was carried out according to the above catalytic activity test steps. Each cycle was tested for six hours.

The CO evolution rate was calculated according to the following equation:

$$Evolution rate \left( \mu mol \text{g}^{\text{-1}} \text{h}^{\text{-1}} \right)=\frac{Yield over a period of time (\mu mol)}{Mass of catalyst (g)\times Time (h)}$$

The CO selectivity was calculated according to the following equation:

$$CO selectivity=\frac{Evolution rate of CO}{Evolution rate of CO+Evolution rate of C\text{H}_{\text{4}}}\times100\%$$

The apparent quantum efficiency (AQE) was calculated according to the following equation:^[3]^

$$\mathrm{AQE}\left( \% \right)=\frac{N\left( \mathrm{electron} \right)}{N\left( \mathrm{photon} \right)}=\frac{2\times N\left( \mathrm{CO} \right)+8\times N(\mathrm{CH}_{4})}{N\left( \mathrm{photon} \right)}\times100\%$$

where N (electron) signifies that it consumes 2 (or 8) electrons to produce one CO (or CH_4_) molecule in a unit of time. N (CO) and N (CH_4_) indicate the number of CO and CH_4_ molecules generated in the reaction, respectively. The N (photon) refers to the number of incident photons, which is figured out by the following formula:

$$N\left( \mathrm{photon} \right)=\frac{Light intensity\times Illumination area\times Time}{Average single photon energy\times N_{A}}$$

in which a light-emitting diodes (LEDs) with 385 nm wavelength was used as monochromatic incident light. The light intensity of LEDs is 2.21 mW/cm^2^, the illumination area is controlled at 3.14 cm^2^, N_A_ refers to the Avogadro constant, Time is effective illumination time, as well as the average single photon energy (E (photon)) is estimated based on the equation:

$$E(photon)=\frac{h\times c}{\lambda}$$

where h refers to the Planck constant, λ indicates the wavelength, and c is the speed of light.

## 1.5. Photoelectrochemical measurements

All the photoelectrochemical measurements were implemented by using a CHI660E electrochemical workstation (CH Instruments, Inc., China) equipped with a standard three-electrode system in 0.5 M Na_2_SO_4_ solution. A saturated Ag/AgCl electrode was utilized as the reference electrode and a Pt foil electrode was used as the counter electrode. The working electrode was fabricated by electrophoretic deposition (EPD) method on fluoride tin oxide (FTO) glass. Before EPD, FTO glass was cleaned successively by ultrasonication in toluene, acetone, ethanol as well as deionized water for 30 min, respectively, and then dried at 60 °C. The detailed preparation process is as follows: Firstly, 30 mg catalyst was uniformly dispersed in a 30 mL of 0.3 mg/mL I_2_/acetone solution under ultrasonic treatment. Then, a two-electrode process was applied to deposit the photocatalyst at an applied potential of 30 V for 3 min, where FTO glasses with a coated area of approximately 1×1 cm^2^ were utilized for both electrodes. In the end, the working electrode was obtained after being dried in air at 100 °C for 30 min to remove the residual I_2_.

## 1.6. Density functional theory (DFT) calculations

The spin-polarized DFT calculations were performed in the Vienna Ab-initio Simulation Package (VASP 6.1.0).^[4]^ The projector-augmented wave (PAW) approach^[5]^ and the Perdew-Burke-Ernzerhof (PBE) exchange-correlation functional^[6]^ were used for all computations. Need to mention, that the valency of the Tm atomic sphere should be 9 (ZVAL value in POTCAR). A plane wave cutoff energy was set to 400 eV and the DFT-D3 method^[7]^ was adopted for the van der Waals correction. In addition, a vacuum space of 20 Å along the z direction was set for the catalyst layer to ensure sufficient vacuum and eliminate the periodic interference in the presence of surface adsorption. The convergence thresholds for energy and atomic forces were set as 10^-5^ eV and 0.02 eV/Å, respectively. The optimized catalyst models were frozen and only adsorbates were relaxed in all energy calculations. The thermal and zero-point energy corrections of different intermediates adsorbed on constructed catalysts were calculated at the Γ point. The Gibbs free energy change (ΔG)^[8]^ is simply defined as:

$$\Delta G=\Delta E+{\Delta E}_{\mathrm{ZPE}}-T\Delta S$$

where ΔE is the change of reaction free energy directly obtained from DFT total energies, ${\Delta E}_{\mathrm{ZPE}}$ is the change of zero-point energy, T is the temperature (298.15 K), and ΔS is the change in entropy.

The strength of the interaction between the adsorbed molecule and the substrate was described by the adsorption energy (E_ads_), which is defined as:

$$\text{E}_{\text{ads}}=\text{E}_{\text{adsorbate-adsorbent}}-\left( \text{E}_{\text{adsorbate}}+\text{E}_{\text{adsorbent}} \right)$$

Where E_adsorbate-adsorbent_ is the total energy of the adsorbates with adsorbents in their equilibrium geometry, E_adsorbate_ is the energy of an isolated adsorbate molecule, and E_adsorbent_ is the energy of the optimized adsorbent. Therefore, the more negative the value of E_ads_, the stronger the adsorption.

# 2. Supplementary data

***2.1. Schematic diagram for synthesis***


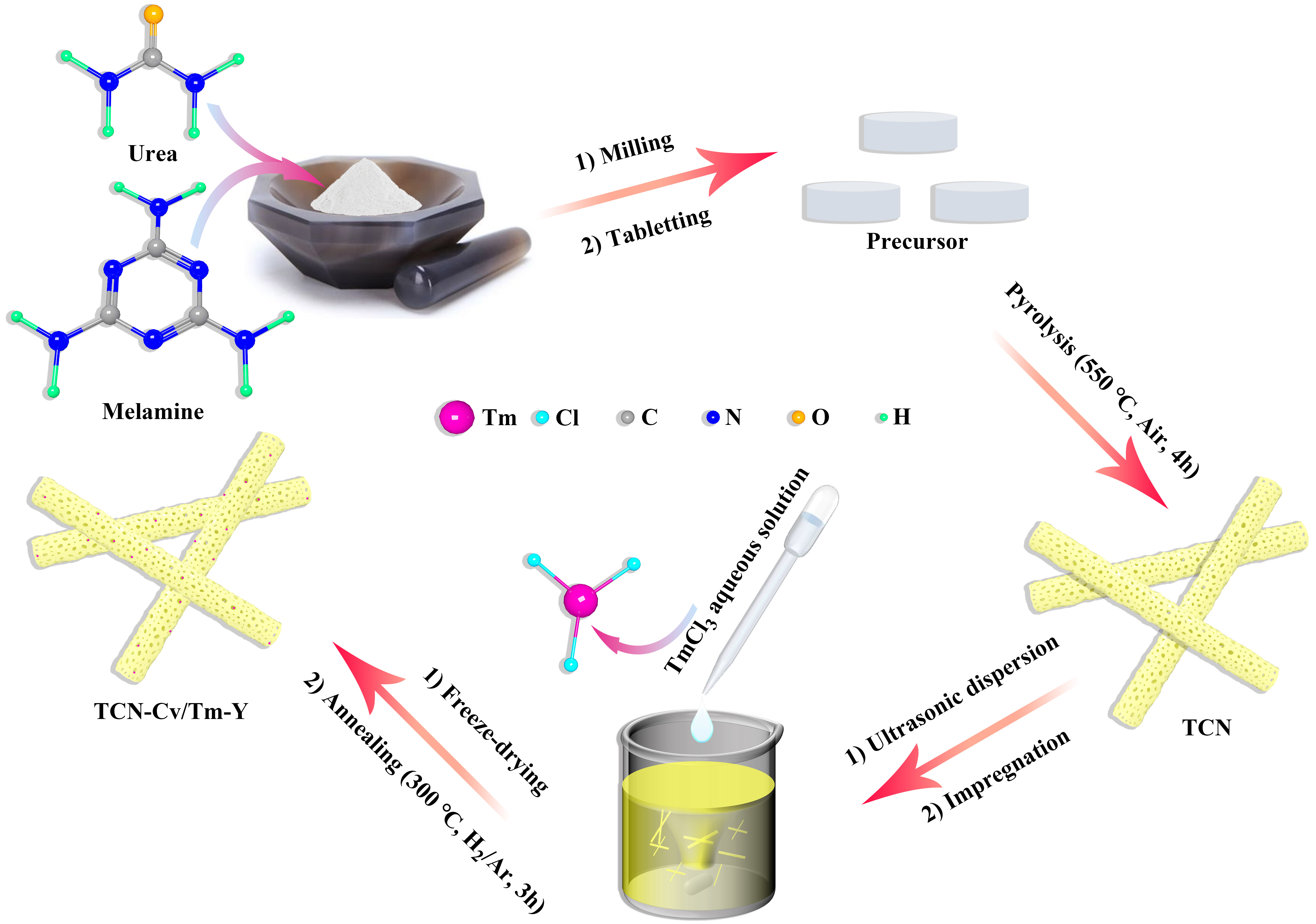


**Figure S1.** The schematic diagram of TCN and TCN-Cv/Tm-Y (Y = 1, 2, or 3) photocatalysts synthesis.

***2.2. SEM (EDS), TEM (SAED), and XPS analysis of TCN***

**
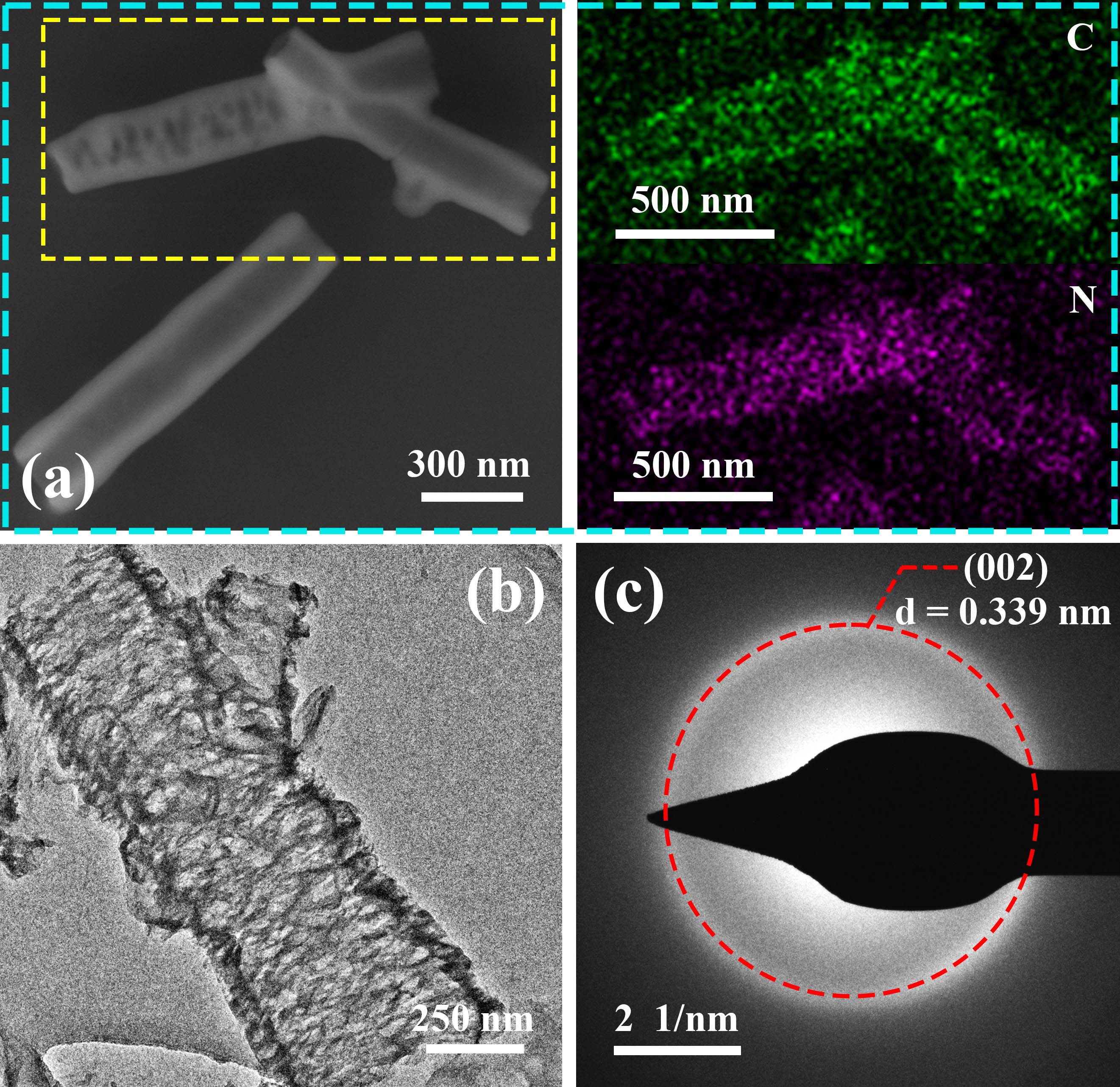
**

**Figure S2.** (a) SEM image and EDS elemental maps, (b) TEM image, and (c) SAED image of TCN.

**
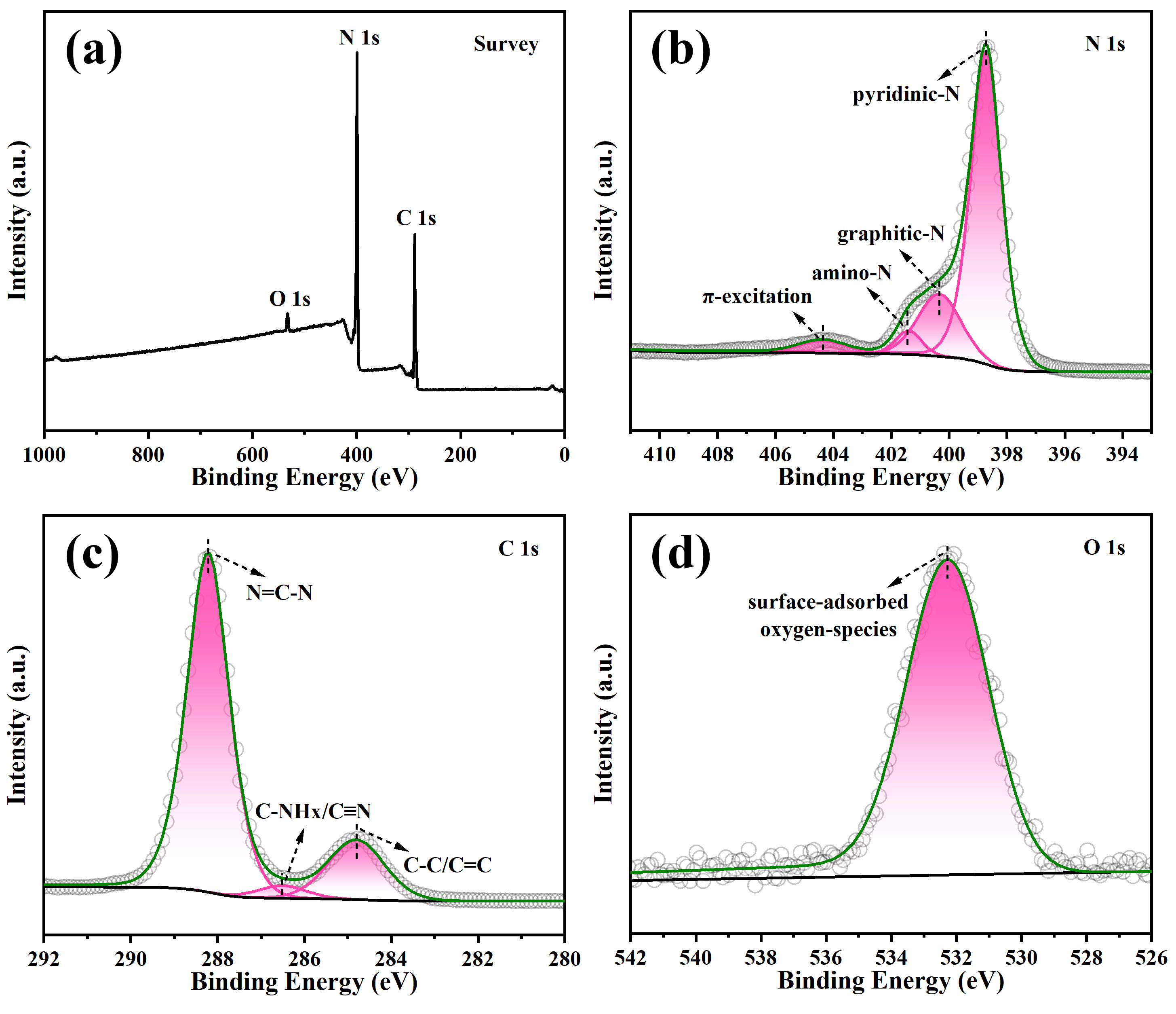
**

**Figure S3.** XPS spectra of TCN. (a) XPS survey spectrum, (b) high-resolution N 1s XPS spectrum, (c) high-resolution C 1s XPS spectrum, and (d) high-resolution O 1s XPS spectrum.

***2.3. XRD, SEM, TEM (SAED), and XPS analysis of CN***

**
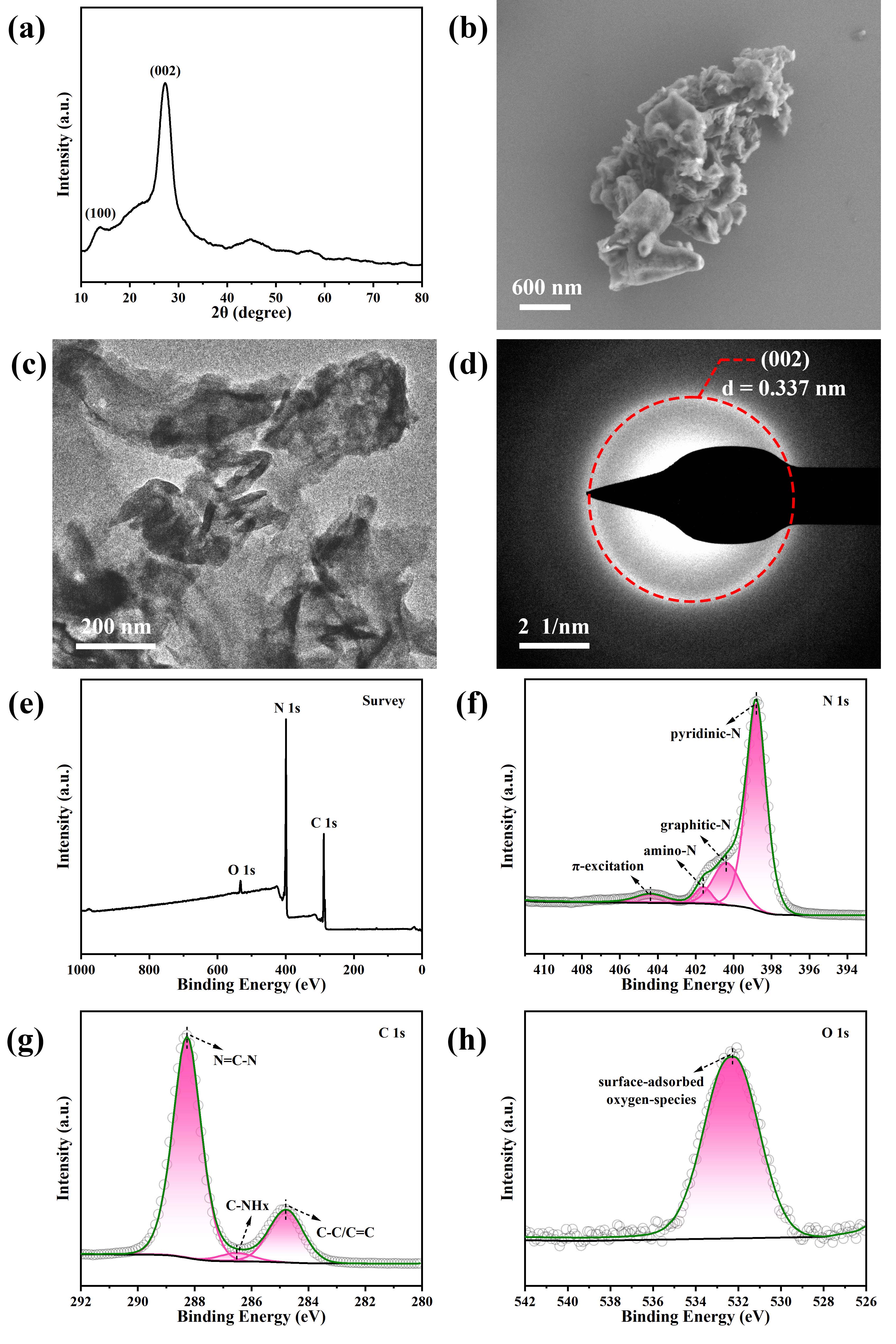
**

**Figure S4.** (a) XRD pattern, (b) SEM image, (c) TEM image, (d) SAED image, and (e-h) XPS spectra of CN.

***2.4. Table of Tm content analysis***

**Table S1.** The Tm loading mass results of TCN-Cv/Tm-Y (Y = 1, 2, or 3) catalysts were measured by ICP-OES and XPS.

| **Catalysts** | TCN-Cv/Tm-1 | TCN-Cv/Tm-2 | TCN-Cv/Tm-3 |
| --- | --- | --- | --- |
| **Tm (wt%) (ICP-OES)** | 0.79 | 1.65 | 2.56 |
| **Tm (wt%) (XPS)** | 0.81 | 1.45 | 1.62 |

***2.5. XRD, SEM, and TEM characterization of TCN-Cv/Tm-Y (Y = 1, 2, or 3)***

**
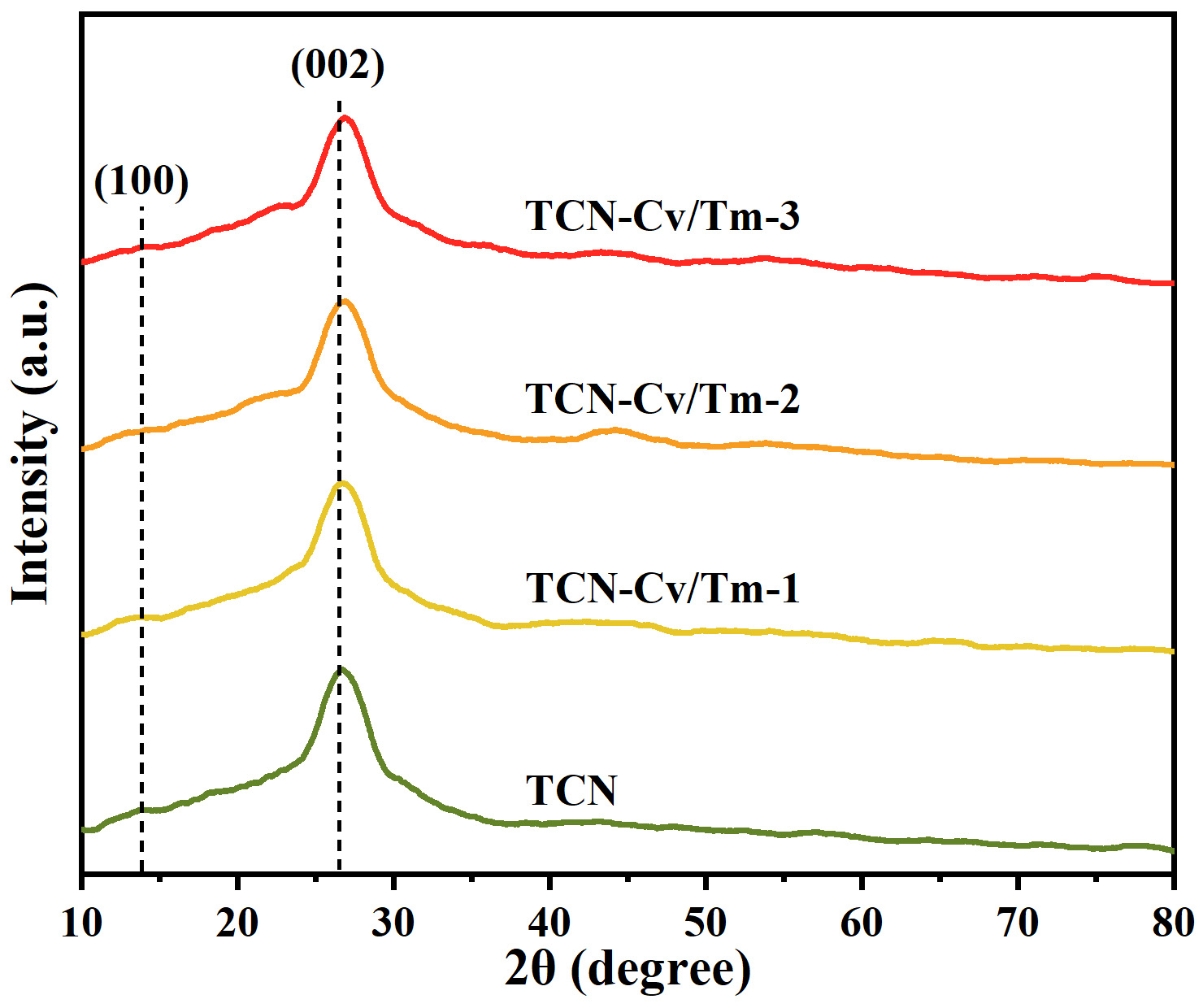
**

**Figure S5.** XRD patterns of TCN and TCN-Cv/Tm-Y (Y = 1, 2, or 3) samples.

**
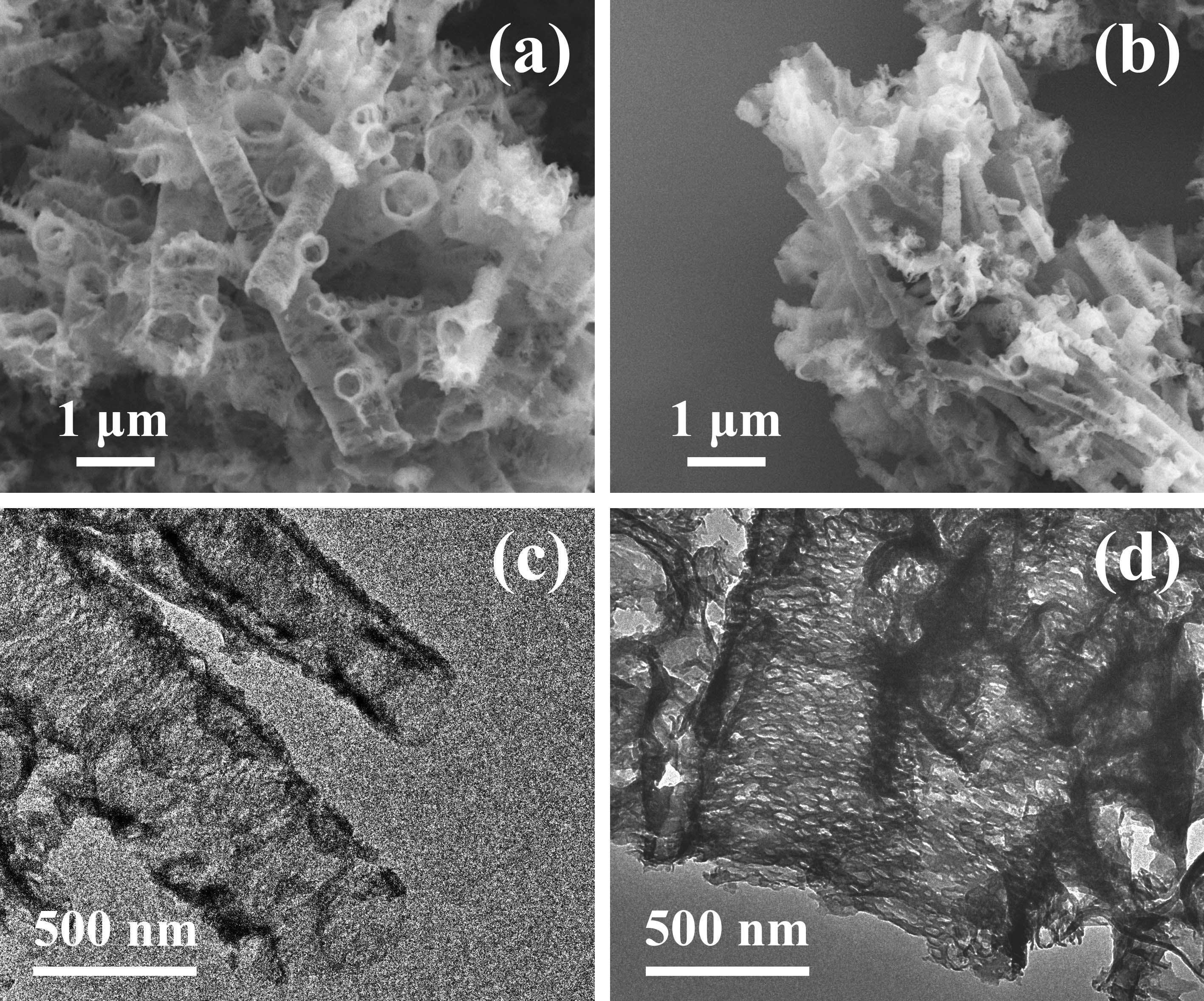
**

**Figure S6.** SEM and TEM images of (a, c) TCN-Cv/Tm-1 and (b, d) TCN-Cv/Tm-3 samples.

***2.6. AC HAADF-STEM images of TCN-Cv/Tm-2***


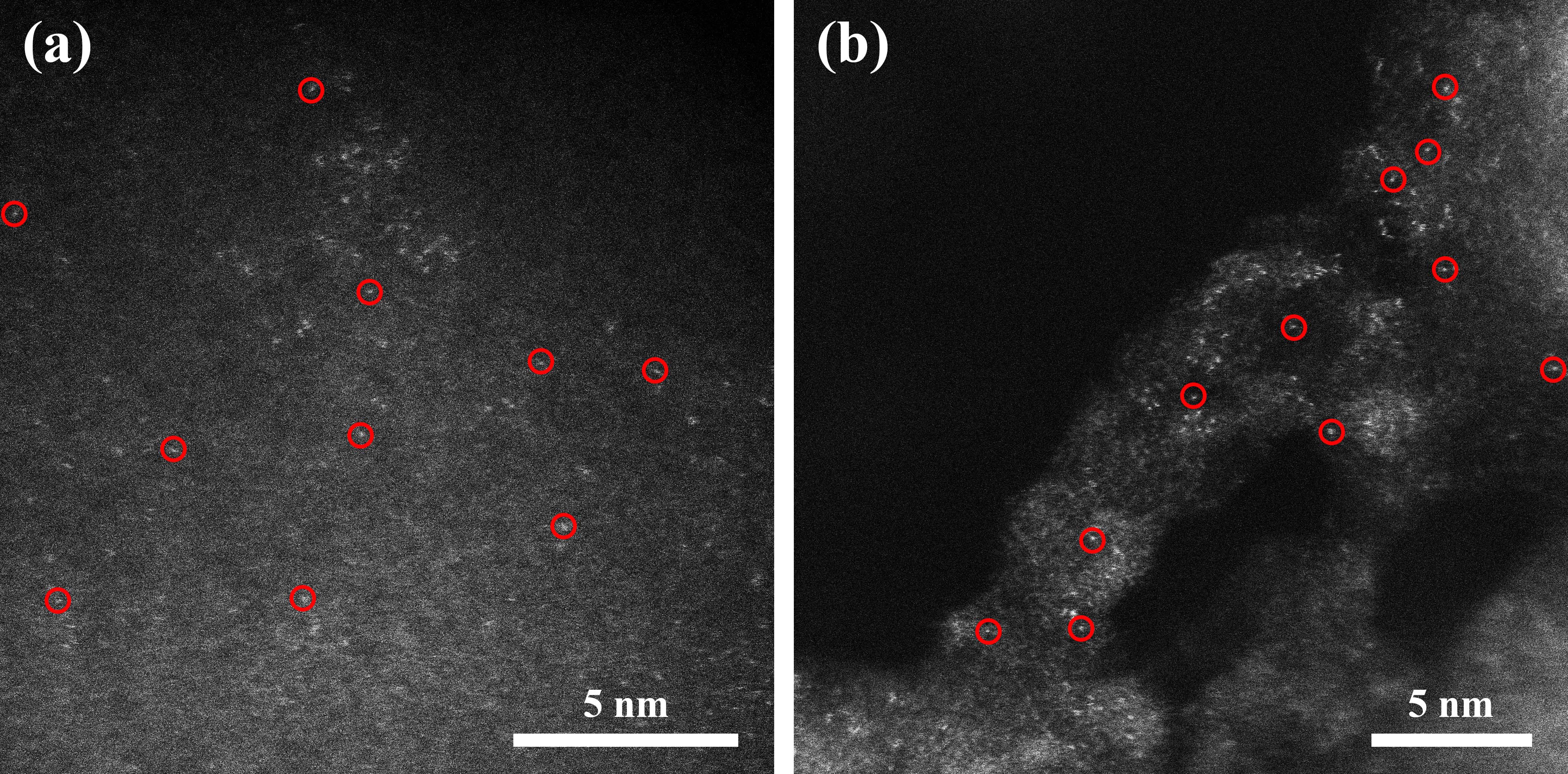


**Figure S7.** (a, b) AC HAADF-STEM images of TCN-Cv/Tm-2 in different areas (single-atom Tm species are highlighted by red circles).

***2.7. FTIR and EPR spectra of CN, TCN, and TCN-Cv/Tm-Y***

**
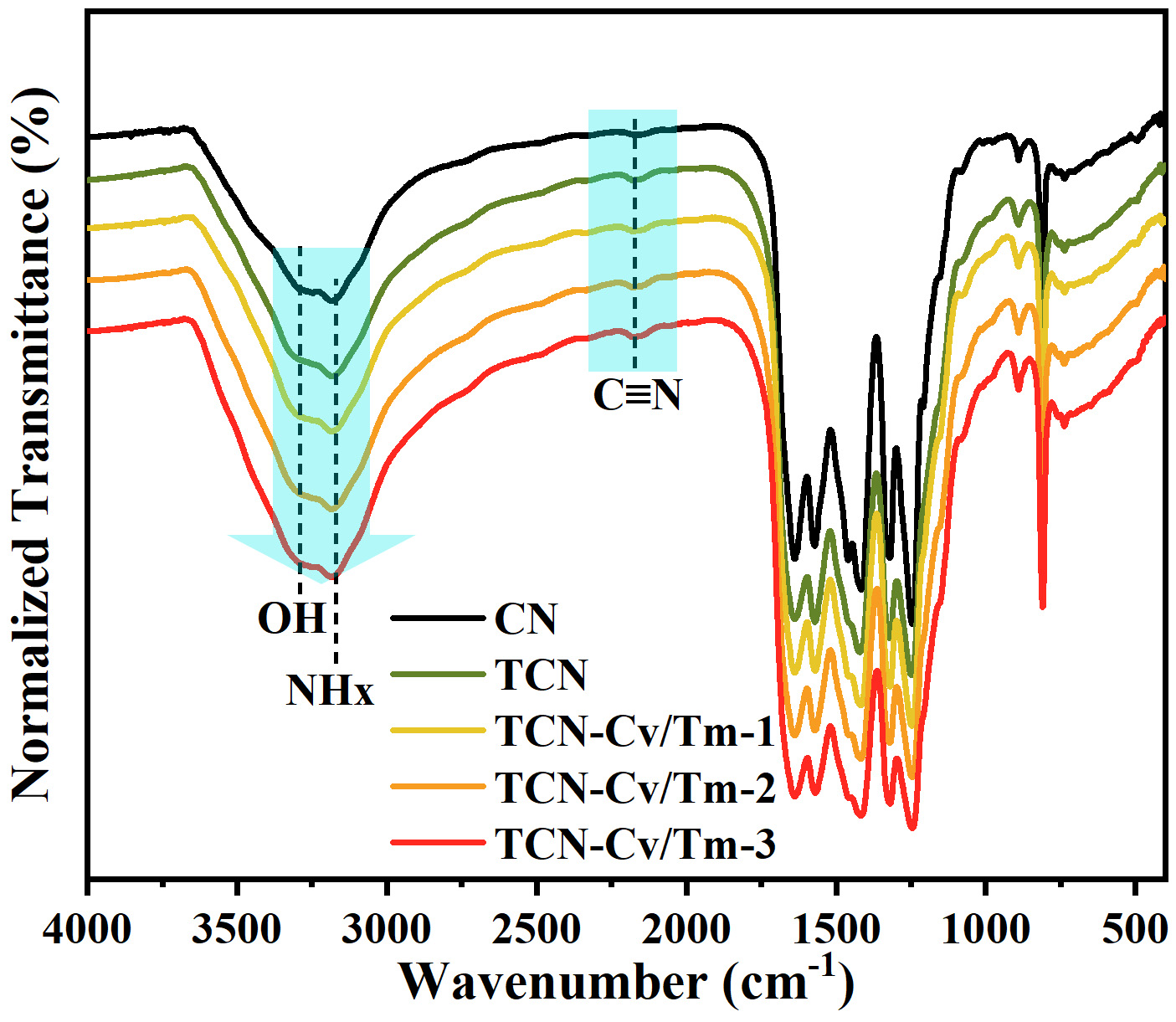
**

**Figure S8.** FTIR spectra of CN, TCN, and TCN-Cv/Tm-Y (Y = 1, 2, or 3) samples.

**
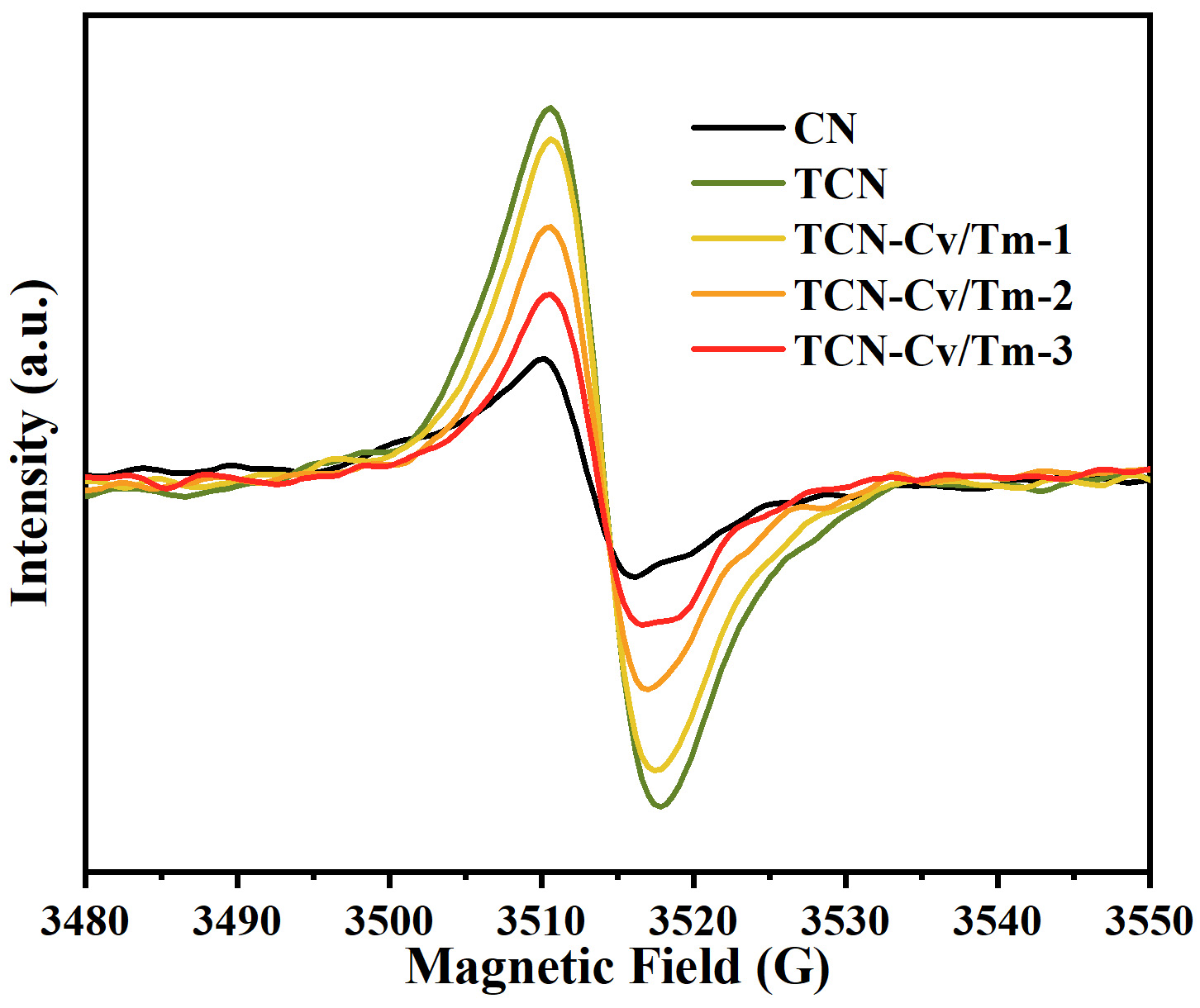
**

**Figure S9.** EPR spectra of CN, TCN, and TCN-Cv/Tm-Y (Y = 1, 2, or 3) samples.

***2.8. XPS analysis of TCN-Cv/Tm-Y***

**
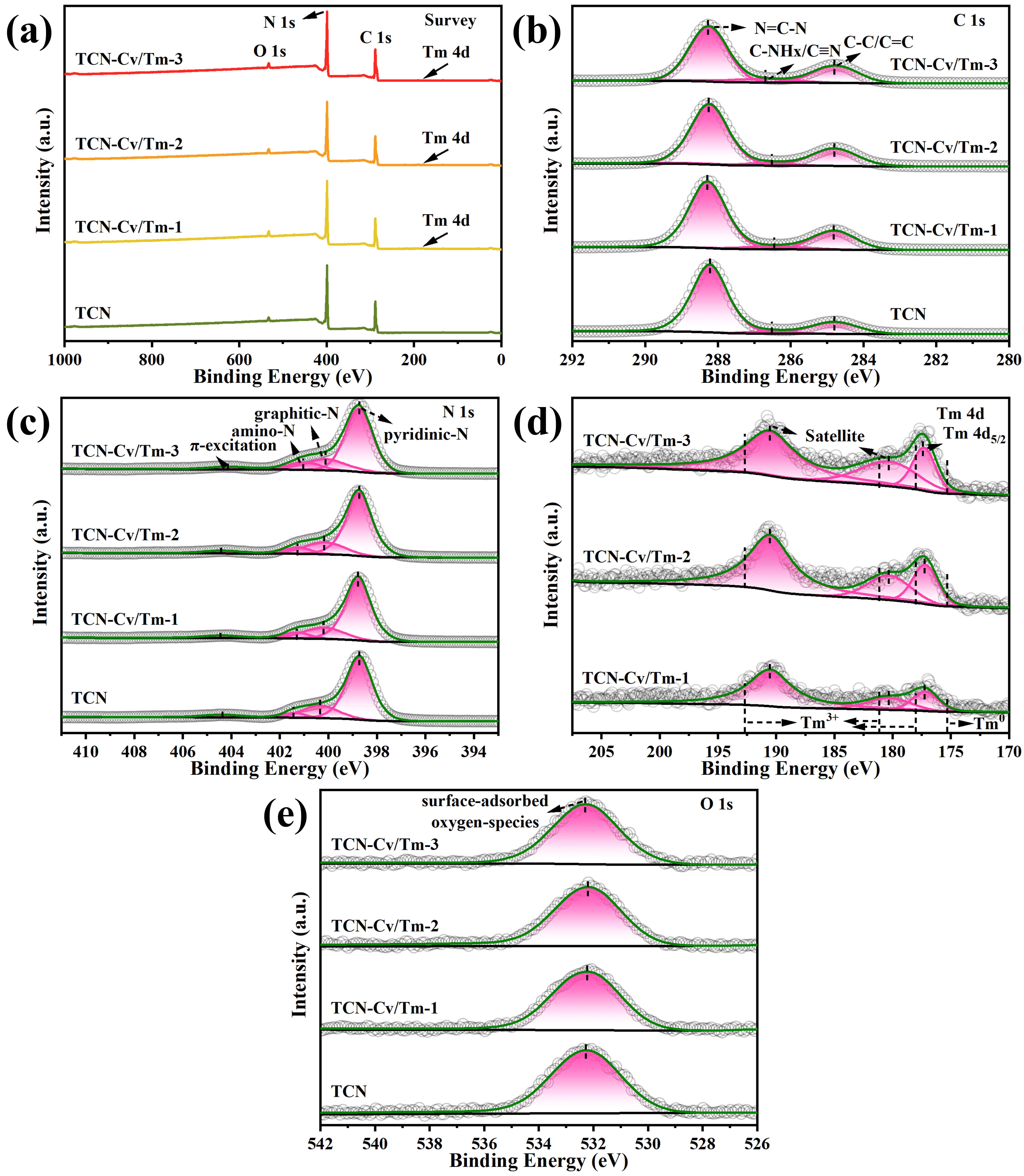
**

**Figure S10.** XPS spectra of TCN and TCN-Cv/Tm-Y (Y = 1, 2, or 3) samples. (a) XPS survey spectra, (b) high-resolution C 1s XPS spectra, (c) high-resolution N 1s XPS spectra, (d) high-resolution Tm 4d XPS spectra, and (e) high-resolution O 1s XPS spectra.

**Table S2.** Summarized C 1s XPS data for TCN and TCN-Cv/Tm-Y (Y = 1, 2, or 3) samples.

| **Samples** | **Carbon species** | **Binding energies (eV)** | **Area ratio** | $\frac{\text{N=C-N}}{\text{C-C/C=C}}$ |
| --- | --- | --- | --- | --- |
| TCN | N=C-N | 288.22 | 0.7991 | 4.69 |
|  | C-NHx/C≡N | 286.52 | 0.0305 |  |
|  | C-C/C=C | 284.80 | 0.1703 |  |
| TCN-Cv/Tm-1 | N=C-N | 288.28 | 0.7301 | 3.33 |
|  | C-NHx/C≡N | 286.45 | 0.0505 |  |
|  | C-C/C=C | 284.80 | 0.2194 |  |
| TCN-Cv/Tm-2 | N=C-N | 288.24 | 0.6788 | 2.87 |
|  | C-NHx/C≡N | 286.52 | 0.0850 |  |
|  | C-C/C=C | 284.80 | 0.2363 |  |
| TCN-Cv/Tm-3 | N=C-N | 288.27 | 0.6436 | 2.71 |
|  | C-NHx/C≡N | 286.72 | 0.1192 |  |
|  | C-C/C=C | 284.80 | 0.2372 |  |

**Table S3.** Summarized N 1s XPS data for TCN and TCN-Cv/Tm-Y (Y = 1, 2, or 3) samples.

| **Samples** | **Nitrogen species** | **Binding energies (eV)** | **Area ratio** | $\frac{\text{amino-N}}{\text{graphitic-N}}$ |
| --- | --- | --- | --- | --- |
| TCN | pyridinic-N | 398.72 | 0.7284 | 0.23 |
|  | graphitic-N | 400.34 | 0.1882 |  |
|  | amino-N | 401.40 | 0.0426 |  |
|  | π-excitation | 404.37 | 0.0408 |  |
| TCN-Cv/Tm-1 | pyridinic-N | 398.76 | 0.7087 | 0.34 |
|  | graphitic-N | 400.18 | 0.1878 |  |
|  | amino-N | 401.29 | 0.0636 |  |
|  | π-excitation | 404.40 | 0.0398 |  |
| TCN-Cv/Tm-2 | pyridinic-N | 398.73 | 0.6869 | 0.45 |
|  | graphitic-N | 400.15 | 0.1894 |  |
|  | amino-N | 401.26 | 0.0846 |  |
|  | π-excitation | 404.39 | 0.0391 |  |
| TCN-Cv/Tm-3 | pyridinic-N | 398.73 | 0.6668 | 0.57 |
|  | graphitic-N | 400.10 | 0.1860 |  |
|  | amino-N | 400.98 | 0.1069 |  |
|  | π-excitation | 404.12 | 0.0403 |  |

***2.9. Organic elemental analysis (OEA)***

**Table S4.** The OEA results, N/H, and C/N atomic ratio of TCN and TCN-Cv/Tm-Y (Y = 1, 2, or 3) samples.

| **Samples** | **C**  **(wt%)** | **N**  **(wt%)** | **H**  **(wt%)** | **N/H**  **(mol%)** | **C/N**  **(mol%)** |
| --- | --- | --- | --- | --- | --- |
| TCN | 33.71 | 58.69 | 2.63 | 1.59 | 0.67 |
| TCN-Cv/Tm-1 | 33.21 | 58.74 | 2.78 | 1.51 | 0.66 |
| TCN-Cv/Tm-2 | 32.34 | 58.65 | 2.96 | 1.42 | 0.64 |
| TCN-Cv/Tm-3 | 31.23 | 58.71 | 3.21 | 1.31 | 0.62 |

***2.10. Synchrotron radiation analysis***

**
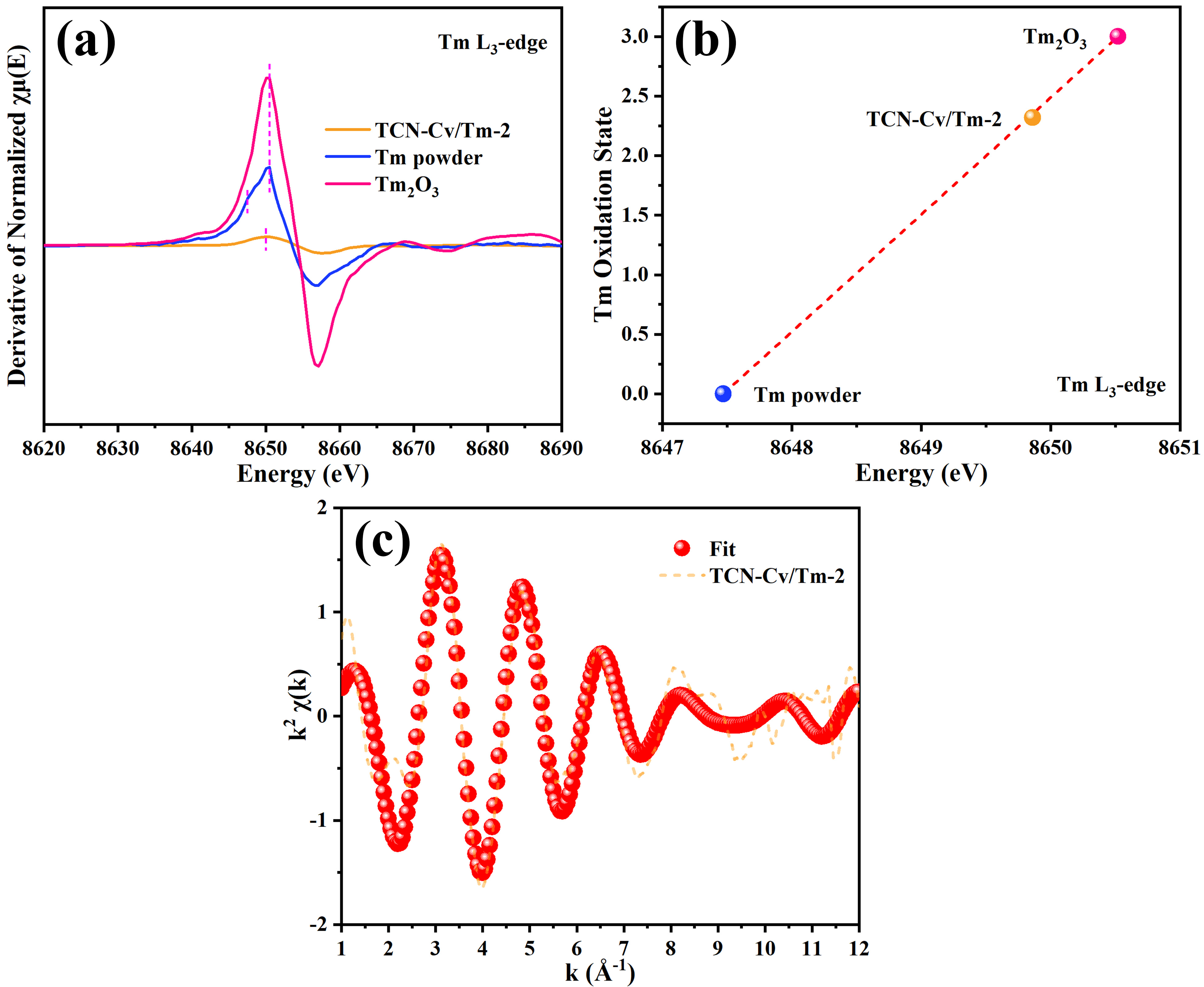
**

**Figure S11.** (a) First derivatives of Tm L_3_-edge XANES regions of Tm powder, Tm_2_O_3_, and TCN-Cv/Tm-2. (b) The fitted average oxidation state of Tm from XANES spectra. (c) Tm L_3_-edge EXAFS spectra and fit curve for the TCN-Cv/Tm-2 sample, shown in k^2^-weighted k-space.

**Table S5.** Fitting structural parameters for Tm L_3_-edge EXAFS for the TCN-Cv/Tm-2 sample.

| **Paths** | **CN** | **R (Å)** | **σ^2^ (Å^2^)** | **R factor** |
| --- | --- | --- | --- | --- |
| Tm-N_1_ | 6.2 ± 0.4 | 2.25 | 0.002 | 0.012 |
| Tm-N_2_ | 6.0 ± 0.4 | 2.41 | 0.002 |  |

Note:

The amplitude reduction factor (S_0_^2^) was obtained from Tm powder and fixed at 0.80. The edge-energy shift (ΔE_0_, namely the difference between the zero kinetic energy value of the theoretical model and that of the sample) returned a value of -0.0885 ± 0.5386 eV. R factor for this fit is 1.20%, which is used to assess the goodness of the fitting. The Debye-Waller factor (σ^2^) is a measure of static disorder and thermal in absorber-scatterer distances. R is interatomic distance, namely the bond length between Tm central atoms and surrounding coordination atoms. CN is the coordination number.

***2.11. XRD and SEM characterization of TCN-X (X = 1, 2, 3, 4, and 5)***

**
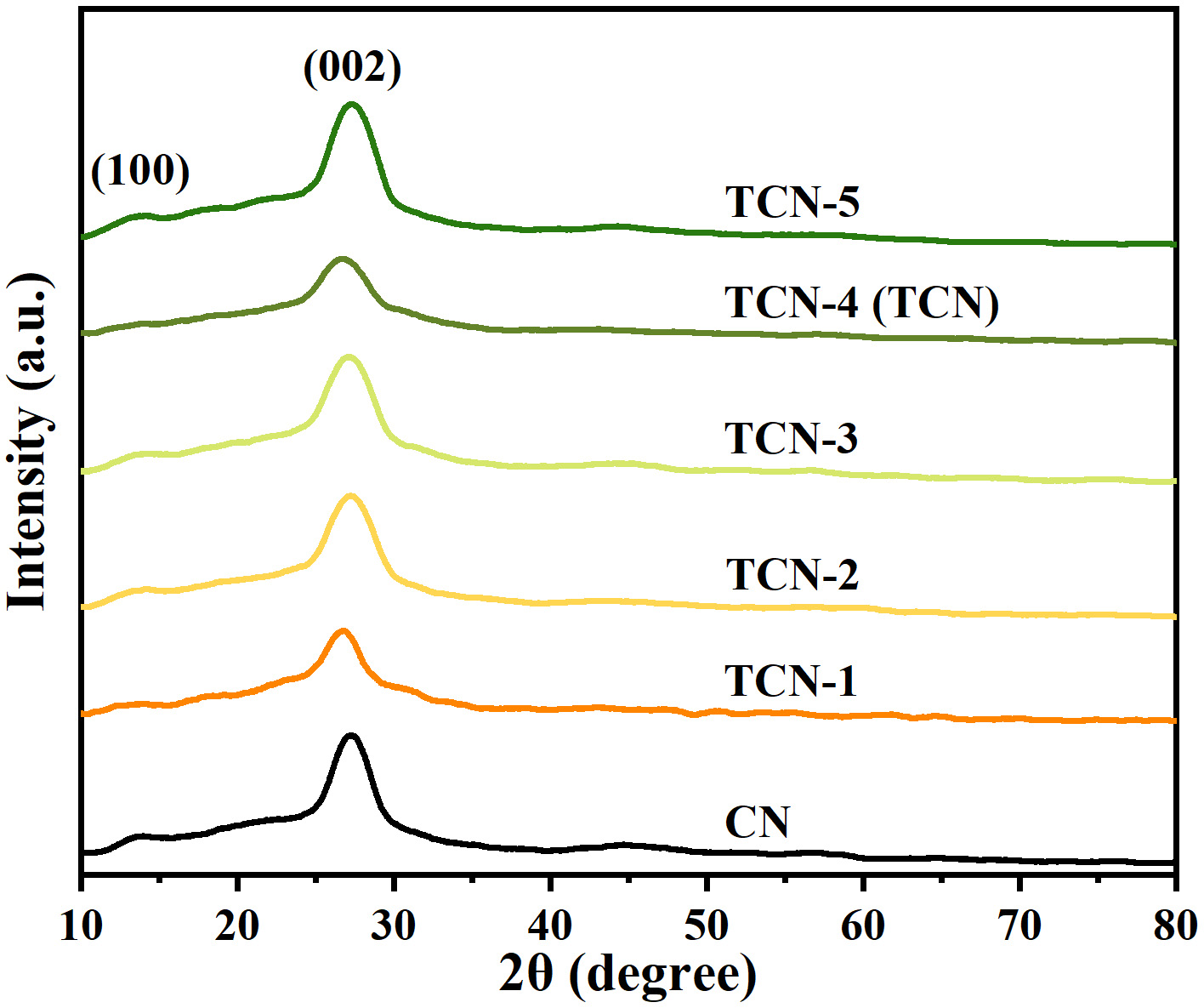
**

**Figure S12.** XRD patterns of CN and TCN-X (X = 1, 2, 3, 4, and 5) samples.

**
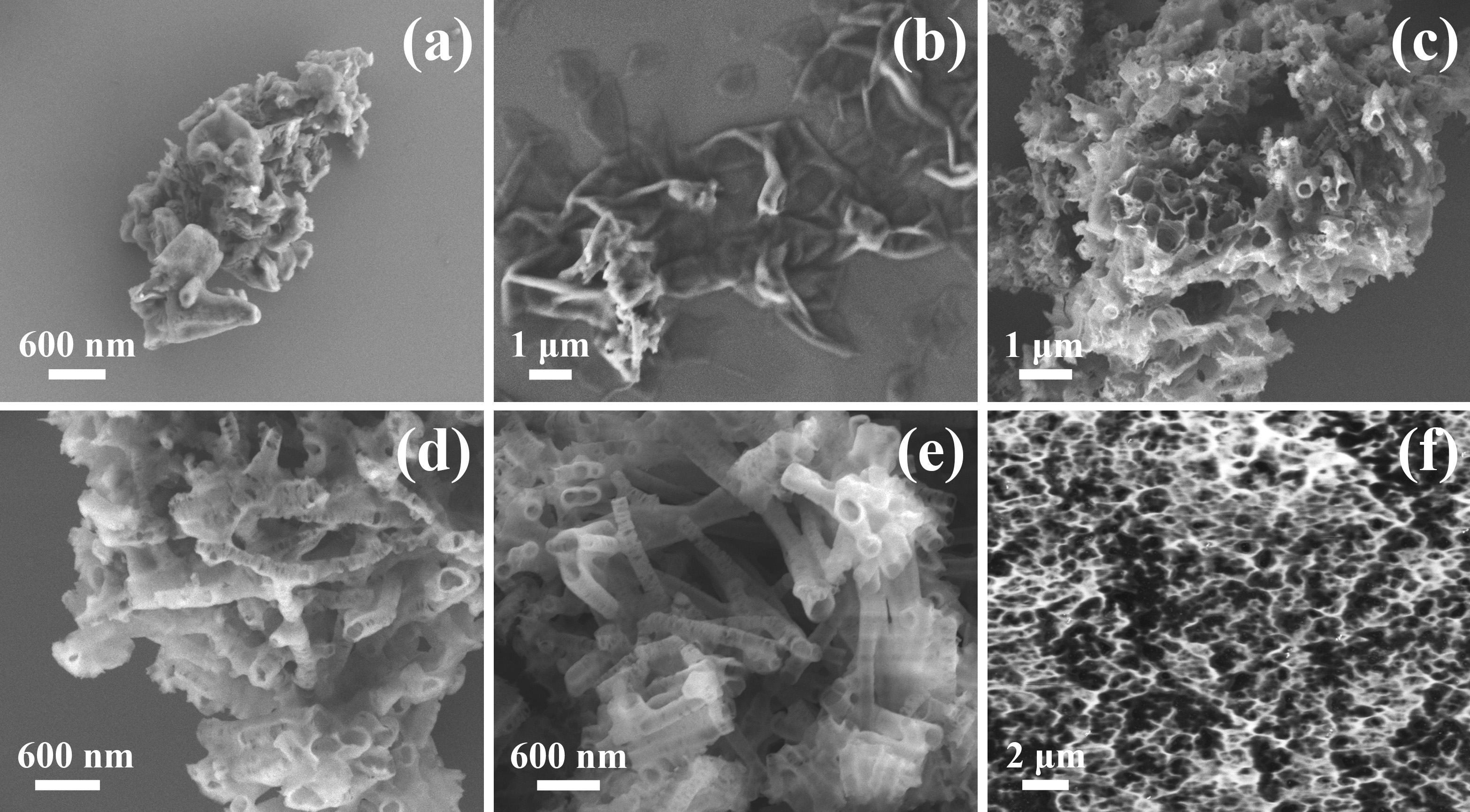
**

**Figure S13.** SEM images of (a) CN, (b) TCN-1, (c) TCN-2, (d) TCN-3, (e) TCN-4 (TCN), and (f) TCN-5 samples.

***2.12. Photocatalytic CO_2_ reduction activity***

**
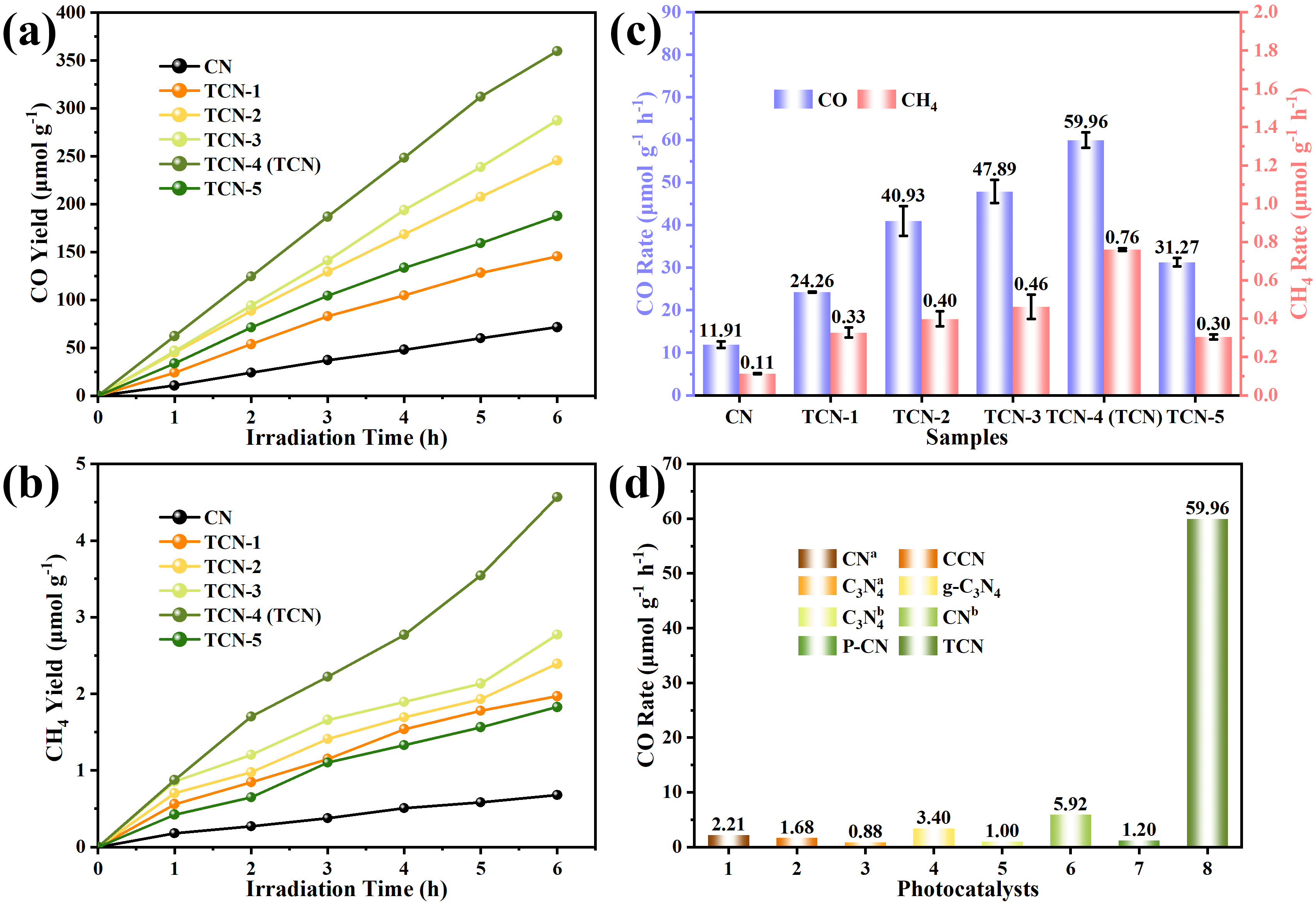
**

**Figure S14.** Photocatalytic CO_2_ reduction activity of CN and TCN-X (X = 1, 2, 3, 4, and 5) samples. (a) The time courses of CO evolution. (b) The time courses of CH_4_ evolution. (c) The average production rates of CO and CH_4_. (d) Comparison of the photocatalytic performances for CO_2_ reduction to CO between TCN and other pristine CN catalysts (catalyst name superscripts refer to differentiate between catalysts in different references, please see Table S6 for specific citations).

**
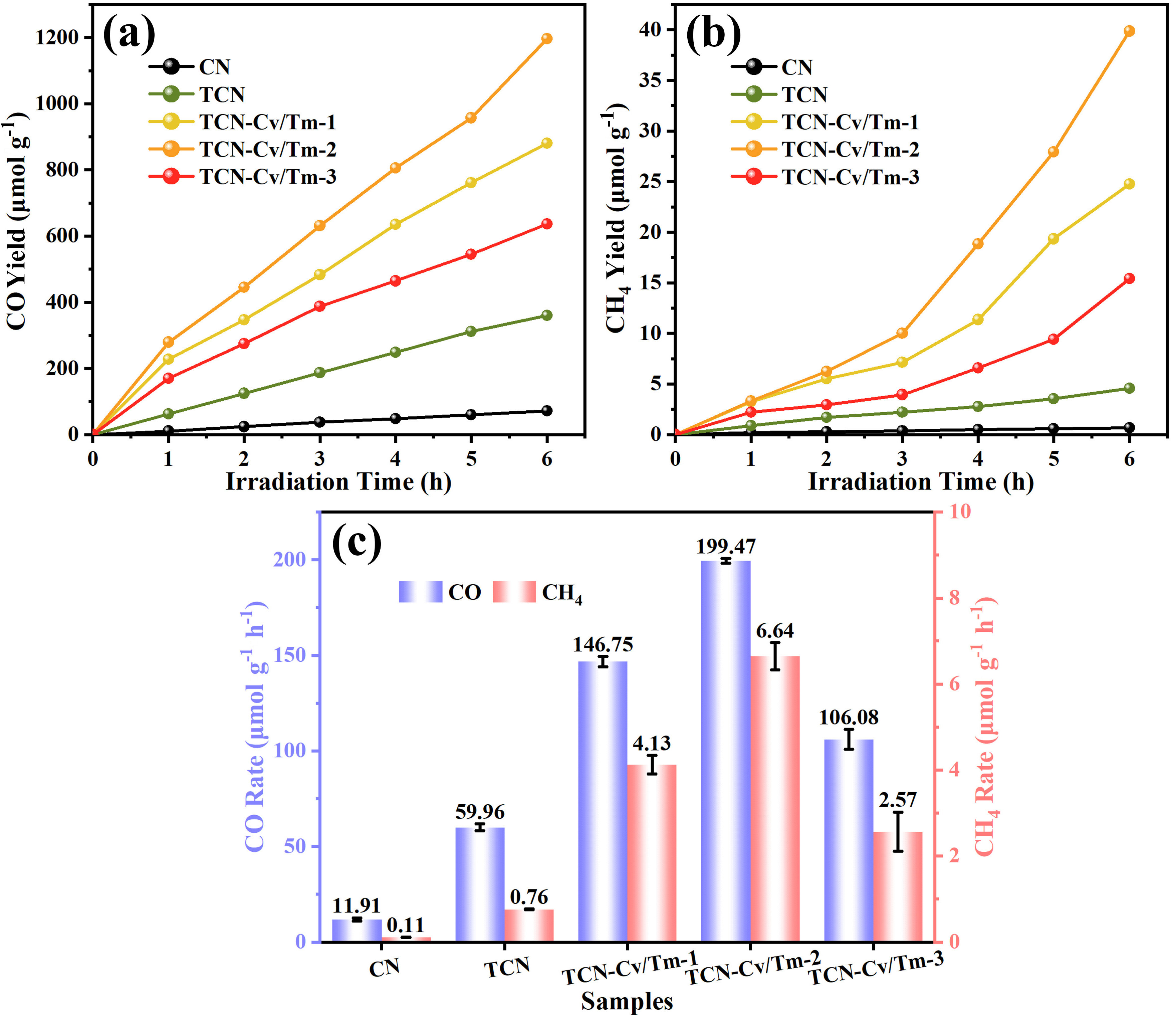
**

**Figure S15.** Photocatalytic CO_2_ reduction activity of CN, TCN, and TCN-Cv/Tm-Y (Y = 1, 2, or 3) samples. (a) The time courses of CO evolution. (b) The time courses of CH_4_ evolution. (c) The average production rates of CO and CH_4_.

***2.13. Tables of performance comparison data***

**Table S6.** Comparison of photocatalytic CO_2_ reduction activity of TCN catalysts with other pristine CN catalysts.

| **Catalysts** | **Light source** | **Reaction condition** | **Major product (CO)**  **evolution rate (μmol g^-1^ h^-1^)** | **Ref./Year** |
| --- | --- | --- | --- | --- |
| CN^a^ | 300 W Xe lamp | CO_2_ + H_2_O (l) | 2.21 | [9]/2021 |
| CCN | 300 W Xe lamp | CO_2_ + H_2_O (g) | 1.68 | [10]/2020 |
| C_3_N_4_^a^ | 300 W Xe lamp  (λ > 420 nm) | CO_2_ + H_2_O (g) | 0.88 | [11]/2022 |
| g-C_3_N_4_ | 300 W Xe lamp with  a 420 nm cut-off filter | CO_2_ + H_2_O (l) | 3.4 | [12]/2021 |
| C_3_N_4_^b^ | 300 W Xe lamp  (λ ≥ 420 nm) | CO_2_ + H_2_O (g) | 1.0 | [13]/2022 |
| CN^b^ | 300 W Xe lamp with  an AM1.5 filter | CO_2_ + H_2_O (g) | 5.92 | [14]/2023 |
| P-CN | 300 W Xe lamp | CO_2_ + H_2_O (g) | 1.2 | [15]/2022 |
| TCN | 300 W Xe lamp | CO_2_ + H_2_O (g) | 59.96 | This work/- |

Note:

The superscripts in CN^a^, C_3_N_4_^a^, C_3_N_4_^b^, and CN^b^ refer to differentiating between catalysts in different references.

**Table S7.** Comparison of photocatalytic CO_2_ reduction activity of TCN-Cv/Tm-2 catalyst with other carbon nitride-based single-atom photocatalysts reported in recent years.

| **Catalysts** | **Light**  **source** | **Reaction condition** | **Major product (CO) evolution rate (μmol g^-1^ h^-1^)** | **CO selectivity (%)** | **Ref./Year** |
| --- | --- | --- | --- | --- | --- |
| O/La-CN  (La single atoms) | 300 W Xe lamp | CO_2_ + H_2_O (l) + TEOA + MeCN | 92 | 80.3 | [16]/2020 |
| High-density  (HD)-Er_1_/CN-NT | 300 W Xe lamp with a 420 nm  filter | CO_2_ + H_2_O (l) | 47.1 | n/a | [17]/2020 |
| Cu-CCN  (Cu single atoms) | 300 W Xe lamp | CO_2_ + H_2_O (g) | 3.086 | ~ 100 | [10]/2020 |
| Fe-g-C_3_N_4_  (Fe single atoms) | 300 W Xe lamp | CO_2_ + H_2_O (l) | 0.51 | n/a | [18]/2021 |
| Cu_1_/N_2C_V-CN-0.5 | 300 W Xe lamp with an AM1.5 filter | CO_2_ + H_2_O (g) | 11.12 | 98.5 | [14]/2023 |
| Cu_1_N_3_@PCN | 300 W Xe lamp (λ ≥ 420 nm) | CO_2_ + H_2_O (g) | ~ 49.8 | > 99 | [13]/2022 |
| Cu/CN-0.25  (Cu single atoms) | 300 W Xe lamp with an AM1.5 filter | CO_2_ + H_2_O (g) | 11.21 | n/a | [19]/2020 |
| Ni/CN-0.5  (Ni single atoms) | 300 W Xe lamp with an AM 1.5G filter | CO_2_ + H_2_O (g) | ~ 19.9 | n/a | [20]/2023 |
| 0.7Ni-5OB-CN  (Ni single atoms) | 300 W Xe lamp | CO_2_ + H_2_O (l) | 22.1 | n/a | [9]/2021 |
| Ni_5_-CN  (Ni single atoms) | 300 W Xe lamp | CO_2_ + H_2_O (g) | 8.6 | 81.1 | [21]/2020 |
| Mn_1_Co_1_/CN | 300 W Xe lamp | CO_2_ + H_2_O (g) | 47 | ~ 100 | [22]/2022 |
| CoRu-HCNp  (Co/Ru dual single atoms) | 300 W Xe lamp | CO_2_ + H_2_O (g) | 27.3 | 91.8 | [23]/2021 |
| PtCu‐crCN  (Pt/Cu dual single atoms) | 300 W Xe lamp | CO_2_ + H_2_O (g) | ~ 11.74 | n/a | [24]/2022 |
| U-CAN  (Au single atoms) | 300 W Xe lamp with a filter (λ > 420 nm) | CO_2_ + H_2_O (g) | ~ 8.68 | n/a | [25]/2020 |
| Ag_1_@PCN | 300 W Xe lamp | CO_2_ + H_2_O (g) | 161.3 | 94.0 | [26]/2023 |
| CN-Cu (Cu single atoms) | Solar simulator with AM 1.5 illumination | CO_2_ + H_2_O (g) | 1.62 | n/a | [27]/2023 |
| Zn_1_/CN | 300 W Xe lamp with an AM1.5 filter | CO_2_ + H_2_O (g) | 76.9 | n/a | [28]/2023 |
| TCN-Cv/Tm-2 | 300 W Xe lamp | CO_2_ + H_2_O (g) | 199.47 | 96.8 | This work/- |

**Table S8.** Comparison of photocatalytic CO_2_ reduction activity of TCN-Cv/Tm-2 catalyst with other outstanding carbon nitride-based photocatalysts reported in the past year.

| **Catalysts** | **Light**  **source** | **Reaction condition** | **Major product (CO) evolution rate (μmol g^-1^ h^-1^)** | **CO selectivity (%)** | **Ref./Year** |
| --- | --- | --- | --- | --- | --- |
| Bro-PCN  (O-doping) | 300 W Xe lamp | CO_2_ + H_2_O (l) + TEOA | 80.93 | n/a | [29]/2023 |
| CNA_2.5_ (A: 5-aminouracil) | 300 W Xe lamp with a filter (λ > 420 nm) | CO_2_ + H_2_O (g) | 12.58 | 96.53 | [30]/2023 |
| PSCN (S-doping) | 300 W Xe lamp with a 420 nm filter | CO_2_ + H_2_O (l) | 30.3 | n/a | [31]/2023 |
| 3% Au/Ar-CN (Ar: O-containing aromatic ring) | 300 W Xe lamp | CO_2_ + H_2_O (g) | 32.03 | n/a | [32]/2023 |
| Cu_1_/N_2C_V-CN-0.5 | 300 W Xe lamp with an AM1.5 filter | CO_2_ + H_2_O (g) | 11.12 | 98.5 | [14]/2023 |
| 20KCSCN | 300 W Xe lamp | CO_2_ + H_2_O (g) | 3.9 | > 80 | [33]/2023 |
| g-C_3_N_4_/Cu_2_SnS_3_ | Solar simulator AM1.5 G | CO_2_ + H_2_O (g) | 18.2 | n/a | [34]/2023 |
| Ni/CN-0.5  (Ni single atoms) | 300 W Xe lamp with an AM 1.5G filter | CO_2_ + H_2_O (g) | ~ 19.9 | n/a | [20]/2023 |
| BCN-NaK | 300 W Xe lamp with a filter (λ > 400 nm) | CO_2_ + H_2_O (l) + MeCN | 22.8 | 61.3 | [35]/2023 |
| BPQDs/CNNT  (BPQDs: black phosphorus quantum dots) | 300 W Xe lamp | CO_2_ + H_2_O (l) + TEOA | 44.56 | 84.2 | [36]/2023 |
| 0.7 at% P-PCN (P-doping) | 300 W Xe lamp with a 420 nm filter | CO_2_ + H_2_O (g) | 5.37 | 83 | [37]/2023 |
| 24-CN-EDA  (EDA: ethylenediamine) | 300 W Xe lamp (420 ~ 780 nm) | CO_2_ + H_2_O (g) | 1.72 | n/a | [38]/2023 |
| Co_1_Ag_(1+n)_-PCN (single atoms and nanoparticles) | 300 W Xe lamp | CO_2_ + H_2_O (l) + MeCN | 11.71 | 70.1 | [39]/2023 |
| Au@CN/Au_c_/G Y@S (yolk@shell structure; nanoparticles and clusters) | 300 W Xe lamp with a filter (λ > 400 nm) | CO_2_ + H_2_O (l) + TEOA | 61.75 | 63.2 | [40]/2023 |
| Co_3_O_4_/Au/PCN-5 (Au nanospecies) | 300 W UV lamp | CO_2_ + H_2_O (l) + TEOA | ~ 39.12 | n/a | [41]/2023 |
| Zn_1_/CN | 300 W Xe lamp with an AM1.5 filter | CO_2_ + H_2_O (g) | 76.9 | n/a | [28]/2023 |
| WO_x_/Cu-g-C_3_N_4_ (Cu nanoparticles) | 300 W Xe lamp | CO_2_ + H_2_O (l) | 5.89 | n/a | [42]/2023 |
| Ag_1_@PCN | 300 W Xe lamp | CO_2_ + H_2_O (g) | 161.3 | 94.0 | [26]/2023 |
| BTCN (thiophene-bridged CN) | 300 W Xe lamp | CO_2_ + H_2_O (g) | 23.02 | n/a | [43]/2023 |
| InVO_4_/g-C_3_N_4_ | PLS-SXE300D | CO_2_ + H_2_O (g) | 20.14 | n/a | [44]/2023 |
| 30.0%Ni(OH)_2_/K-CN (K-doping) | 300 W Xe lamp | CO_2_ + H_2_O (g) | 14.3 | 75.2 | [45]/2023 |
| WO_x_/Pt-g-C_3_N_4_ (Pt clusters) | 300 W Xe lamp (λ > 420 nm) | CO_2_ + H_2_O (l) | 5.89 | n/a | [46]/2023 |
| CN-Cu (Cu single atoms) | Solar simulator with AM 1.5 illumination | CO_2_ + H_2_O (g) | 1.62 | n/a | [27]/2023 |
| TCN-Cv/Tm-2 | 300 W Xe lamp | CO_2_ + H_2_O (g) | 199.47 | 96.8 | This work/- |

**Table S9.** Comparison of photocatalytic CO_2_ reduction activity of TCN-Cv/Tm-2 with other state-of-the-art photocatalysts reported this year.

| **Catalysts** | **Light**  **source** | **Reaction condition** | **Major product evolution rate (μmol g^-1^ h^-1^)** | **Major product selectivity (%)** | **Ref./Year** |
| --- | --- | --- | --- | --- | --- |
| CoAl-LDH/BiOBr-10 (CBO-10) | 300 W Xe lamp | CO_2_ + H_2_O (l) | 23.62 (CO) | 95 | [47]/2024 |
| Cu/Cd_1−x_S | 300 W Xe lamp | CO_2_ + H_2_O (l) | 3.4 (CO) | 98 | [48]/2024 |
| BTOPAu (Bi_4_Ti_3_O_12_ with Au single-atom arrays) | 300 W Xe lamp | CO_2_ + H_2_O (g) | 34.15 (CO) | n/a | [49]/2024 |
| Cu-SA/D-ZIS (Defective ZnIn_2_S_4_-supported single-atom Cu) | 300 W Xe  lamp with a 420 nm cut-filter | CO_2_ + H_2_O (g) | 112.5 (CO) | ~100% | [50]/2024 |
| TiO_2_-HEA (HEA = FeCoNiCuMn high-entropy alloys) | 300 W Xe lamp with an AM  1.5G filter | CO_2_ + H_2_O (g) | 235.2 (CO) | n/a | [51]/2024 |
| Al-CuS/TiO_2_ | 300 W Xe lamp with a 420 nm cutoff filter | CO_2_ + H_2_O (g) + TEOA | 335.68 (CO) | 90.68 | [52]/2024 |
| *V*_N_-CN HNSs (HNSs = holey nanosheets) | 300 W Xe lamp with a 400 nm cut-on filter | CO_2_ + H_2_O (g) | 35.2 (CO) | 100 | [53]/2024 |
| Mn/Co_3_O_4_ | 300 W Xe lamp  (320-780 nm) | CO_2_ + H_2_O (g) | 11.19 (CH_4_) | 82.71 | [54]/2024 |
| DMASnI_3_(O)  (DMA = dimethylammonium) | 300 W Xe arc lamp | CO_2_ + H_2_O (l) | 11.2 (C_2_H_4_) | 74.5 | [55]/2024 |
| Cu-N_2_-V | 300 W Xe lamp | CO_2_ + H_2_O (l) + DMF | 69.8 (C_2_H_5_OH) | 97.8 | [56]/2024 |
| TCN-Cv/Tm-2 | 300 W Xe lamp | CO_2_ + H_2_O (g) | 199.47 (CO) | 96.8 | This work/- |

***2.14. O_2_ evolution rate during CO_2_ reduction***

**
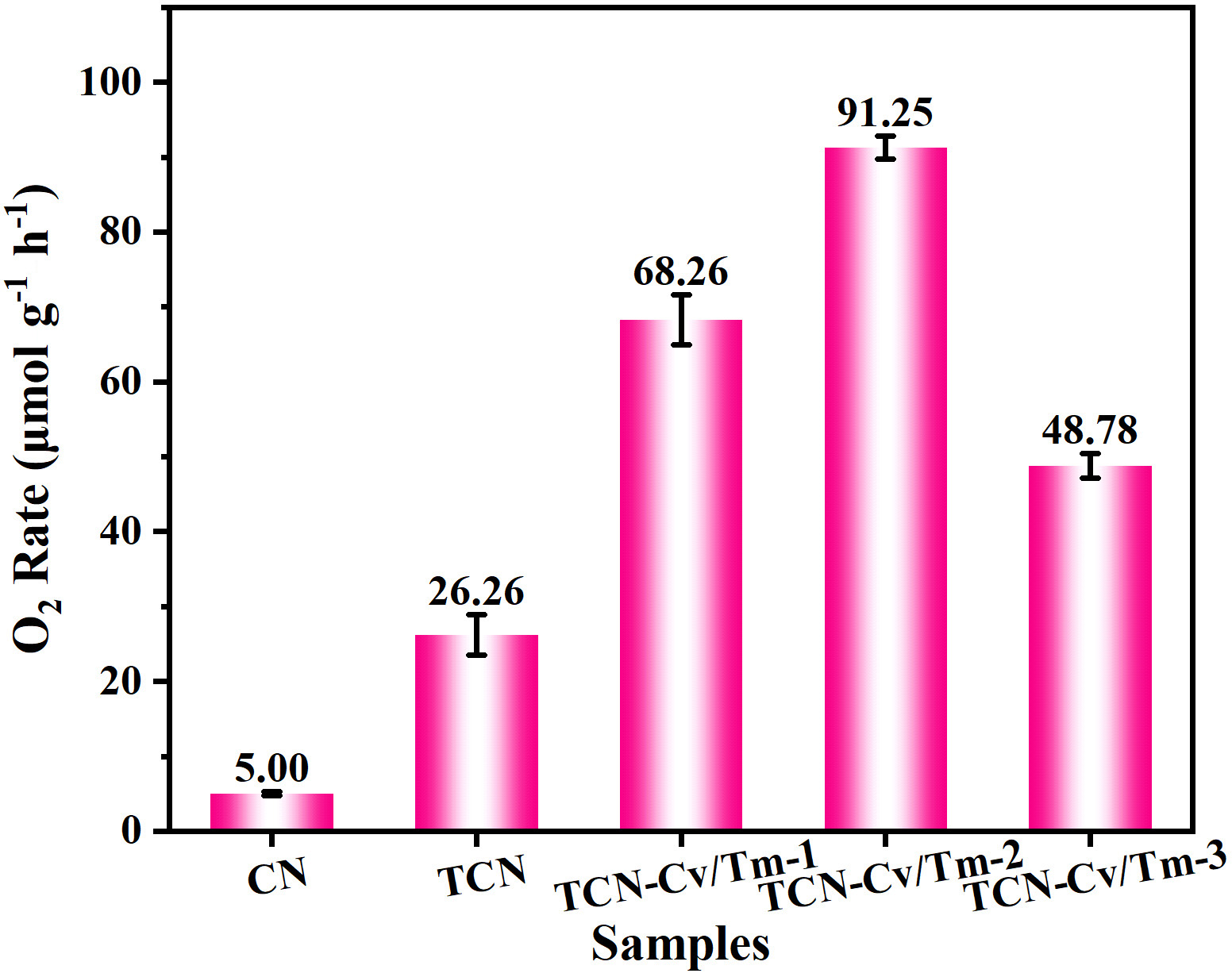
**

**Figure S16.** O_2_ production rate of CN, TCN, TCN-Cv/Tm-1, TCN-Cv/Tm-2, and TCN-Cv/Tm-3 samples during CO_2_ reduction.

***2.15. Table of AQE comparison data***

**Table S10.** Comparison with the AQE of other existing reported C_3_N_4_-based catalysts for photocatalytic CO_2_ reduction.

| **Catalysts** | **Wavelength (nm)** | **AQE (%)** | **Ref./Year** |
| --- | --- | --- | --- |
| Feqpy-BA/C_3_N_4_ | 460 | 0.10 | [57]/2022 |
| Coqpy@mpg-C_3_N_4_ | n/a | 0.25 | [58]/2020 |
| CNNA/rGO | 420 | 0.254 | [59]/2019 |
| RuRu’/Ag/NS-C_3_N_4_ | 400 | 0.20 | [60]/2017 |
| CISCN20 | 420 | 0.14 | [61]/2017 |
| ZnV_2_O_6_/pCN | 450 | 0.081 | [62]/2019 |
| Co^2+^@C_3_N_4_ | 400 | 0.40 | [63]/2018 |
| NCD/LDH/CN | 400 | 0.62 | [64]/2019 |
| Bi_3_O_4_Cl/20%g-C_3_N_4_ | 365 | 0.14 | [65]/2021 |
| Bi/g-C_3_N_4_ | 420 | 0.198 | [66]/2021 |
| MoS_2_/g-C_3_N_4_ | 420 | 0.255 | [67]/2018 |
| 1%Co-CN | 420 | 0.30 | [68]/2021 |
| TCN-Cv/Tm-2 | 385 | 0.84 | This work/- |

***2.16. Characterizations of TCN-Cv/Tm-2 after the cyclic stability test***

**
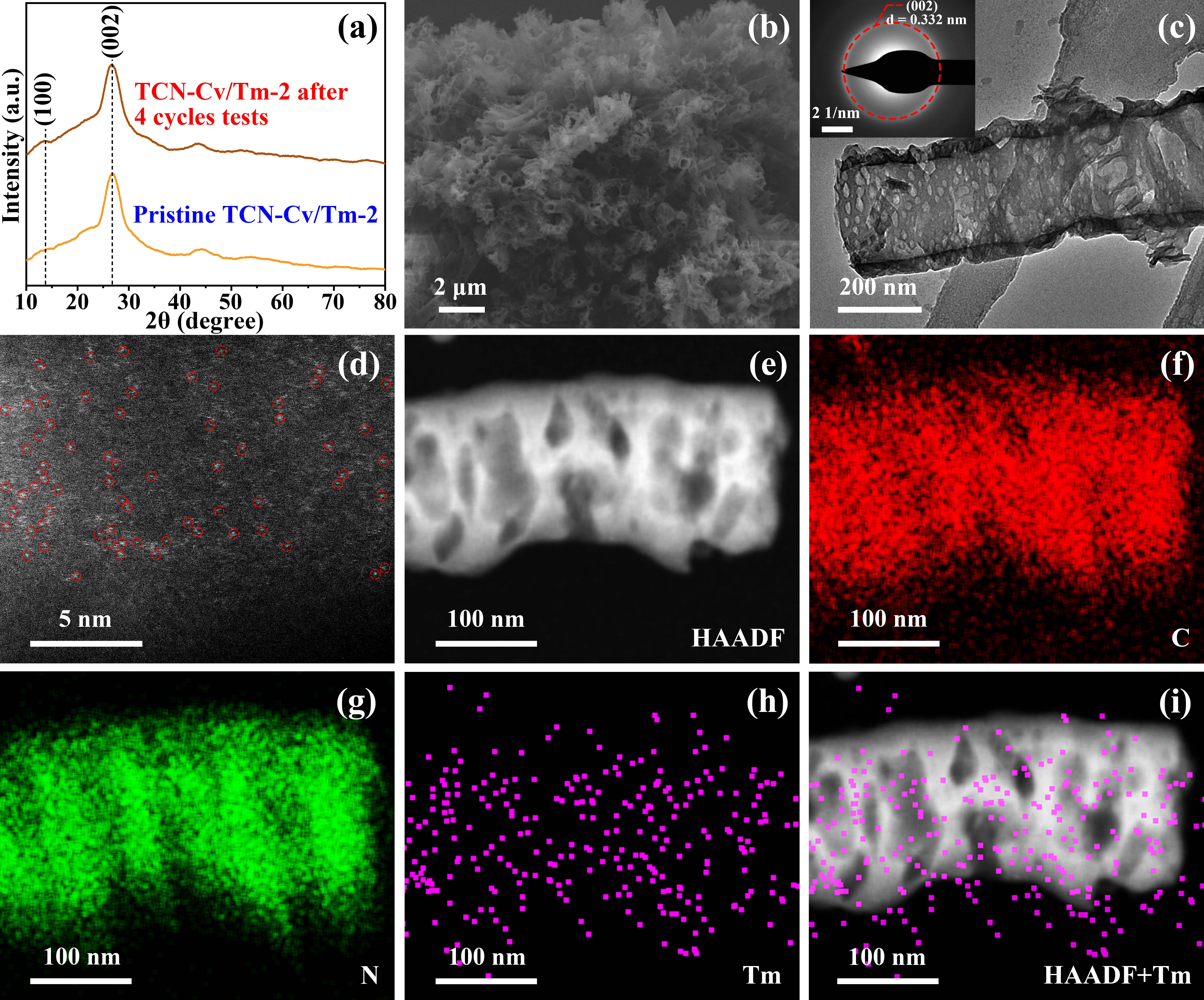
**

**Figure S17.** (a) XRD patterns of the pristine and used TCN-Cv/Tm-2 after four photocatalytic cycle tests. (b) SEM image, (c) TEM and SAED (inset) images, (d) AC HAADF-STEM image, and (e-i) EDS mapping images of the TCN-Cv/Tm-2 after four cycling tests.

**
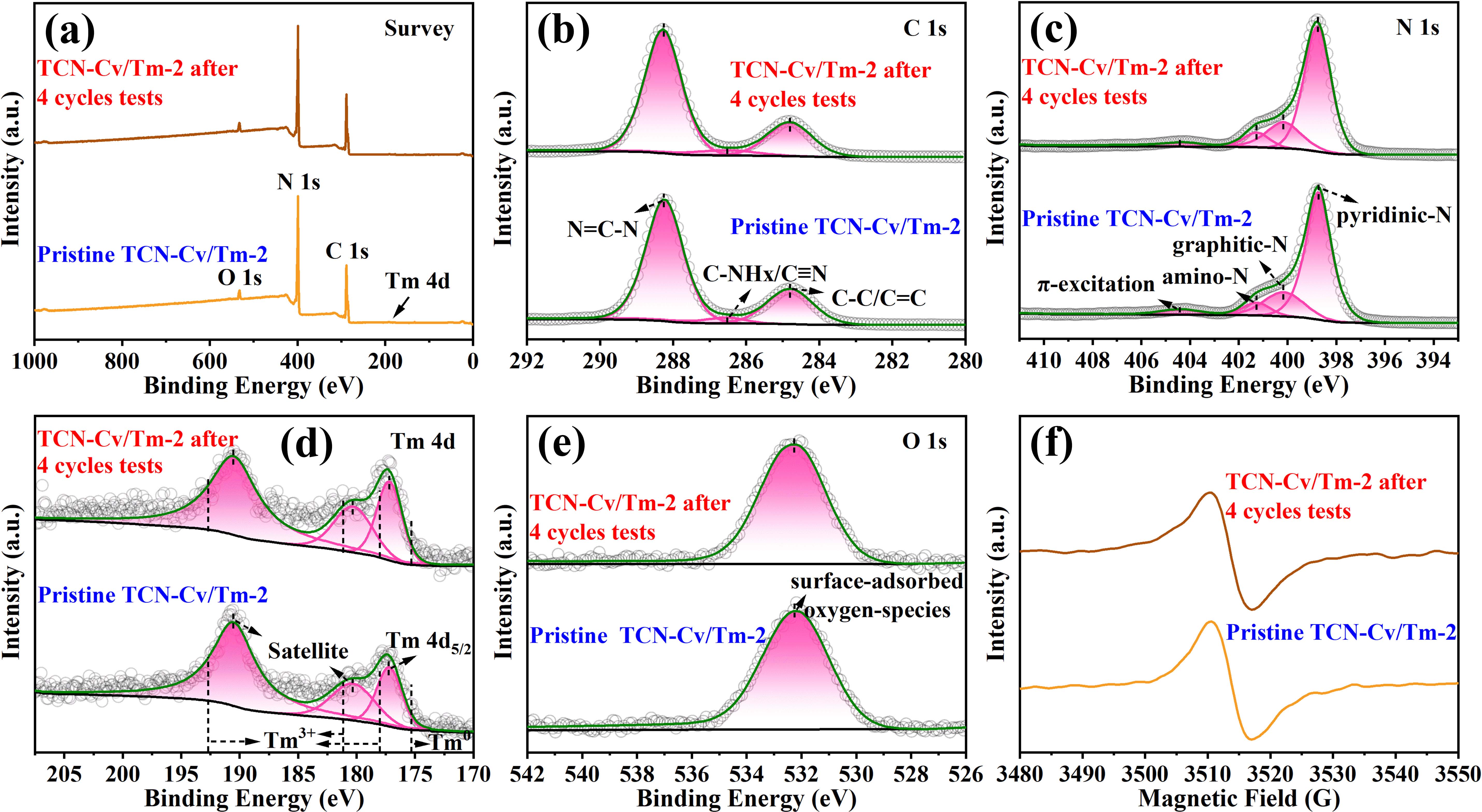
**

**Figure S18.** XPS and EPR spectra of the pristine and used TCN-Cv/Tm-2 after four photocatalytic cycle tests. (a) XPS survey spectra, (b) high-resolution C 1s XPS spectra, (c) high-resolution N 1s XPS spectra, (d) high-resolution Tm 4d XPS spectra, (e) high-resolution O 1s XPS spectra, and (f) EPR spectra.

***2.17. Band structure analysis***

**
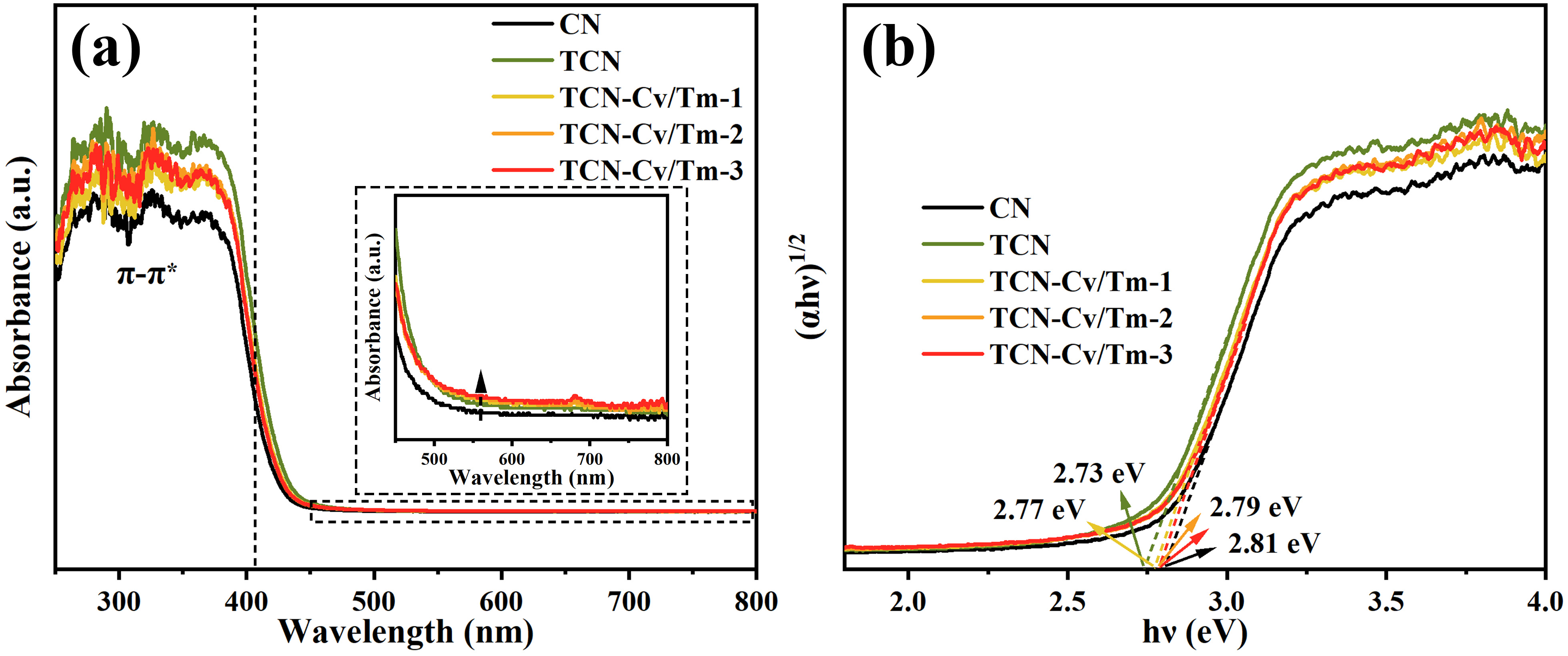
**

**Figure S19.** (a) UV-vis spectra (inset: an enlarged image of the partial region) and (b) Tauc-plots of the (αhν)^1/2^ versus hν for CN, TCN, and TCN-Cv/Tm-Y (Y = 1, 2, or 3) samples.

**
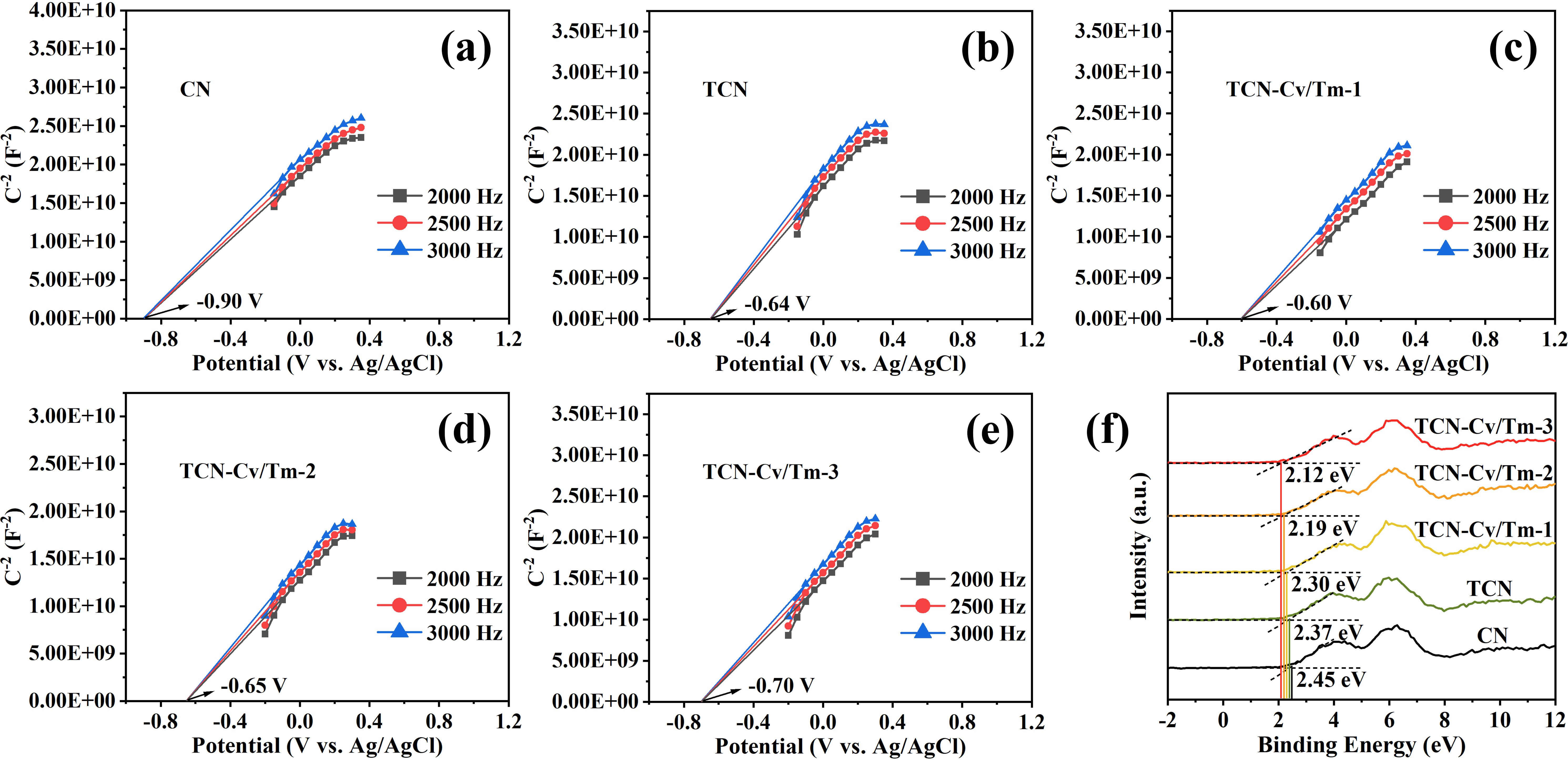
**

**Figure S20.** (a-e) Mott-Schottky measurements and (f) VB-XPS plots of CN, TCN, and TCN-Cv/Tm-Y (Y = 1, 2, or 3) samples.

**
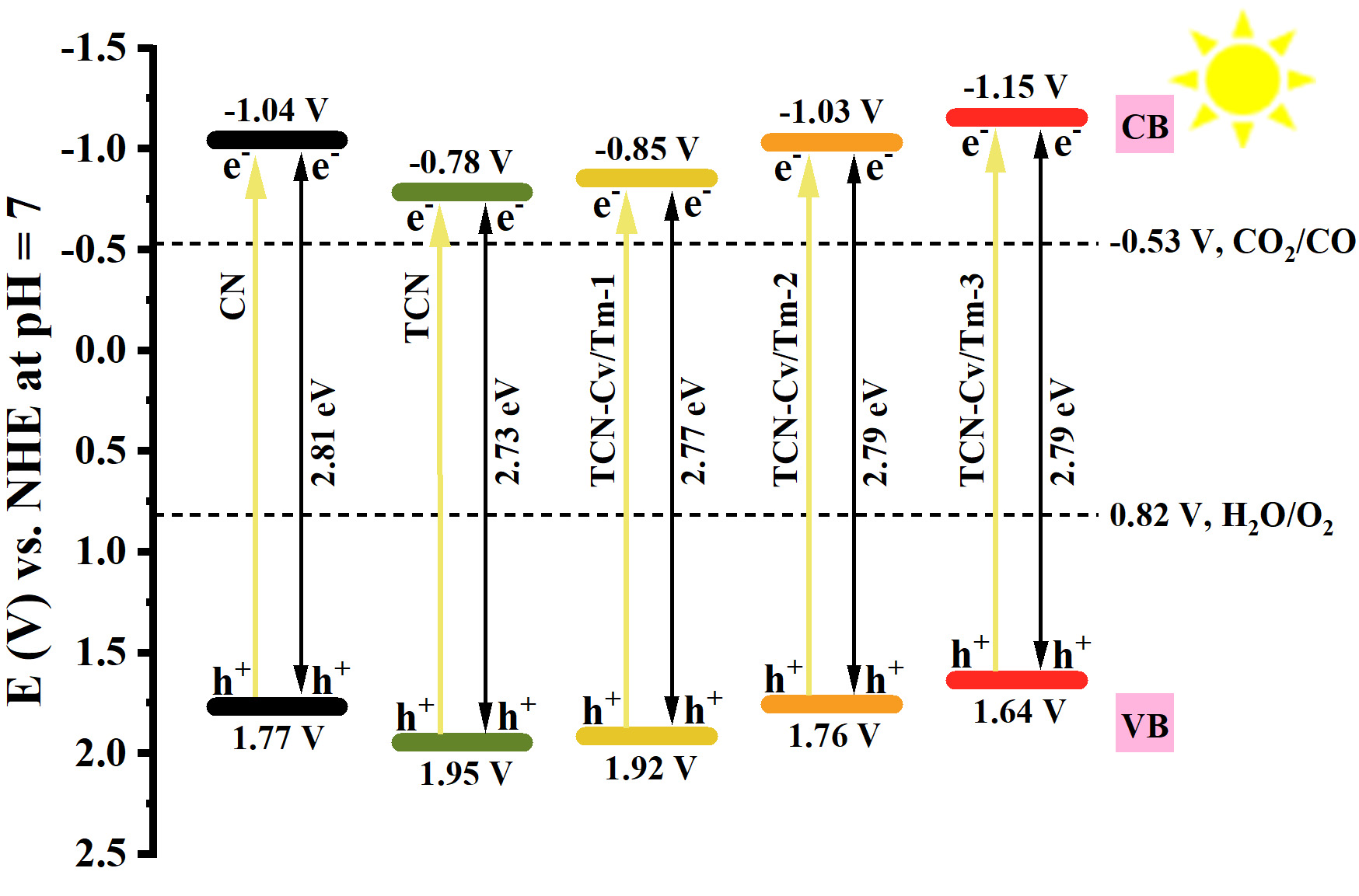
**

**Figure S21.** Energy band position diagram of CN, TCN, and TCN-Cv/Tm-Y (Y = 1, 2, or 3) samples.

**
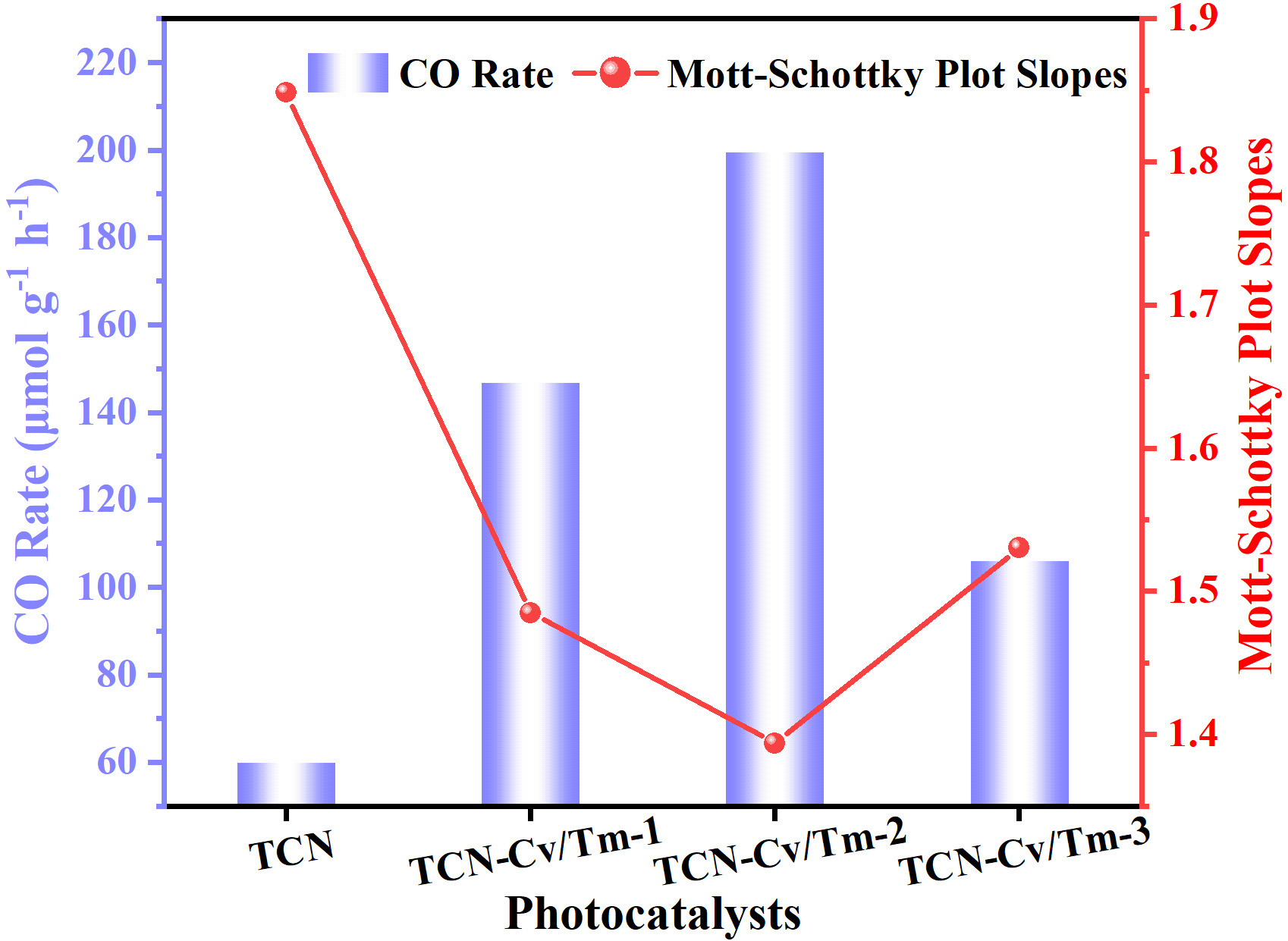
**

**Figure S22.** Relationship plot between the slope of the Mott-Schottky curve and photocatalytic CO_2_ reduction activity over TCN and TCN-Cv/Tm-Y (Y = 1, 2, or 3) samples.

***2.18. Photoelectrochemical analysis***

**
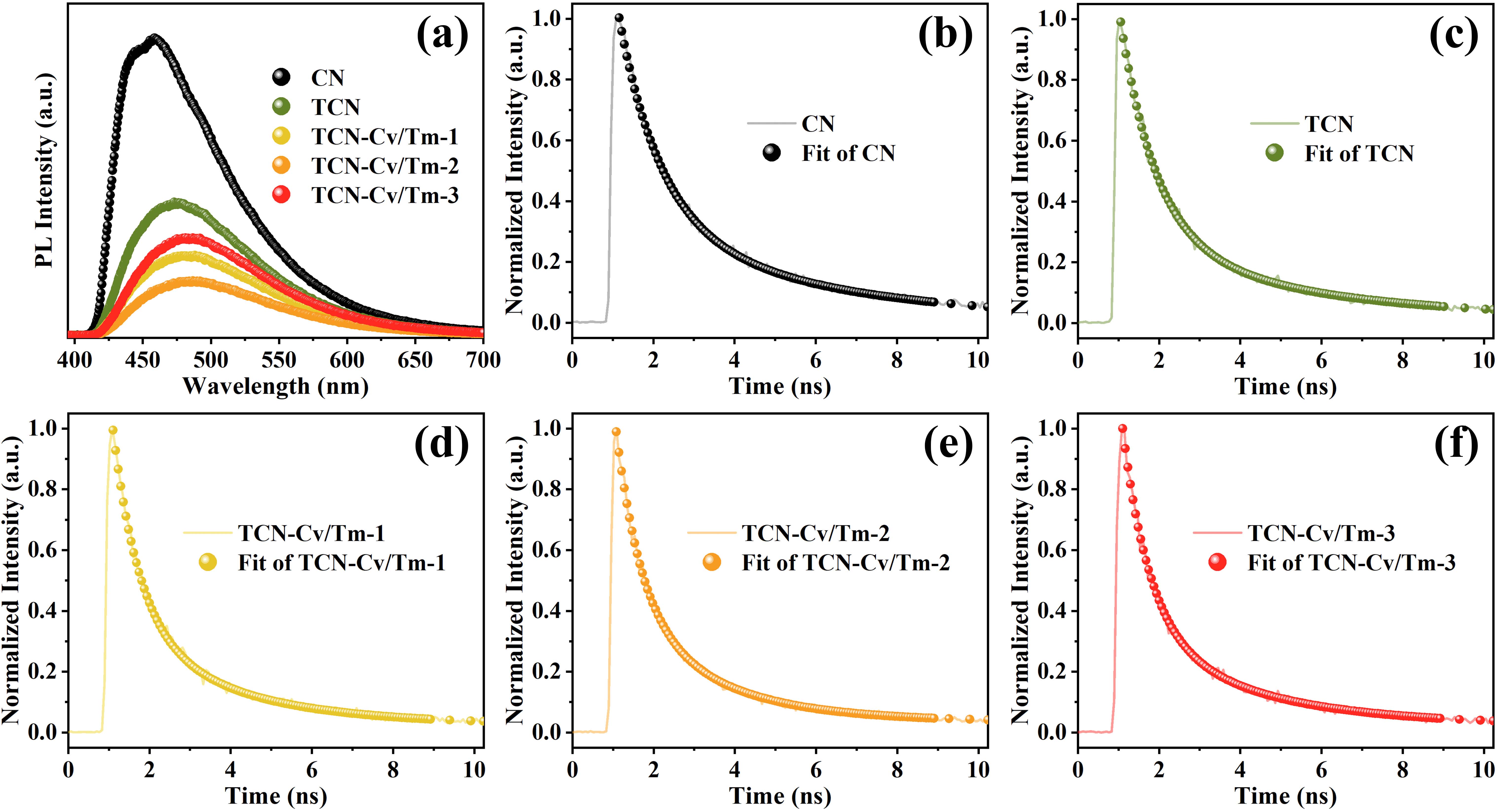
**

**Figure S23.** (a) The steady-state PL spectra and (b-f) the normalized TRF emission decay spectra of CN, TCN, and TCN-Cv/Tm-Y (Y = 1, 2, or 3) samples.

**Table S11.** The kinetics of PL decay parameters of CN, TCN, and TCN-Cv/Tm-Y (Y = 1, 2, or 3) samples were obtained by bi-exponential decay fitting.

| **Samples** | CN | TCN | TCN-Cv/Tm-1 | TCN-Cv/Tm-2 | TCN-Cv/Tm-3 |
| --- | --- | --- | --- | --- | --- |
| **A_1_** | 0.66555 | 0.78769 | 0.64432 | 0.64942 | 0.64633 |
| **τ_1_ (ns)** | 1.01822 | 0.86668 | 0.65387 | 0.60916 | 0.66644 |
| **A_2_** | 0.34049 | 0.28549 | 0.33774 | 0.40542 | 0.32980 |
| **τ_2_ (ns)** | 4.40822 | 3.89890 | 2.67212 | 2.22147 | 2.91511 |
| **τ_ave_ (ns)** | 3.35374 | 2.74618 | 2.02980 | 1.72940 | 2.21935 |
| **K_et_ (10^8^ s^-1^)** | - | - | 1.28974 | 2.14398 | 0.86814 |

Note:

τ_ave_ = $\frac{\text{A}_{\text{1}}\text{τ}_{\text{1}}^{\text{2}} \text{+} \text{A}_{\text{2}}\text{τ}_{\text{2}}^{\text{2}}}{\text{A}_{\text{1}}\text{τ}_{\text{1}} \text{+} \text{A}_{\text{2}}\text{τ}_{\text{2}}}$;

K_et(TCN→TCN-Cv/Tm-Y)_ = 1/τ_ave(TCN-Cv/Tm-Y)_ - 1/τ_ave(TCN)_,

Where, τ_1_, τ_2_, and τ_ave_ are photogenerated charge lifetime, and K_et_ is electron transfer rate constant, respectively.

**
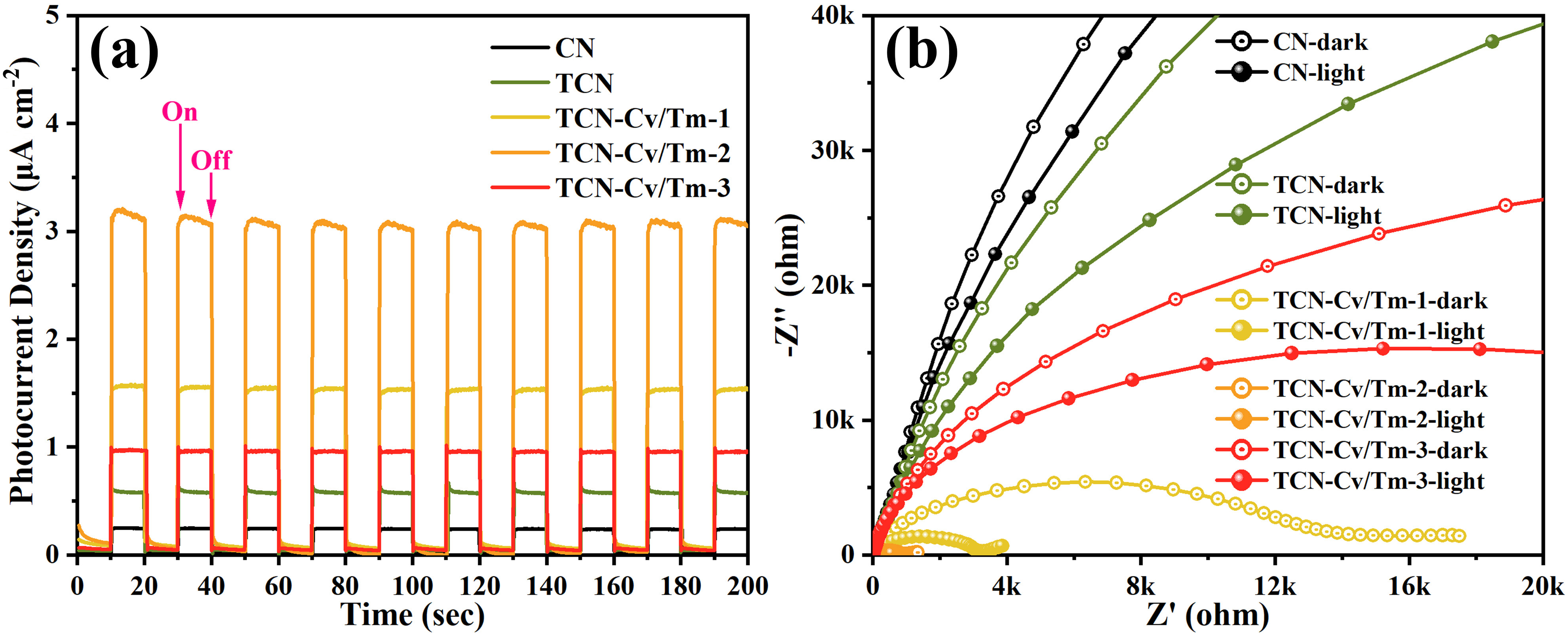
**

**Figure S24.** (a) Photocurrent density curves and (b) EIS spectra with the absence and presence of light illumination for CN, TCN, and TCN-Cv/Tm-Y (Y = 1, 2, or 3) samples.

***2.19. Analysis of specific surface area and pore structure***

**
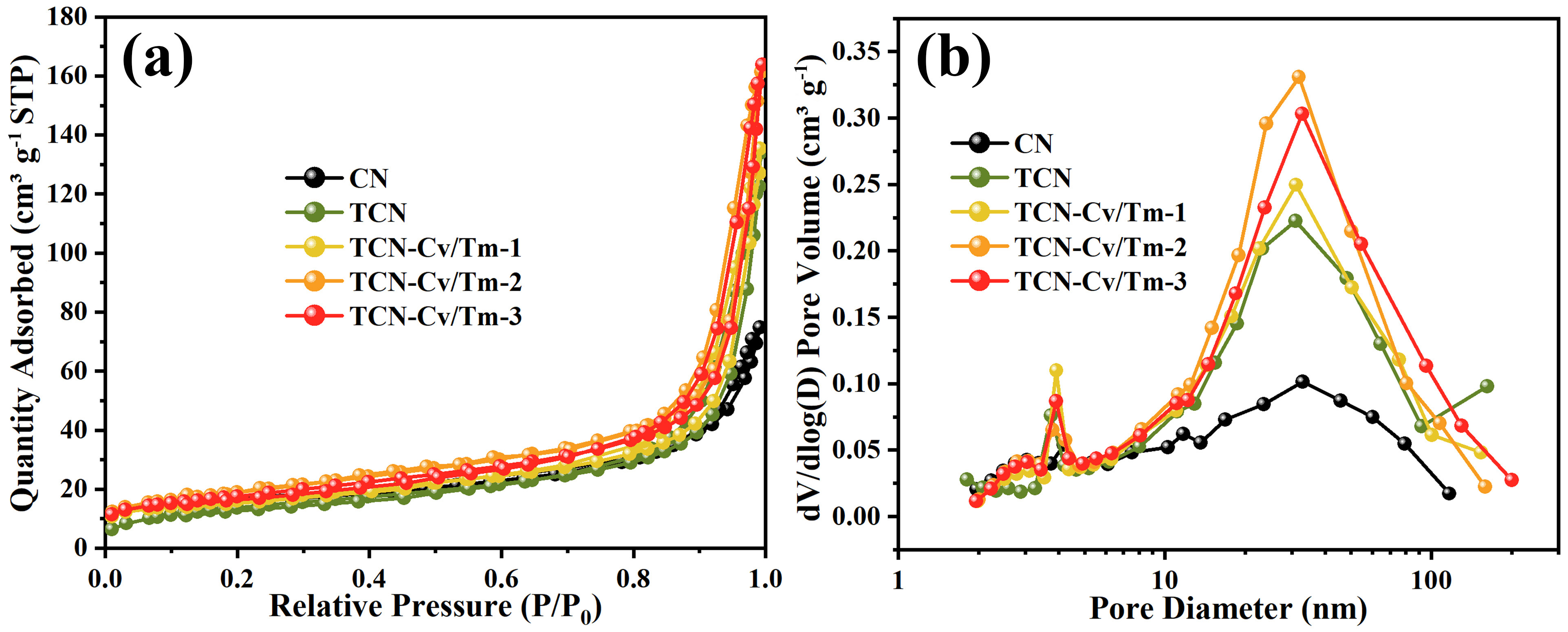
**

**Figure S25.** The nitrogen adsorption-desorption isotherms at 77 K (a) and pore size distribution (b) of CN, TCN, and TCN-Cv/Tm-Y (Y = 1, 2, or 3) samples.

**Table S12.** The specific surface areas and pore structure parameters of CN, TCN, and TCN-Cv/Tm-Y (Y = 1, 2, or 3) samples.

| **Samples** | **S_BET_ (m^2^/g)^a^** | **Pore volume (cm^3^/g)^b^** | **Pore size (nm)^c^** |
| --- | --- | --- | --- |
| CN | 8.88 | 0.11 | 10.95 |
| TCN | 51.08 | 0.21 | 16.52 |
| TCN-Cv/Tm-1 | 57.01 | 0.21 | 16.05 |
| TCN-Cv/Tm-2 | 67.35 | 0.25 | 16.83 |
| TCN-Cv/Tm-3 | 62.52 | 0.26 | 17.71 |

Note:

^a^ The S_BET_ refers to BET surface area; ^b^ The pore volume is BJH desorption cumulative volume of pores between 17.000 Å and 3000.000 Å diameter; ^c^ The pore size is BJH desorption average pore diameter (4V/A).

***2.20. CO_2_ adsorption isotherms analysis***

**
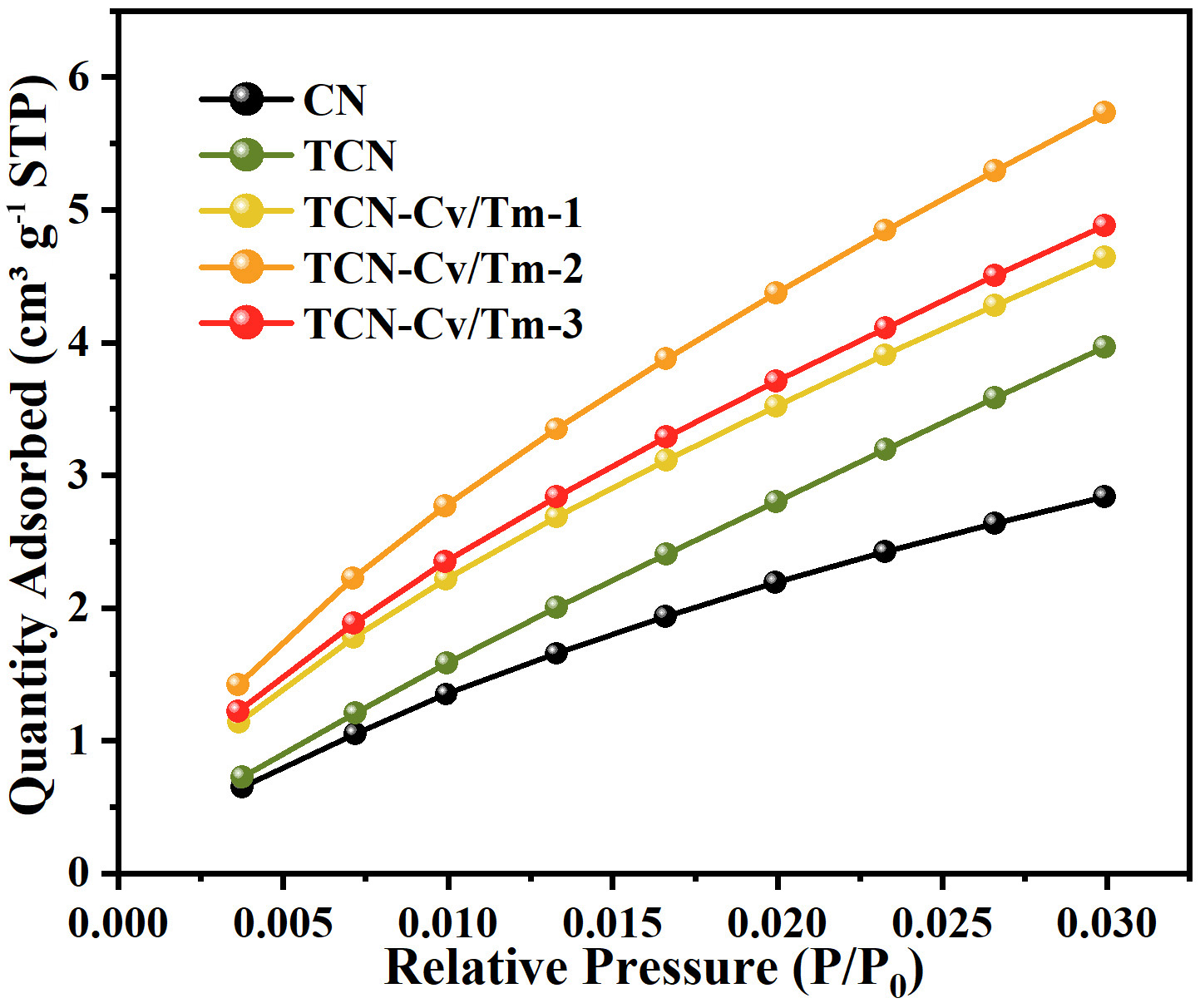
**

**Figure S26.** CO_2_ adsorption isotherms of CN, TCN, and TCN-Cv/Tm-Y (Y = 1, 2, or 3) samples.

***2.21. ISFTIR analysis***

**
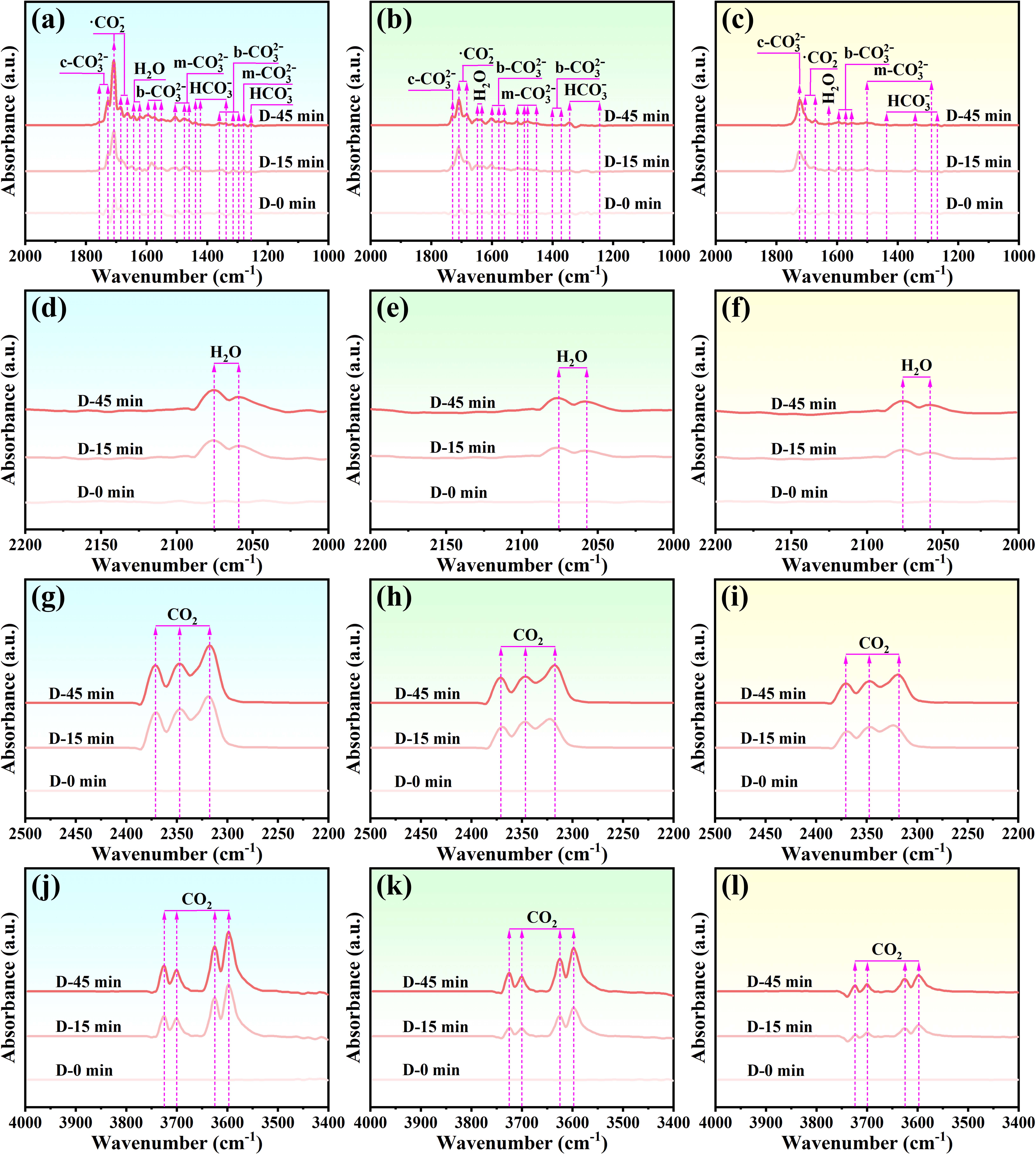
**

**Figure S27.** The ISFTIR spectra of CO_2_ and H_2_O interaction with TCN-Cv/Tm-2 (a, d, g, j), TCN (b, e, h, k), or CN (c, f, i, l) in the dark (D refers to dark environment, L refers to light environment).

***2.22. Calculation simulation analysis***

**
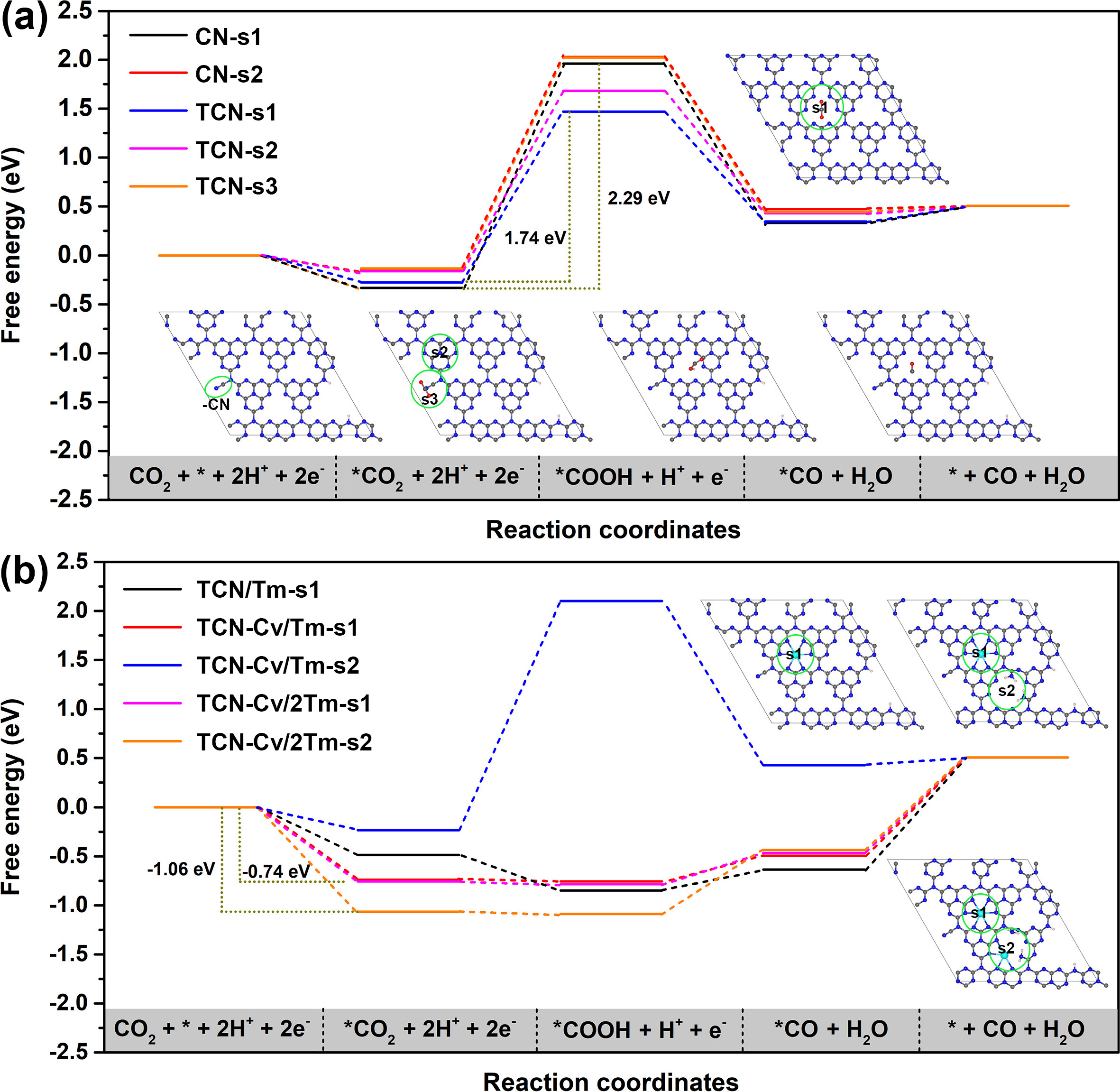
**

**Figure S28.** (a) The calculated free energy diagram to the reaction path followed by the CO2 conversion on bare CN and TCN in different reaction sites. (b) The calculated free energy diagram on TCN/Tm, TCN-Cv/Tm, and TCN-Cv/2Tm in target sites.


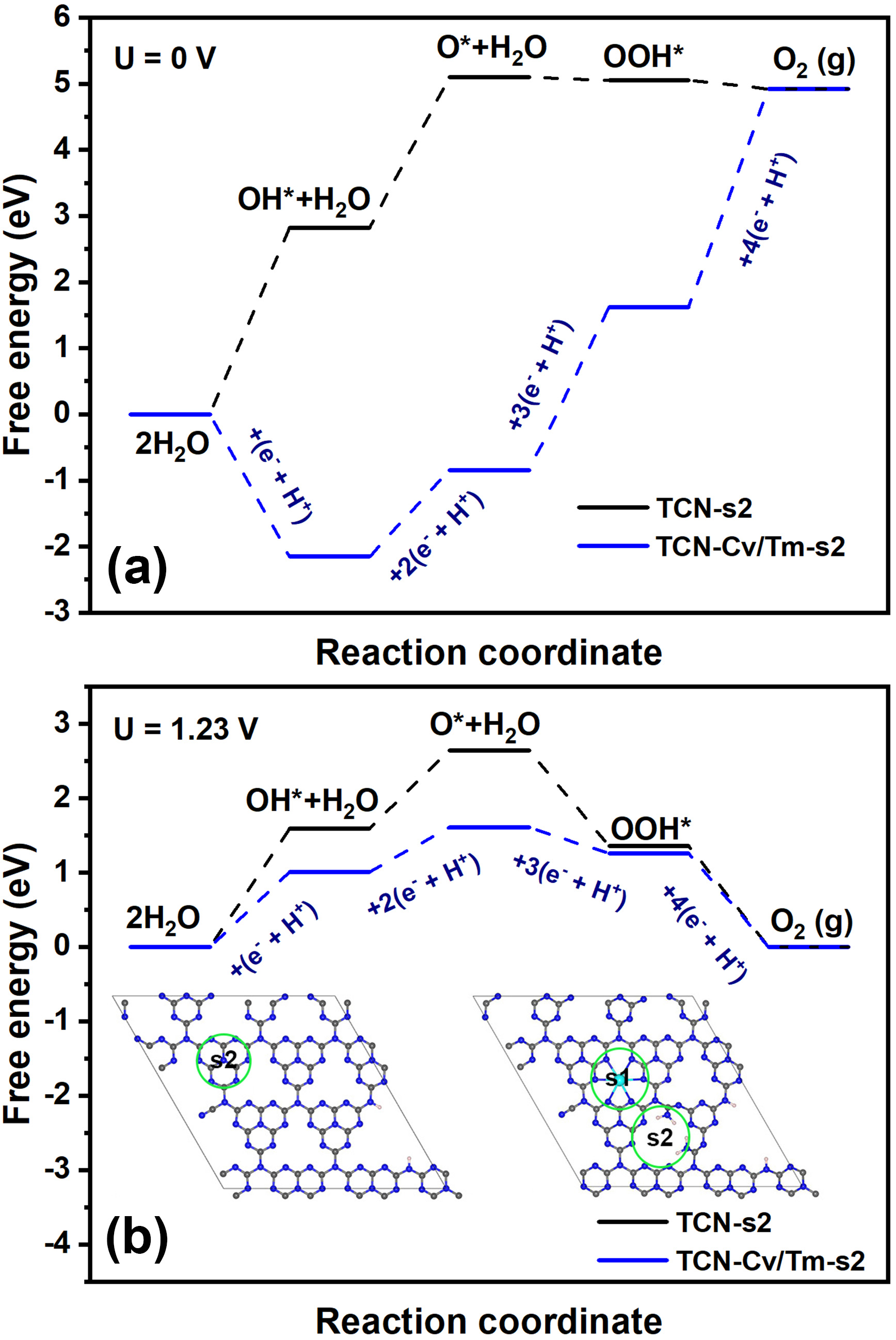


**Figure S29.** The calculated free energy diagram (a) U = 0 V and (b) U = 1.23 V of the H_2_O oxidation reaction path in target sites.

**Table S13.** DFT models, calculated CO_2_ and *COOH adsorption energies (E_ads_) on the different sites.

| **Sites** | **E_ads_(CO_2_) (eV)** | **E_ads_(*COOH) (eV)** | **DFT models** |
| --- | --- | --- | --- |
| CN-s1 | -0.235 | -0.202 | 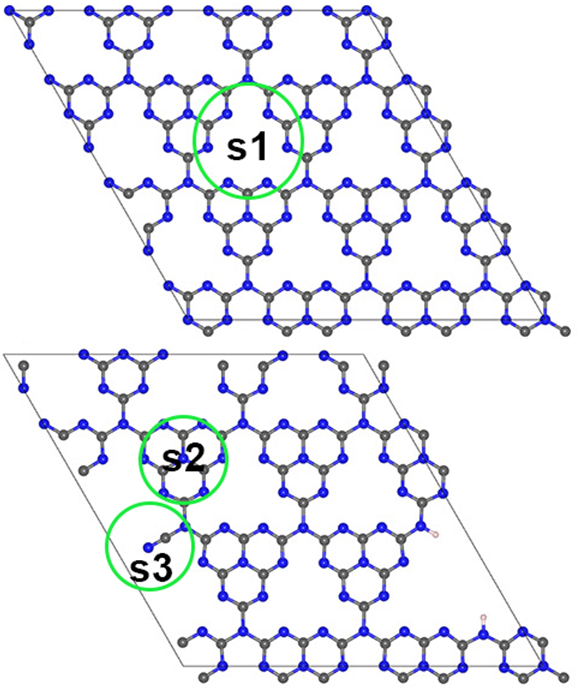 |
| CN-s2 | -0.108 | -0.133 |  |
| TCN-s1 | -0.229 | -0.693 |  |
| TCN-s2 | -0.097 | -0.513 |  |
| TCN-s3 | -0.102 | -0.136 |  |
| TCN/Tm-s1 | -0.365 | -3.007 | 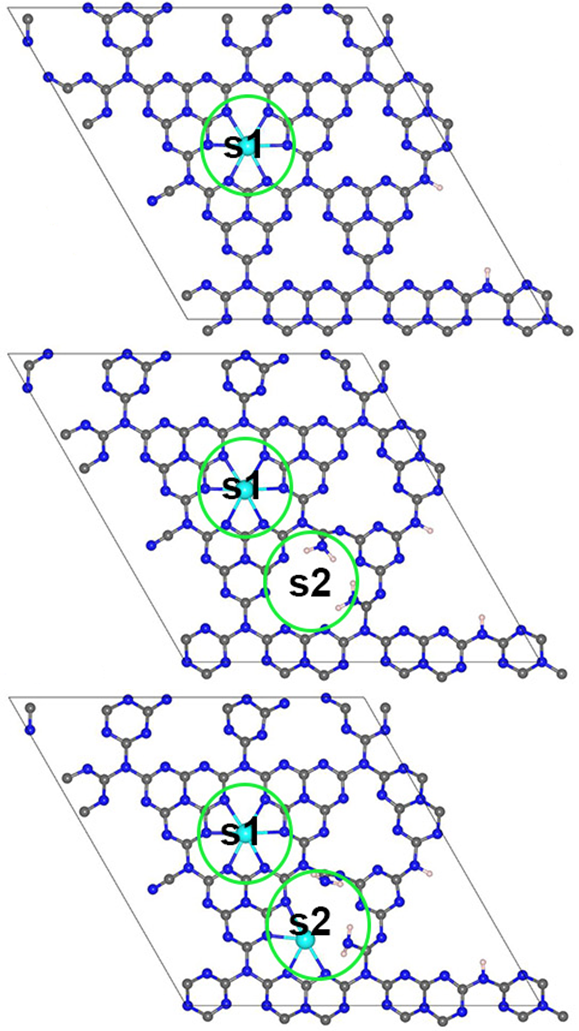 |
| TCN-Cv/Tm-s1 | -0.615 | -2.903 |  |
| TCN-Cv/Tm-s2 | -0.135 | 0.010 |  |
| TCN-Cv/2Tm-s1 | -0.642 | -2.933 |  |
| TCN-Cv/2Tm-s2 | -0.967 | -3.281 |  |

***2.23. Hydrophilicity/hydrophobicity analysis***


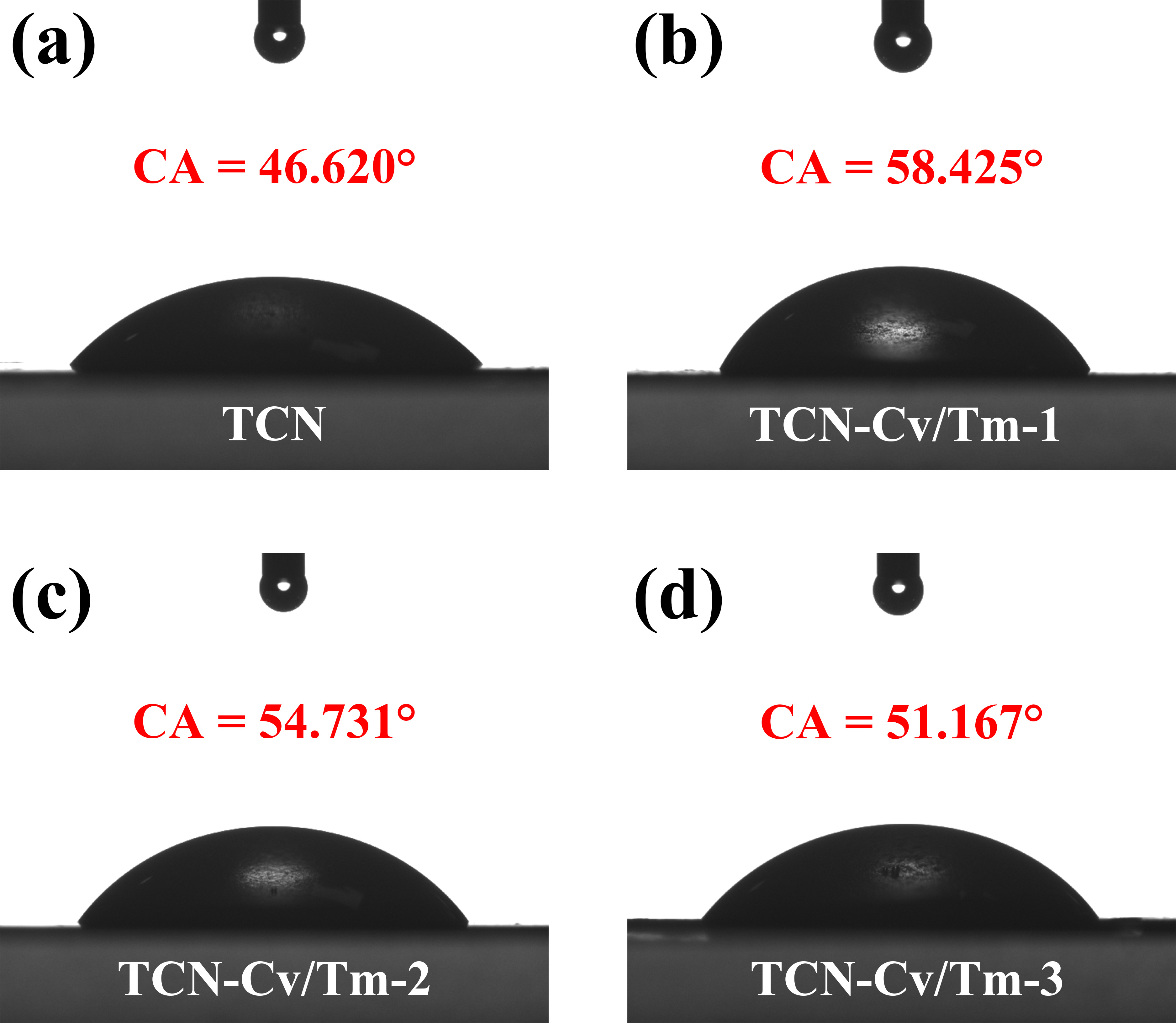


**Figure S30.** Digital images of contact angle (CA) tests for as-prepared samples ((a) TCN, (b) TCN-Cv/Tm-1, (c) TCN-Cv/Tm-2, and (d) TCN-Cv/Tm-3).

**Additional discussions:** As shown in Figure S30, pure TCN exhibits hydrophilicity (contact angle (CA) of 46.620°), which should be derived from the presence of edge NHx (x = 1, 2) and CN groups.^[69]^ After introducing Tm single atoms, also due to the presence of the aforementioned hydrophilic groups, TCN-Cv/Tm-Y still shows hydrophilicity with CAs of 58.425° (TCN-Cv/Tm-1), 54.731° (TCN-Cv/Tm-2), and 51.167° (TCN-Cv/Tm-3), respectively. However, the hydrophobicity of TCN-Cv/Tm-Y is significantly enhanced compared to pure TCN, which is mainly attributed to the hydrophobic property of Tm single atoms.^[70]^ In addition, the hydrophilicity of the synthetic material gradually increases again as the Tm content continues to increase, which should be attributed to the significant increase in NHx content caused by the formation of C vacancies. This phenomenon also provides indirect evidence of the generation of C vacancies. In conclusion, the hydrophobicity of TCN-Cv/Tm-Y is significantly stronger than that of TCN, which explains to some extent that the Tm single-atom sites are the CO_2_ adsorption and activation centers.

***2.24. Table of machine learning (ML) parameters***

**Table S14.** List of elemental features used as descriptors and DFT-calculated CO_2_ activation energy barriers (Delta G). Period is the period number of a metal element; Group is the group number of a metal element; RWIGS is the bulk wigner-seitz radius of a metal element (Å); Nve is the valence electron number of a metal element; Am is the electron affinity of a metal element (kJ/mol); Mm is the atomic mass of a metal element (u); Xm is the electronegativity of a metal element; Dm is the density of a metal element (g/cm^3^).

| Metal | Period | Group | RWIGS | Nve | Am | Mm | Xm | Dm | Delta G |
| --- | --- | --- | --- | --- | --- | --- | --- | --- | --- |
| Mg | 3 | 2 | 2.88 | 2 | -40 | 24.03 | 1.31 | 1.74 | -0.438 |
| Al | 3 | 13 | 2.65 | 3 | 41.76 | 26.98 | 1.61 | 2.7 | -0.106 |
| Si | 3 | 14 | 2.48 | 4 | 134.06 | 28.08 | 1.9 | 2.33 | -0.189 |
| Ti | 4 | 4 | 2.5 | 4 | 7.28 | 47.87 | 1.54 | 4.51 | -0.685 |
| Mn | 4 | 7 | 2.5 | 7 | -50 | 54.94 | 1.55 | 7.21 | -0.483 |
| Fe | 4 | 8 | 2.46 | 8 | 14.78 | 55.85 | 1.83 | 7.87 | -0.306 |
| Co | 4 | 9 | 2.46 | 9 | 63.89 | 58.93 | 1.88 | 8.86 | -0.308 |
| Ni | 4 | 10 | 2.43 | 10 | 111.65 | 58.69 | 1.91 | 8.9 | -0.278 |
| Cu | 4 | 11 | 2.2 | 11 | 119.23 | 63.55 | 1.9 | 8.96 | -0.284 |
| Zn | 4 | 12 | 2.4 | 12 | -58 | 65.38 | 1.65 | 7.14 | -0.323 |
| Ge | 4 | 14 | 2.3 | 4 | 118.93 | 72.63 | 2.01 | 5.32 | -0.144 |
| Mo | 5 | 6 | 2.75 | 6 | 72.1 | 95.96 | 2.16 | 10.28 | -0.538 |
| Tc | 5 | 7 | 2.75 | 7 | 53 | 98.91 | 1.9 | 11.5 | -0.589 |
| Ru | 5 | 8 | 2.65 | 8 | 100.27 | 101.1 | 2.2 | 12.45 | -0.495 |
| Rh | 5 | 9 | 2.65 | 9 | 100.27 | 102.9 | 2.28 | 12.41 | -0.411 |
| Pd | 5 | 10 | 2.71 | 10 | 54.24 | 106.4 | 2.2 | 12.02 | -0.382 |
| In | 5 | 13 | 3.17 | 3 | 28.9 | 114.82 | 1.78 | 7.31 | -0.171 |
| Sn | 5 | 14 | 2.96 | 4 | 107.29 | 118.7 | 1.96 | 7.26 | -0.098 |
| Ce | 6 | 3 | 2.5 | 12 | 55 | 140.12 | 1.12 | 6.689 | -0.735 |
| Tm | 6 | 3 | 3 | 23 | 99 | 168.93 | 1.25 | 9.32 | -0.740 |
| Hf | 6 | 4 | 3.05 | 4 | 17.18 | 178.5 | 1.3 | 13.31 | -1.118 |
| W | 6 | 6 | 2.75 | 6 | 78.76 | 183.8 | 2.36 | 19.3 | -0.767 |
| Ir | 6 | 9 | 2.84 | 9 | 150.94 | 192.2 | 2.2 | 22.56 | -0.374 |
| Pt | 6 | 10 | 2.75 | 10 | 205.04 | 195.1 | 2.28 | 21.45 | -0.376 |
| Ta | 6 | 5 | 2.84 | 5 | 31 | 180.95 | 1.5 | 16.65 | -1.067 |
| Zr | 5 | 4 | 3.07 | 12 | 41.1 | 91.22 | 1.33 | 6.511 | -1.122 |
| Er | 6 | 3 | 3 | 22 | 50 | 167.26 | 1.24 | 9.066 | -0.743 |
| Eu | 6 | 3 | 2.8 | 17 | 50 | 151.965 | 1.2 | 5.224 | -0.612 |
| La | 6 | 3 | 2.8 | 11 | 48 | 138.9 | 1.1 | 6.146 | -0.511 |

***2.25. Schematic diagram of reaction mechanism***

**
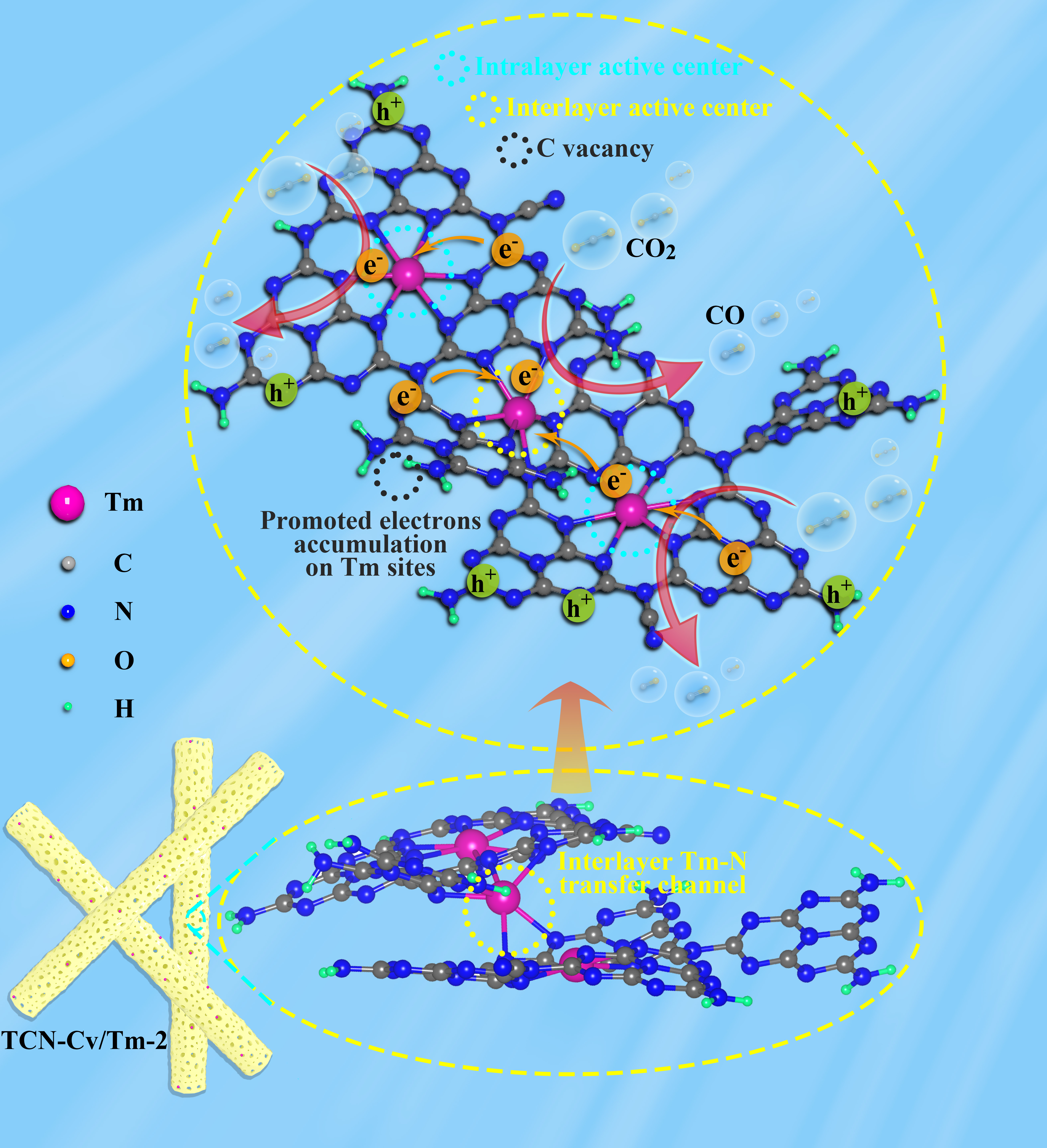
**

**Figure S31.** The reaction mechanism for photocatalytic conversion of CO_2_ to CO over the synthetic synergetic catalyst.

**References**

[1] B. Ravel, M. Newville, *J. Synchrotron Radiat.* **2005**, *12*, 537.

[2] a) H. Funke, A. C. Scheinost, M. Chukalina, *Phys. Rev. B* **2005**, *71*, 094110; b) H. Funke, M. Chukalina, A. C. Scheinost, *J. Synchrotron. Radiat.* **2007**, *14*, 426.

[3] Q. Han, X. Bai, Z. Man, H. He, L. Li, J. Hu, A. Alsaedi, T. Hayat, Z. Yu, W. Zhang, J. Wang, Y. Zhou, Z. Zou, *J. Am. Chem. Soc.* **2019**, *141*, 4209.

[4] a) G. Kresse, J. Furthmüller, *Comput. Mater. Sci.* **1996**, *6*, 15; b) G. Kresse, J. Furthmüller, *Phys. Rev. B* **1996**, *54*, 11169.

[5] a) P. E. Blöchl, *Phys. Rev. B* **1994**, *50*, 17953; b) G. Kresse, D. Joubert, *Phys. Rev. B* **1999**, *59*, 1758.

[6] J. P. Perdew, K. Burke, M. Erzenhof, *Phys. Rev. Lett.* **1996**, *77*, 3865.

[7] S. Grimme, S. Ehrlich, L. Goerigk, *J. Comput. Chem.* **2011**, *32*, 1456.

[8] J. K. Nørskov, J. Rossmeisl, A. Logadottir, L. Lindqvist, *J. Phys. Chem. B* **2004**, *108*, 17886.

[9] Y. Wang, Y. Qu, B. Qu, L. Bai, Y. Liu, Z. D. Yang, W. Zhang, L. Jing, H. Fu, *Adv. Mater.* **2021**, *33*, 2105482.

[10] Y. Li, B. Li, D. Zhang, L. Cheng, Q. Xiang, *ACS Nano* **2020**, *14*, 10552.

[11] G. Jia, Z. Wang, M. Gong, Y. Wang, L. H. Li, Y. Dong, L. Liu, L. Zhang, J. Zhao, W. Zheng, X. Cui, *Carbon Energy* **2022**, *5*, e270.

[12] Y. Zhao, Z. Han, G. Gao, W. Zhang, Y. Qu, H. Zhu, P. Zhu, G. Wang, *Adv. Funct. Mater.* **2021**, *31*, 2104976.

[13] X. Sun, L. Sun, G. Li, Y. Tuo, C. Ye, J. Yang, J. Low, X. Yu, J. H. Bitter, Y. Lei, D. Wang, Y. Li, *Angew. Chem. Int. Ed.* **2022**, *61*, e202207677.

[14] Y. Duan, Y. Wang, W. Zhang, J. Zhang, C. Ban, D. Yu, K. Zhou, J. Tang, X. Zhang, X. Han, L. Gan, X. Tao, X. Zhou, *Adv. Funct. Mater.* **2023**, *33*, 2301729.

[15] X. Shi, Y. Huang, Y. Bo, D. Duan, Z. Wang, J. Cao, G. Zhu, W. Ho, L. Wang, T. Huang, Y. Xiong, *Angew. Chem. Int. Ed.* **2022**, *61*, e202203063.

[16] P. Chen, B. Lei, X. Dong, H. Wang, J. Sheng, W. Cui, J. Li, Y. Sun, Z. Wang, F. Dong, *ACS Nano* **2020**, *14*, 15841.

[17] S. Ji, Y. Qu, T. Wang, Y. Chen, G. Wang, X. Li, J. Dong, Q. Y. Chen, W. Zhang, Z. Zhang, S. Liang, R. Yu, Y. Wang, D. Wang, Y. Li, *Angew. Chem. Int. Ed.* **2020**, *59*, 10651.

[18] Z. Zhao, W. Liu, Y. Shi, H. Zhang, X. Song, W. Shang, C. Hao, *Phys. Chem. Chem. Phys.* **2021**, *23*, 4690.

[19] J. Wang, T. Heil, B. Zhu, C. W. Tung, J. Yu, H. M. Chen, M. Antonietti, S. Cao, *ACS Nano* **2020**, *14*, 8584.

[20] R. Tang, H. Wang, X. Dong, S. Zhang, L. Zhang, F. Dong, *J. Colloid Interface Sci.* **2023**, *630*, 290.

[21] L. Cheng, H. Yin, C. Cai, J. Fan, Q. Xiang, *Small* **2020**, *16*, 2002411.

[22] H. Ou, S. Ning, P. Zhu, S. Chen, A. Han, Q. Kang, Z. Hu, J. Ye, D. Wang, Y. Li, *Angew. Chem. Int. Ed.* **2022**, *61*, e202206579.

[23] L. Cheng, X. Yue, L. Wang, D. Zhang, P. Zhang, J. Fan, Q. Xiang, *Adv. Mater.* **2021**, *33*, 2105135.

[24] L. Cheng, P. Zhang, Q. Wen, J. Fan, Q. Xiang, *Chin. J. Catal.* **2022**, *43*, 451.

[25] Y. Yang, F. Li, J. Chen, J. Fan, Q. Xiang, *ChemSusChem* **2020**, *13*, 1979.

[26] S. Hu, P. Qiao, X. Yi, Y. Lei, H. Hu, J. Ye, D. Wang, *Angew. Chem. Int. Ed.* **2023**, *62*, e202304585.

[27] Z. Wang, M. Zhang, J. Hu, Z. Li, W. Zhang, J. Zhang, Z. Wang, X. Guo, C. Yan, H. Yuan, M. Li, Y. Li, X. Sun, Z. Xu, S.-P. Feng, Z. Lu, *J. Power Sources* **2023**, *577*, 233188.

[28] X. Shi, Y. Huang, G. Zhu, W. Peng, M. Chen, *Nano Res.* **2023**, https://doi.org/10.1007/s12274-023-6079-y.

[29] J. Yang, K. Yang, X. Zhu, Z. Wang, Z. Yang, X. Ding, K. Zhong, M. He, H. Li, H. Xu, *Chem. Eng. J.* **2023**, *461*, 141841.

[30] S. Li, Y. Yang, S. Wan, R. Wang, M. Yu, F. Song, Q. Zhong, *J. Colloid Interface Sci.* **2023**, *651*, 726.

[31] J. Wang, Z. Jiang, *Ultrason. Sonochem.* **2023**, *92*, 106273.

[32] Q. Ye, Y. Zhou, Y. Xu, Q. Zhang, X. Shi, D. Li, D. Tian, D. Jiang, *Chem. Eng. J.* **2023**, *463*, 142395.

[33] Z. Xu, Y. Chen, B. Wang, Y. Ran, J. Zhong, M. Li, *J. Colloid Interface Sci.* **2023**, *651*, 645.

[34] H. A. E. Omr, R. Putikam, S.-P. Feng, M.-C. Lin, H. Lee, *Appl. Catal. B* **2023**, *339*, 123103.

[35] C. Cheng, L. Mao, X. Kang, C.-L. Dong, Y.-C. Huang, S. Shen, J. Shi, L. Guo, *Appl. Catal. B* **2023**, *331*, 122733.

[36] Z. Liu, J. Liang, Q. Song, Y. Li, Z. Zhang, M. Zhou, W. Wei, H. Xu, C.-S. Lee, H. Li, Z. Jiang, *Appl. Catal. B* **2023**, *328*, 122472.

[37] J. Tang, X. Li, Y. Ma, K. Wang, Z. Liu, Q. Zhang, *Appl. Catal. B* **2023**, *327*, 122417.

[38] S. Cao, H. Liu, Z. Jia, M. Guo, W. Gao, Z. Ding, W. Yang, L. Chen, W. Wang, *Chem. Eng. J.* **2023**, *455*, 140746.

[39] A. Deng, E. Zhao, Q. Li, Y. Sun, Y. Liu, S. Yang, H. He, Y. Xu, W. Zhao, H. Song, Z. Xu, Z. Chen, *ACS Nano* **2023**, *17*, 11869.

[40] A. Ziarati, J. Zhao, J. Afshani, R. Kazan, A. Perez Mellor, A. Rosspeintner, S. McKeown, T. Bürgi, *Small* **2023**, *19*, 2207857.

[41] B. Hu, M. Xiao, C. Liu, G. Che, J. Jia, L. Yan, H. Dong, *Sep. Purif. Technol.* **2023**, *315*, 123726.

[42] X. Zhang, K. Matras-Postolek, P. Yang, S. Ping Jiang, *J. Colloid Interface Sci.* **2023**, *636*, 646.

[43] Q. Ye, R. Yang, L. Huang, Q. Li, Q. Zhang, D. Li, D. Tian, D. Jiang, *J. Colloid Interface Sci.* **2023**, *652*, 813.

[44] M. Yu, J. Wang, G. Li, S. Zhang, Q. Zhong, *J. Mater. Sci. Technol.* **2023**, *154*, 129.

[45] M. Zhai, Y. Zhang, J. Xu, H. Lin, J. Wang, L. Wang, *J. Colloid Interface Sci.* **2023**, *650*, 1671.

[46] X. Zhang, K. Matras-Postolek, P. Yang, S. P. Jiang, *Carbon* **2023**, *214*, 118337.

[47] K. Wang, R.-J. You, H. Ma, T. Sun, Z.-H. He, J.-G. Chen, H. Wang, W. Wang, Y. Yang, Z.-T. Liu, *J. Colloid Interface Sci.* **2024**, *654*, 988.

[48] H. Cao, X. Zhu, J. Xue, R. Wang, J. Shang, P. Ma, C. Liu, J. Bao, *ACS Catal.* **2024**, *14*, 9734.

[49] L. Liu, J. Hu, Z. Ma, Z. Zhu, B. He, F. Chen, Y. Lu, R. Xu, Y. Zhang, T. Ma, M. Sui, H. Huang, *Nat. Commun.* **2024**, *15*, 305.

[50] W. Wang, W. Zhang, C. Deng, H. Sheng, J. Zhao, *Angew. Chem. Int. Ed.* **2024**, *63*, e202317969.

[51] H. Huang, J. Zhao, H. Guo, B. Weng, H. Zhang, R. A. Saha, M. Zhang, F. Lai, Y. Zhou, R.-Z. Juan, P.-C. Chen, S. Wang, J. A. Steele, F. Zhong, T. Liu, J. Hofkens, Y.-M. Zheng, J. Long, M. B. J. Roeffaers, *Adv. Mater.* **2024**, *36*, e2313209.

[52] J. Wang, H. Zhang, Y. Nian, Y. Chen, H. Cheng, C. Yang, Y. Han, X. Tan, J. Ye, T. Yu, *Adv. Funct. Mater.* **2024**, 2406549. https://doi.org/10.1002/adfm.202406549

[53] L. Li, H. Liu, C. Cheng, X. Dai, F. Chen, J. Ning, W. Wang, Y. Hu, *ACS Catal.* **2024**, *14*, 10204.

[54] M. Li, S. Wu, D. Liu, Z. Ye, L. Wang, M. Kan, Z. Ye, M. Khan, J. Zhang, *J. Am. Chem. Soc.* **2024**, *146*, 15538.

[55] L. Li, D. Xu, X. Xu, Z. Tian, X. Zhou, S. Yang, Z. Zhang, *Proc. Natl. Acad. Sci. U. S. A.* **2024**, *121*, e2318970121.

[56] H. Shi, Y. Liang, J. Hou, H. Wang, Z. Jia, J. Wu, F. Song, H. Yang, X. Guo, *Angew. Chem. Int. Ed.* **2024**, e202404884. https://doi.org/10.1002/anie.202404884

[57] Y. Wei, L. Chen, H. Chen, L. Cai, G. Tan, Y. Qiu, Q. Xiang, G. Chen, T.-C. Lau, M. Robert, *Angew. Chem. Int. Ed.* **2022**, *61*, e202116832.

[58] B. Ma, G. Chen, C. Fave, L. Chen, R. Kuriki, K. Maeda, O. Ishitani, T.-C. Lau, J. Bonin, M. Robert, *J. Am. Chem. Soc.* **2020**, *142*, 6188.

[59] Y. Xia, Z. Tian, T. Heil, A. Meng, B. Cheng, S. Cao, J. Yu, M. Antonietti, *Joule* **2019**, *3*, 2792.

[60] R. Kuriki, M. Yamamoto, K. Higuchi, Y. Yamamoto, M. Akatsuka, D. Lu, S. Yagi, T. Yoshida, O. Ishitani, K. Maeda, *Angew. Chem. Int. Ed.* **2017**, *56*, 4867.

[61] H. Liu, Z. Zhang, J. Meng, J. Zhang, *Mol. Catal.* **2017**, *430*, 9.

[62] A. Bafaqeer, M. Tahir, N. A. S. Amin, *Appl. Catal. B* **2019**, *242*, 312.

[63] P. Huang, J. Huang, S. A. Pantovich, A. D. Carl, T. G. Fenton, C. A. Caputo, R. L. Grimm, A. I. Frenkel, G. Li, *J. Am. Chem. Soc.* **2018**, *140*, 16042.

[64] W.-K. Jo, S. Kumar, S. Tonda, *Composites, Part B* **2019**, *176*, 107212.

[65] Y. Xu, X. Jin, T. Ge, H. Xie, R. Sun, F. Su, X. Li, L. Ye, *Chem. Eng. J.* **2021**, *409*, 128178.

[66] J.-w. Gu, R.-t. Guo, Y.-f. Miao, Y.-z. Liu, G.-l. Wu, C.-p. Duan, W.-g. Pan, *Energy Fuels* **2021**, *35*, 10102.

[67] H. Qin, R.-T. Guo, X.-Y. Liu, W.-G. Pan, Z.-Y. Wang, X. Shi, J.-Y. Tang, C.-Y. Huang, *Dalton Trans.* **2018**, *47*, 15155.

[68] J. Fu, L. Zhu, K. Jiang, K. Liu, Z. Wang, X. Qiu, H. Li, J. Hu, H. Pan, Y.-R. Lu, T.-S. Chan, M. Liu, *Chem. Eng. J.* **2021**, *415*, 128982.

[69] a) H. Che, X. Gao, J. Chen, J. Hou, Y. Ao, P. Wang, *Angew. Chem. Int. Ed.* **2021**, *60*, 25546; b) Q. H. Thi, P. Man, L. Huang, X. Chen, J. Zhao, T. H. Ly, *Small Sci.* **2023**, *3*, 2200099.

[70] G. Azimi, R. Dhiman, H.-M. Kwon, A. T. Paxson, K. K. Varanasi, *Nat. Mater.* **2013**, *12*, 315.
